# De novo identification of maximally deregulated subnetworks based on multi-omics data with DeRegNet

**DOI:** 10.1186/s12859-022-04670-6

**Published:** 2022-04-19

**Authors:** Sebastian Winkler, Ivana Winkler, Mirjam Figaschewski, Thorsten Tiede, Alfred Nordheim, Oliver Kohlbacher

**Affiliations:** 1 Applied Bioinformatics, Department of Computer Science, University of Tuebingen, Tübingen, Germany; 2 International Max Planck Research School (IMPRS) “From Molecules to Organism”, Tübingen, Germany; 3 Interfaculty Institute for Cell Biology (IFIZ), University of Tuebingen, Tübingen, Germany; 4 German Cancer Consortium (DKTK), German Cancer Research Center (DKFZ), Heidelberg, Germany; 5 Leibniz Institute on Aging (FLI), Jena, Germany; 6 Institute for Bioinformatics and Medical Informatics, University of Tuebingen, Tübingen, Germany; 7 Translational Bioinformatics, University Hospital Tuebingen, Tübingen, Germany

**Keywords:** Biomolecular networks, Fractional integer programming, De-novo subnetwork enrichment, Functional enrichment, Omics data

## Abstract

**Background:**

With a growing amount of (multi-)omics data being available, the extraction of knowledge from these datasets is still a difficult problem. Classical enrichment-style analyses require predefined pathways or gene sets that are tested for significant deregulation to assess whether the pathway is functionally involved in the biological process under study. *De novo* identification of these pathways can reduce the bias inherent in predefined pathways or gene sets. At the same time, the definition and efficient identification of these pathways *de novo* from large biological networks is a challenging problem.

**Results:**

We present a novel algorithm, DeRegNet, for the identification of maximally deregulated subnetworks on directed graphs based on deregulation scores derived from (multi-)omics data. DeRegNet can be interpreted as maximum likelihood estimation given a certain probabilistic model for de-novo subgraph identification. We use fractional integer programming to solve the resulting combinatorial optimization problem. We can show that the approach outperforms related algorithms on simulated data with known ground truths. On a publicly available liver cancer dataset we can show that DeRegNet can identify biologically meaningful subgraphs suitable for patient stratification. DeRegNet can also be used to find explicitly multi-omics subgraphs which we demonstrate by presenting subgraphs with consistent methylation-transcription patterns. DeRegNet is freely available as open-source software.

**Conclusion:**

The proposed algorithmic framework and its available implementation can serve as a valuable heuristic hypothesis generation tool contextualizing omics data within biomolecular networks.

## Introduction

Modern high-throughput technologies, in particular massively parallel sequencing [1] and high-resolution mass spectrometry [2], enable omics technologies, i.e. the determination of bioanalytes on the genome-wide scale. Many of of these omics technologies are increasingly being applied in clinical settings and publicly available large-scale data resources such as The Cancer Genome Atlas (TCGA) [3] provide ample opportunity for research. These resources can provide valuable reference data sets in the analysis of molecular profiles of individual patients and patient groups. However, one of the biggest challenges in the analysis of omics data remains functional annotation/interpretation. The interpretation of the experimental read-outs with the goal of understanding the underlying known or unknown biological processes and functions is a vital step in providing personalized, precise, and focused molecular therapies.

One of the most widely used approaches for functional annotation of large omics datasets is gene set enrichment (GSE) [4]. In its most basic form, GSE entails hypergeometric and Fisher test-based approaches to detect the overrepresentation of differentially expressed genes. GSE requires a set of predefined gene sets (typically obtained from pathway databases [5] such as KEGG [6], WikiPathways [7] or Reactome [8]) and a measure of “deregulation” (e.g., a binary indication of differential gene expression). The goal of the GSE analysis is to identify those gene sets from the collection which show “high” deregulation. Here, the term “high” is defined by the method’s specific underlying statistical model. In the simplest case, the method examines if each gene set contains a higher number of differentially expressed genes than would be expected by chance, under the assumption that differentially expressed genes are represented uniformly across all genes. Many adaptations and variations of GSE exist [4, 9].

Classical GSE methods treat pathways as an unstructured collection of genes and do not explicitly account for the extensive biological knowledge encoded in biological networks. Networks as an abstraction for biological knowledge can be represent signaling networks, metabolic networks [10], gene regulatory networks [11], or protein-protein interaction networks [12, 13], and more.

There has been extensive research into the possibility of designing enrichment methods which take into account the topology of the pathways [14–16]. An example of such approach is the calculation of topology-dependent perturbation scores for each gene [17]. A further aspect usually ignored by GSE methods is the issue of pathway crosstalks. While ’textbook pathways’ have a solid base in biological findings and can provide useful guidance for functional interpretation of omics experiments, molecular and cellular events are often more complicated and involve the direct interaction of molecular entities across predefined pathway boundaries. Correspondingly, a range of methods were proposed which aim to extract “deregulated” patterns from larger regulatory networks without relying on predefined pathways [18, 19]. These methods are often referred to as *de novo* pathway enrichment (de novo pathway identification, de novo subnetwork/subgraph enrichment/identification/detection) methods, emphasizing that the pathways are defined/extracted from the data itself and are not given as fixed gene sets. Here, we also call algorithms of this flavor deregulated subnetwork/subgraph detection/identification/enrichment methods.

A way to categorize these methods is based on how they handle undirected or directed interaction networks. A lot of biomolecular interactions are directed in nature, e.g. protein A phosphorylates protein B, enzyme A precedes enzyme B in a metabolic pathway in contrast to symmetric interactions such as physical interactions of proteins in protein complexes.

Some methods designed for undirected networks are described in the following studies: [20–31]. More detailed review of these method is available in [19]. These methods, while achieving similar results on an abstract level, vary greatly in terms of suitable underlying networks, interpretation of outcomes and algorithmic strategies employed. Algorithmic approaches employed include ant colony optimization [31], dynamic programming [27], simulated annealing [20], integer programming [23, 24], Markov random fields [32] or message passing approaches [28].

Also, some methods are tailored to the characteristics of a particular data type. An example are methods attempting to find significantly mutated pathways/networks [33–38], trying to factor in the pecularities of mutation data in a network context.

While methods which work natively with directed networks are rarer [39–44], it is instrumental to be able to capture the effects of directed biomolecular interactions in the process of discovering deregulated networks. One particular approach is the one described in [40] which utilized an integer programming approach in order to find deregulated subnetworks. It uncovers deregulated subnetworks downstream or upstream of a so called root node where the latter can be fixed *a priori* or determined by the algorithm itself.

In this paper, we present an algorithm for de novo subnetwork identification which can conceptually be characterized as a mixture of the approach presented by [40] and the prize-collecting Steiner tree methods proposed in [45–48]. Our method natively handles directed interaction networks and adapts from [40] the general integer programming approach in such a way that it can encapsulate the general idea of sources and targets as put forward in the prize-collecting Steiner tree/forest (PCST/PCSF) approaches [45–48] which capture the idea of deregulated networks starting or ending at certain types of nodes, for example membrane receptors and transcription factors. Methodologically, we extend the integer programming approach of [40] (*Backes et al.*) to fractional integer programming to allow for the necessary flexibility to incorporate sources and targets. Furthermore, we show that our algorithm, DeRegNet, can be interpreted as maximum likelihood estimation under a certain natural statistical model. We demonstrate DeRegNet’s suitability as an exploratory hypothesis generation tool by applying it to TCGA liver cancer data. We introduce a personalized approach to interpreting cancer data and introduce the notion of network-defined cancer genes which allow to identify patient groups based on their similarity of their detected personalized subgraphs. The appendix Additional file 1: Supplementary Material and Methods furthermore contains a demonstration of the usefulness of subgraph-derived features for survival prediction. In particular, these features outperform comparable features derived from gene set enrichment indicated pathways and also improve classifiers based on clinical data alone.

**Fig. 1:**
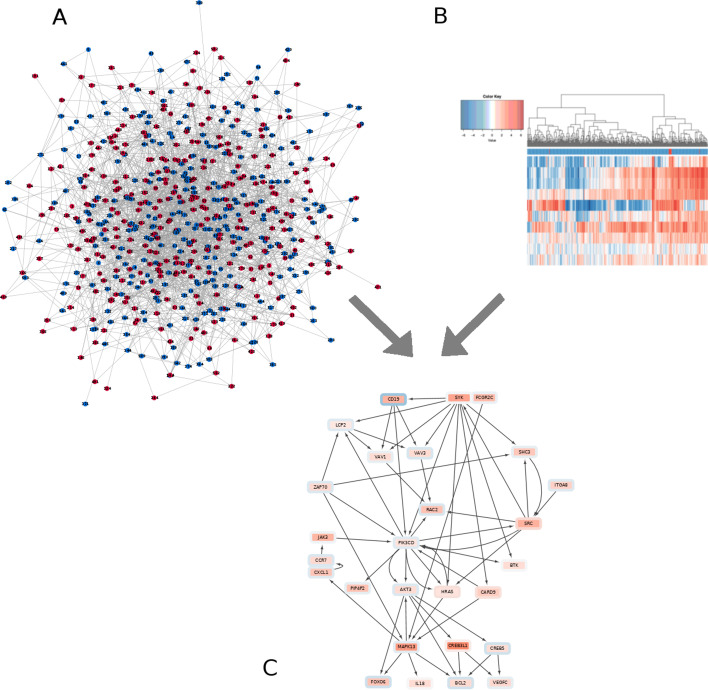
DeRegNet’s inputs are a biomolecular network (**A**), such as a signaling or gene regulatory network, and omics measurements (**B**), such as gene expression data. The latter are mapped onto the nodes of the network acting as node-level measures of deregulation. DeRegNet then extracts the most deregulated subnetwork (**C**) from the larger regulatory network according to some definition of *most deregulated*. For a conceptual view of the progression from set enrichment to de novo subnetwork methods we refer to Fig. 2

**Fig. 2:**
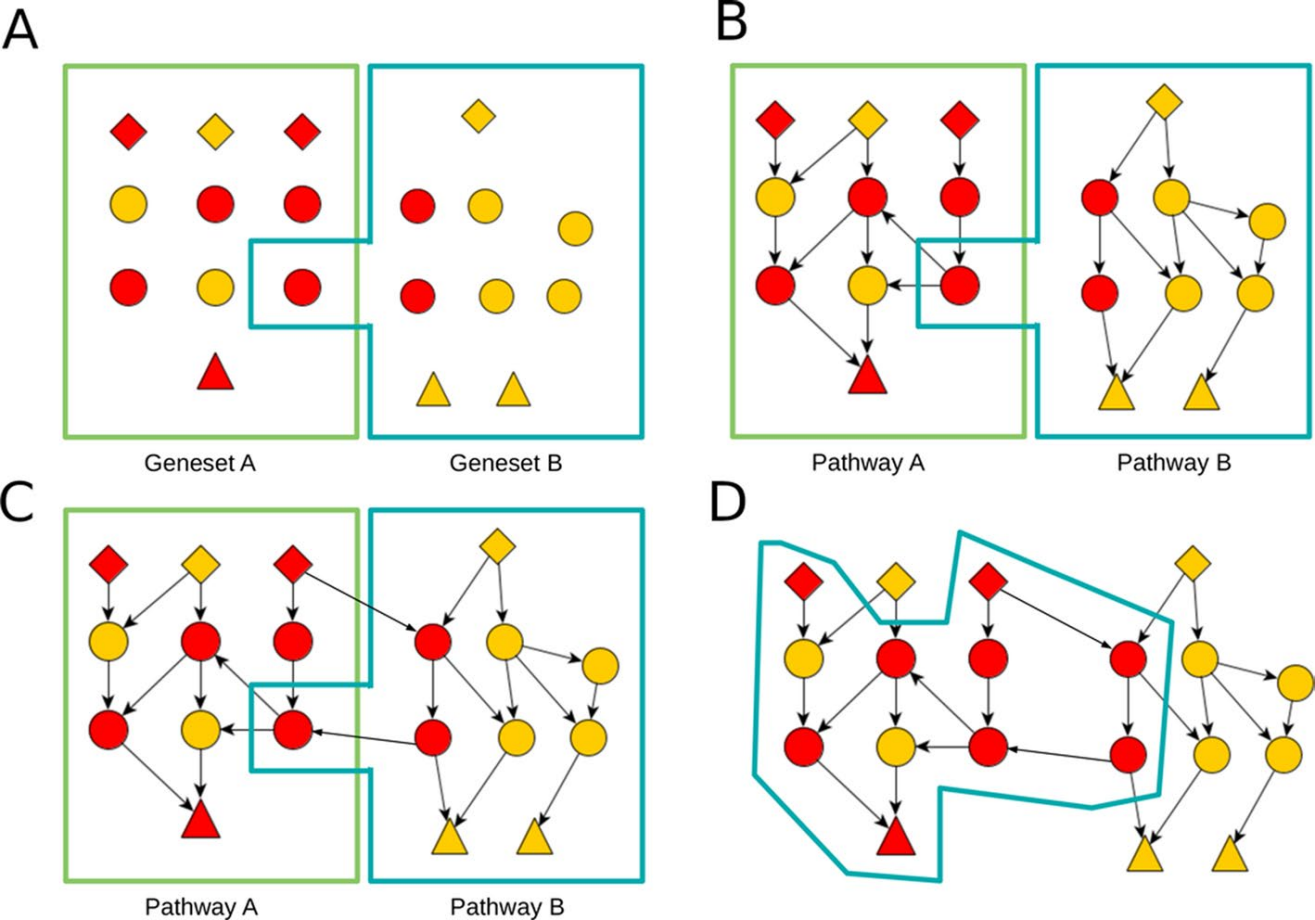
Conceptual progression from gene set enrichment to de-novo subnetwork enrichment. **A** Classical gene set enrichment. See Additional file 2: Fig. S1 for additional details. **B** Topological pathway enrichment. See Additional file 2: Fig. S2 for additional details. **C** Topological pathway enrichment with pathway cross-talk. See Additional file 2: Fig. S3 for additional details. **D** De-novo subnetwork/pathway enrichment like DeRegNet. See Additional file 2: Fig. S4 for additional details.

## Methods and materials

### DeRegNet: a de-novo subnetwork identification algorithm

#### Formal setting and definitions

Formally, it is given a directed graph $G=(V,E)$, i.e. $E \subset V \times V$, representing knowledge about biomolecular interactions in some way. To avoid certain pathologies in the models defined below, it is assumed that *G* has no self-loops, i.e. $(v,v) \notin E$ $\forall v \in V$. For a subset $S \subset V$, one defines $\delta ^{+}(S) = \{u \in V \backslash S: \exists v \in S: (v,u) \in E\}$ and $\delta ^{-}(S) = \{u \in V \backslash S: \exists v \in S: (u,v) \in E\}$, i.e. the sets of outgoing nodes from and incoming nodes into a set of nodes *S*. For a node $v \in V$ one writes $\delta ^{\pm }(v) := \delta ^{\pm }(\{v\})$. Furthermore, it is given a score function $s:V \rightarrow {\mathbb {R}}$, describing some summary of experimental data available for the biomolecular entities represented by the nodes. For a given graph $G=(V,E)$ any node labeling function $f:V \rightarrow {\mathbb {R}}$ is implicitly implied to be a vector $f \in {\mathbb {R}}^{|V|}$, subject to an arbitrary but fixed ordering of the nodes (shared across all node labeling functions). In particular, with $f_v := f(v)$ for $v \in V$, given $f, g:V \rightarrow {\mathbb {R}}$, one can write $f^Tg = \displaystyle \sum _{v \in V} f_v g_v$. For $S \subset V$ and $f:V \rightarrow {\mathbb {R}}$ one defines $f_S:V \rightarrow {\mathbb {R}}$ via $f_S(v) := 0$ for all $v \in V \setminus S$ and $f_S(v) := f(v)$ for all $v \in S$. Defining $e:V \rightarrow {\mathbb {R}}$ with $e(v) := 1$ for all $v \in V$, one further can write $e^T_S f= \displaystyle \sum _{v \in S} f_v$ for $S \subset V$ and $f:V \rightarrow {\mathbb {R}}$. Comparison of node labeling functions *f*, *g* are meant to be understood element-wise, e.g. $f \le g$ means $f_v \le g_v$ for all $v \in V$. Apart from the graph *G* and node scores *s*, there are given possibly empty subsets of nodes $R \subset V$ and $T \subset V$. It is referred to *R* as *receptors* (or sometimes *sources*) and to *T* as *terminals* (or sometimes *targets*), independent of the biological semantics underlying the definition of these sets (see below). For enforcing the topology of the subnetworks later on, strongly connected components will play a decisive role and it is said that a subset of nodes $V' \subset V$ induces a strongly connected subgraph ($V'$ *iscs*, for short) if the subgraph induced by $V'$ is strongly connected.

#### Probabilistic model for deregulated subgraphs

The mathematical optimization model which is at the heart of the DeRegNet algorithm and presented in the next subsection amounts to maximum likelihood estimation under a certain canonical statistical model. The model assumes binary node scores $s:V \rightarrow \{0,1\}$ which are realizations of random variables ${\mathbf {S}} = (S_v)_{v \in V}$. Here, $S_v = 1$ is interpreted as node $v \in V$ being *deregulated*. Further it is assumed the existence of a subset of vertices $V' \subset V$ such that $S_v | v \in V' \sim Ber(p')$ and $S_v | v \in V \backslash V' \sim Ber(p)$ with $p, p' \in (0,1)$ denoting probabilites of deregulation outside and inside of the deregulated subgraph encoded by $V'$ respectively. It is assumed that $p' > p$ to reflect the idea of *higher* deregulation (probability) in the *deregulated* subgraph, whereas *p* represents a certain amount of background deregulation. The network context (dependency) is introduced via the restriction that $V' \in {\mathcal {C}}(V) \subset {\mathcal {P}}(V)$. Here, ${\mathcal {P}}(V)$ is the power set of *V* (the set of subsets of *V*) while ${\mathcal {C}}(V)$ as a subset of ${\mathcal {P}}(V)$ represents the set of feasible substructures and should (can) reflect topologies inspired by known biomolecular pathway topologies like the one described in Backes et al. [40] and the next subsection. Furthermore it is assumed, that the $(S_v)$, given a network context and deregulation probabilities $p, p'$, are independent. We show in the appendix that under this model and the constraints given by the fractional integer programming problem formulated in the next subsection (defining ${\mathcal {C}}(V)$ in the above notation) DeRegNet amounts to maximum likelihood estimation. Furthermore, we also show that the model put forward in Backes et al. [40] amounts to maximum likelihood estimation only under the assumption of a fixed subgraph size.

#### Finding deregulated subgraphs by fractional integer programming

Given the definitions of the preceding sections, we can now formulate the main model underlying DeRegNet. The DeRegNet model and also the model of Backes et al. [40] can be placed in the context of the so called *Maximum Weight Connected Subgraph Problem (MWCSP)*, see Additional file 1: Supplementary Material and Methods. Note that in the following we formulate all problems as maximization problems and minimization may, depending on the semantics of the node score, be the proper choice. Minimization may for example be prudent in case the node scores represent p-values originating from some statistical significance test. As Backes et al. [40] we model the problem of finding deregulated subnetworks in terms of indicator variables $x_v = {\mathbf {I}}(v \in V')$ and $y_v = {\mathbf {I}}(v \text { is the root node})$ where $V' \subset V$ is a set of nodes inducing a subgraph such that one can reach every node in that subgraph by means of a directed path from the root node. Here, ${\mathbf {I}}(P) = 1$ if *P*, ${\mathbf {I}}(P) = 0$ if not *P* for some predicate *P*. In addition the root is supposed to be a source node and all nodes in the subgraph with no outgoing edges are supposed to be terminal nodes. The proposed model then reads like this:

1a $$\begin{aligned}&\mathop {\text {max}}\limits _{x, y\in \{0,1\}^V}\quad {\dfrac{s^Tx}{e^Tx}} \end{aligned}$$

1b $$\begin{aligned}&s.t. \qquad {y \le x} \end{aligned}$$

1c $$\begin{aligned}&{e^Ty = 1} \end{aligned}$$

1d $$\begin{aligned}&{k_{min} \le e^Tx \le k_{max}} \end{aligned}$$

1e $$\begin{aligned}&{x_v - y_v - e_{\delta ^{-}(v)}^Tx \le 0 \,\,\,\, \forall v\in V} \end{aligned}$$

1f $$\begin{aligned}&{e_S^T(x-y) - e_{\delta ^{-}(S)}^Tx \le |S|-1 \,\,\,\,\forall S\subset V \,iscs,\,|S| > 1} \end{aligned}$$

1g $$\begin{aligned}&{y_v = 0 \,\,\,\, \forall v \in V \setminus R \,\,\,\text { if } R \ne \varnothing } \end{aligned}$$

1h $$\begin{aligned}&{x_v - e_{\delta ^{+}(v)}^Tx \le 0 \,\,\,\, \forall v \in V \setminus T \,\,\,\text { if } T \ne \varnothing } \end{aligned}$$

1i $$\begin{aligned}&{e_\mathbf{Inc }^Tx = |\mathbf{Inc} |} \end{aligned}$$

1j $$\begin{aligned}&{e_\mathbf{Ex }^Tx = 0} \end{aligned}$$

The model derives from the corresponding integer linear programming model in [40] and adapts it for the fractional case, most notably here are the constraints involving the receptors *R* (1g) and the terminals *T* (1h). (1g) just ensures that the root node is a receptor while (1h) ensures that any node in the subgraph with no outgoing edges is a terminal node. (1b) means that a node can only be the root if it is included in the subgraph, (1c) means that there is exactly one root, (1d) means that the size of subgraph has to be within the bound given by $k_{min}, k_{max} \in {\mathbb {N}}$, (1e) says that a node $v \in V$ in the subgraph is either the root node or there is another node $u \in V$ in the subgraph such that there is an edge $(u,v) \in E$. Moreover, the constraints (1i) and (1j) trivially allow to include and exclude specific nodes from given node sets $\mathbf{Inc} \subset V$ and $\mathbf{Ex} \subset V$ respectively. In many situations specific nodes, i.e. genes in the case of gene regulatory networks, may be of interest in other topological positions than in a receptor or terminal role. In that case just requiring a certain gene to be part of the subgraph without any special constraints on its inclusion in topological terms can be of value. The constraint (1f) is the most involved one and actually describes exponentially many constraints which ensure that there are no disconnected directed circles [40] by requiring that any strongly connected component in the subgraph either contains the root node or has an incoming edge from another node which is part of the subgraph but not part the given strongly connected component. Finally, the objective (1a) describes the notion of maximizing the average score of the subgraph. This is crucial for allowing the model the flexibility to connect source nodes to target nodes and also is at the heart of DeRegNet being able to do Maximum Likelihood estimation given the presented statistical model. We summarize some crucial terminology next, before proceeding in the next subsection to describe the solution algorithms for DeRegNet.

##### Definition 1

(*DeRegNet instances, data, and subgraphs*) A tuple (*G*, *R*, *T*, *Ex*, *Inc*, *s*) is called an instance of DeRegNet (a DeRegNet instance, an instance of the DeRegNet model). Here, $G=(V,E)$ is the underlying graph, $R \subset V$ is the receptor set, $T \subset V$ is the terminal set, ${Ex} \subset V$ is the exclude set, ${Inc} \subset V$ is the include set and $s: V \rightarrow {\mathbb {R}}$ is the node score (the score). Further, $x_v: V \rightarrow \{0,1\}$ is called a subgraph with the understanding that it is referred to the subgraph of *G* induced by $V^* = \{v \in V: x_v = 1\}$. Equivalently to $x_v: V \rightarrow \{0,1\}$, it is also referred to the corresponding $V^* = \{v \in V: x_v = 1\}$ as a subgraph. A subgraph is feasible for DeRegNet (for the DeRegNet instance), if it satisfies DeRegNet’s constraints (1b-j). A subgraph satisfying these constraints is called a feasible subgraph. A feasible subgraph which optimizes problem (1) is called an optimal subgraph.

**Fig. 3:**
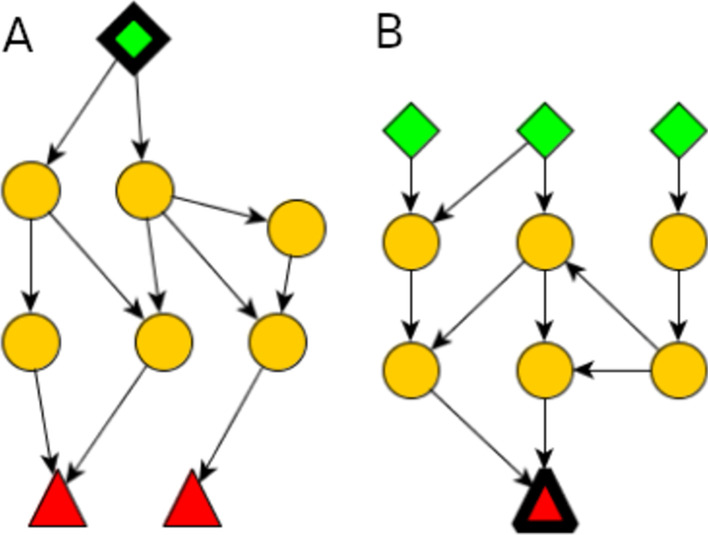
Conceptual view of subgraphs extracted by DeRegNet. **A** From a receptor node/root node (green cube) one can reach any node in the subnetwork. Nodes without any edges leading to other nodes (red triangles) of the subnetwork need to be elements of the so called terminal nodes. Generally, all nodes in the subgraph can be reached from the root node. **B** By reversing the orientation of the underlying network before applying DeRegNet, one can find subgraphs with only one terminal “root” node and multiple receptor nodes such that the terminal node can be reached from any other node in the subgraph. See Additional file 1: Supplementary Material and Methods for further details on applying DeRegNet in reverse mode


Some formal properties of DeRegNet and its solutions can be found in the Additional file 1: Supplementary Material and Methods. A high-level depiction of the overall logic of DeRegNet can be found in Fig. 1 while an overview of DeRegNet’s position within the range of functional enrichment methods is conceptually depicted in Fig. 2. A conceptual view of the particular types of subgraphs determined by DeRegNet can be seen in Fig. 3 whereas the high-level algorithm of DeRegNet is summarized by Algorithm 1.

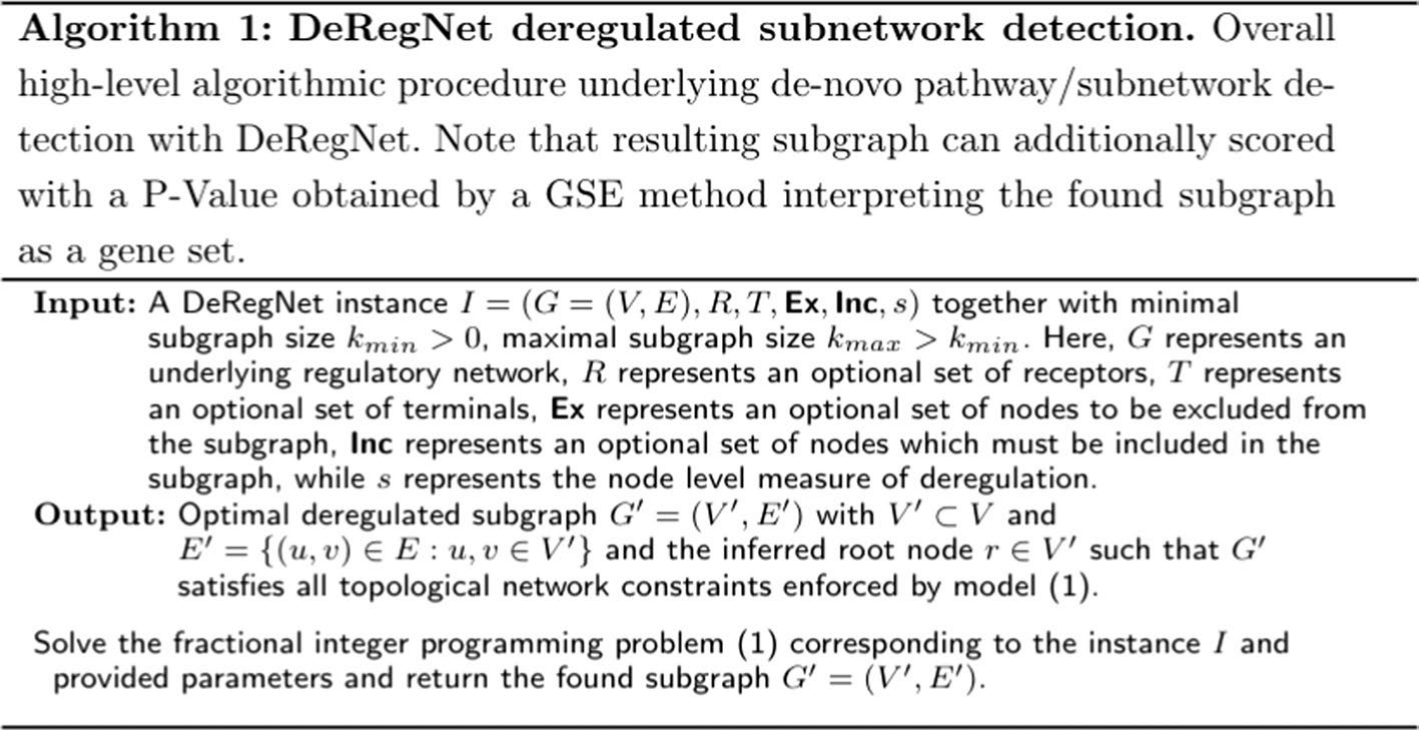


#### Solving the fractional integer programming model

We solve the integer fractional linear programming problems introduced in the previous sections by one out of two implemented methods. Firstly, a generalization of the Charnes–Cooper transformation [49] for fractional linear programs described by [50] and secondly an iterative scheme as introduced generally by Dinkelbach [51, 52] and subsequently applied in the context of integer fractional programming by [53, 54]. While the Dinkelbach-type algorithm solves the problem by iteratively solving certain non-fractional versions of the original problem until some convergence criterion is met, the generalization of the Charnes–Cooper method is based on reformulation of the entire fractional model to a quadratic problem and requires subsequent linearization of artifically introduced quadratic constraints. The latter is implemented in terms of the methods described by [55–57].

As in [40] the exponentially many constraints forbidding any strongly connected components not containing the root and with no incoming edges are handled by lazy constraints. Every time an integer solution is found the Kosaraju-Sharir algorithm [58] is employed (as implemented by the Lemon graph library) to check for violating components and, in the case of violating components, the corresponding constraints are added to the model. Both solution approaches, the generalized Charnes–Cooper method and the Dinkelbach-type algorithm, allow for the lazy constraints to be handled in terms of the original formulation since both retain the relevant variables of the model within the transformed model(s).

For more details on the theoretical underpinnings and the practical implementation of DeRegNet’s solution algorithms consult Additional file 1: Supplementary Material and Methods.

### Assessment of inference quality for known ground truths

The evaluation and benchmarking of *de novo* pathway enrichment or deregulated subnetwork detection algorithms and implementations remains a big challenge. While many of the methods cited in the introduction can be applied to reveal useful biological insight, there are limited studies concerning the comparison of formal and statistical properties of the methods. The two main obstacles are a lack of well-defined gold standard datasets as well as the differences concerning the exact output of the methods. For example, it is not immediately clear how to compare algorithms which produce undirected subnetworks to those which elicit directed networks of a certain structure. An important first step toward atoning the issue in general is described in [19] which focuses on benchmarking approaches for undirected networks. For the purposes of this paper, we designed and performed benchmarks of DeRegNet relative to its closest relative, namely the algorithm described in [40], henceforth referred to as *Backes et al.*. Note however, while we are comparing the integer programming based algorithm of Backes et al. to the fractional integer programming algorithm of DeRegNet, we are using the former as implemented in the DeRegNet software package. This renders the benchmark less dependent on implementation technology since both algorithms have been implemented with the same general stack of languages and libraries. For the benchmark we always utilize the human KEGG network as the underlying regulatory network. We then repeatedly simulate subgraphs which match the structure of both models (DeRegNet and Backes et al.). The simulation procedure is described more formally in Additional file 1: Supplementary Material and Methods. Initially, the simulated subgraph consists of one randomly selected root node, to which we iteratively add a random “outgoing” neighbor of a randomly selected current node in the subgraph until the size of the subgraph matches a randomly chosen value. The latter is uniformly chosen to be an integer between a given lower and an upper bound. “Outgoing” neighbors of $v \in V$ are any nodes from the set $\delta ^{+}(v) = \{u \in V \backslash \{v\}: (v,u) \in E\}$. All nodes in the simulated “real” subgraph are assigned a node score of 1 with a certain probability $p' > 0$, while all nodes which are not contained in the subgraph are assigned a node score of 1 with probability *p* where $0< p < p'$. In summary, we obtain random “real” subgraphs and simulated scores where the latter reflect the different likelihood of a node being deregulated given whether it is contained in the subgraph or not. In terms of the probabilistic interpretation of DeRegNet presented above, the simulation scheme corresponds directly to a deregulation probability of $p'$ for nodes in the “real” subgraph and of *p* for nodes not part of the “real” subgraph. The appendix in Additional file 1: Supplementary Material and Methods provides further details on the simulation of benchmark instances.

Given a sequence of $N \in {\mathbb {N}}$ of these simulated instances, the algorithms are run in order to find subgraphs which can then be compared to the known simulated real subgraph. Here, a *hit* (*true positive*, *tp*) is defined as a node appearing in a subgraph calculated by some algorithm which is also an element of the real subgraph. A *false positive* (*fp*) is a node which appears in a subgraph calculated by an algorithm but is not part of the real subgraph. A *false negative* is defined as a node which is part of the true subgraph but not part of the subgraph detected by an algorithm. Furthermore, we can compare the sizes of the calculated subgraphs with the size of the real subgraph. In general, given an algorithm ${\mathcal {A}}$, which on a given instance with true subgraph $V' \subset V$ finds a subgraph $V_{{\mathcal {A}}}$, one can adopt all standard evaluation metrics for predictive classification models with the understanding that nodes in $V_{{\mathcal {A}}}$ are *predicted positive* and nodes in $V'$ are *true positive*. Examples are the true positive rate (sensitivity) $\mathbf{TPR} := \frac{|V' \cap V_{{\mathcal {A}}}|}{|V'|}$, i.e. the number of actual hits divided by the number of possible hits, or the Jaccard index (intersection over union) $\mathbf{J } = \frac{|V' \cap V_{{\mathcal {A}}}|}{|V' \cup V_{{\mathcal {A}}}|}$. Specifically, we utilize the Matthews correlation coefficient (MCC), the F1 score, the Jaccard index, precision and sensitivity to compare subgraphs found by DeRegNet and Backes et al. The only non-standard metric we employ compares the closeness of an inferred subgraph to a real subgraph and is referred to as size efficiency $\mathbf{SE} := \frac{|V_{{\mathcal {A}}}|}{|V'|}$, i.e. the proportion of algorithm subgraph size to real subgraph size. Another comparison metric is the running time of the algorithms.

Furthermore, the benchmark is based on the realistic assumption that we do not know the exact size of the real subgraph and that one can only assume lower and upper bounds on the subgraph size instead. Since the Backes et al. algorithm does need a fixed a priori specified subgraph size we employ a strategy suggested in [40] to circumvent that fact. Namely, we iterate from the lower to the upper bound, find a subgraph for each subgraph size and then regard the union graph of all found subgraphs as the one subgraph emitted by the algorithm. DeRegNet natively requires only a lower and an upper bound on subgraph size as parameters. All benchmarks have been carried out with the following setup: software: Ubuntu 18.04, Gurobi 9.5.0, hardware: 12x Intel i7-8750H @ 4.1 GHz, 32 GB RAM, Samsung SSD 970 EVO Plus. See Additional file 1: Supplementary Material and Methods for more formal details. Finally, in order to assess the comparative advantage of deregulated subgraphs to pre-defined pathways (gene sets) we calculated GSE P-values for optimal DeRegNet subgraphs (interpreted as gene sets) based on the simulated scores, as well as for the standard KEGG gene sets and compared the distribution of subgraph P-values with those of significant KEGG gene sets across all simulation runs.

### Network and omics data

#### KEGG network

While many sources for directed biomolecular networks are available, e.g. [59], in this paper we here utilize a directed gene-level network constructed from the KEGG database [60–62] with the KEGGgraph R-package [63]. The script used to generate the network as well as the network itself can be found in the DeRegNet GitHub repository. See the subsection on Software Availability for details.

#### RNA-Seq and 450k methylation array derived node scores for TCGA-LIHC

Gene expression and methylation data was downloaded for hepatocellular carcinoma TCGA project from the Genomic Data Commons Portal (https://portal.gdc.cancer.gov/projects/TCGA-LIHC). Raw quantified RNA-Seq counts were normalized with DESeq2 [64] which was also used for calculating log2 fold changes for every gene with respect to the entire cohort. We also used DESeq2 to calculate P-Values for differential expression between tumor and control samples. Personalized log2 fold changes were calculated by dividing a patients tumor sample expression by the mean of all available control samples (adding a pseudo count of 1) before taking the log. The following node scores are defined:

- *Global RNA-Seq score* *s*: $s_v =$ RNA-Seq log2-fold change for a gene $v \in V$ as calculated by DESeq2 for the TCGA-TCGA-LIHC cohort
- *Trinary global RNA-Seq score*:
  2 $$\begin{aligned} s^t_v := {\left\{ \begin{array}{ll} +1 & s_v > 2.0 \text { and DESeq2 P-Value}< 0.05\\ -1 & s_v< 2.0 \text { and DESeq2 P-Value} < 0.05\\ 0 & \text {otherwise} \end{array}\right. } \end{aligned}$$
- *Trinary personalized RNA-Seq score* $s^c$ for case *c*:
  3 $$\begin{aligned} s_v^c := {\left\{ \begin{array}{ll} +1 & \quad \text {if } \text { personalized log2 fold} > 2\\ -1 & \quad \text {if } \text { personalized log2 fold} < -2\\ 0 & \quad \text {else} \end{array}\right. } \end{aligned}$$

From the 450k methylation array [65, 66] data available for the TCGA-LIHC cohort we derive (global) methylation node/gene scores $m_v \in \{-1,0,1\}$ for every gene $v \in V$ representing binary methylation status as follows. First signed differentially methylated probes (DMPs) were inferred using subset quantile normalization (SQN) [66] between tumor and control samples. With *signed* we express the fact, that we keep track whether the median difference between tumor and control was positive or negative. Correspondingly, $\text {median}(\beta _1, \ldots , \beta _T) - \text {median}(\beta '_1, \ldots , \beta '_C) > 0.2$ defines a upregulated DMP while $\text {median}(\beta _1, \ldots , \beta _T) - \text {median}(\beta '_1, \ldots , \beta '_C) < -0.2$ defines a downregulated DMP. Here, $\beta _1, \ldots , \beta _T \in [0,1]$ denote all beta values from tumor samples for a given array probe while $\beta '_1, \ldots , \beta '_C \in [0,1]$ denote all beta values from control samples for that same probe. From the DMPs’ metadata contained in the TCGA-LIHC 450k data one obtains a mapping from any DMP to genes to whose promoter region the DMP lies close to (up to 1500 base pairs upstream of a genes transcription start site). Any gene $v \in V$ which is indicated by at least one upregulated DMP and no downregulated DMPs is considered upregulated and we set $m_v := 1$. Any gene $v \in V$ which is indicated by at least one downregulated DMP but no upregulated DMPs is considered downregulated and we set $m_v := -1$. For genes which are not up- or downregulated we set $m_v := 0$.

### Global and personalized deregulated subgraphs

We refer to subgraphs found with the global RNA-Seq score *s* as *global subgraphs*. A global subgraph can further be subdivided as being *upregulated* or *downregulated* depending on whether the subgraphs were found by employing a maximization or minimization objective respectively. For (any) node score $s: V \rightarrow {\mathbb {R}}$ we define $|s| : V \rightarrow {\mathbb {R}}$ by $|s|(v) := |s(v)|$ for all $v \in V$. *Dysregulated* global subgraphs are those which were found by using the score |*s*| under a maximization objective. Similarily subgraphs found with any of the scores $s^c$ with a maximization objective are called *upregulated* while those found with minimization objective are called *downregulated* (personalized subgraphs for case/patient *c*). Subgraphs found with a $|s^c|$ score under maximization are called *dysregulated* (personalized subgraphs for case/patient *c*). Any of the above subgraph types is called a *deregulated* subgraph. Subgraphs were inferred with minimal subgraph size of $k_{min} = 10$ and maximal subgraph size of $k_{max} = 50$ as this represents a reasonable range of expected pathway sizes, compare Additional file 2: Fig. S24. The optimal and four next best suboptimal global subgraphs were calculated for every modality. The subgraphs were then summarized as a subgraph of the union graph of optimal and suboptimal subgraphs in order to allow streamlined interpretation. See the supplementary figures referenced in the respective figures for references to the direct output of DeRegNet.

### Network-defined cancer genes

Genes, gene products or biomolecular agents are likely to bring about their various phenotypic effects only in conjunction with other agents via their shared biomolecular network context. By that token, one can search for genes which convey phenotypic differences by means of some defined network context. Here, we propose DeRegNet subgraphs as network context for a given case/patient in order to find genes whose inclusion into a case’s deregulated subgraph associates with a significant difference in overall survival as assessed by standard survival analysis techniques [67, 68]. Algorithm 2 describes the procedure more formally. Genes implicated by the outlined procedure are termed *network-defined cancer genes*. The next section provides details on a specific network-defined cancer gene obtained by application of the procedure to personalized upregulated subgraphs in the TCGA-LIHC cohort. 
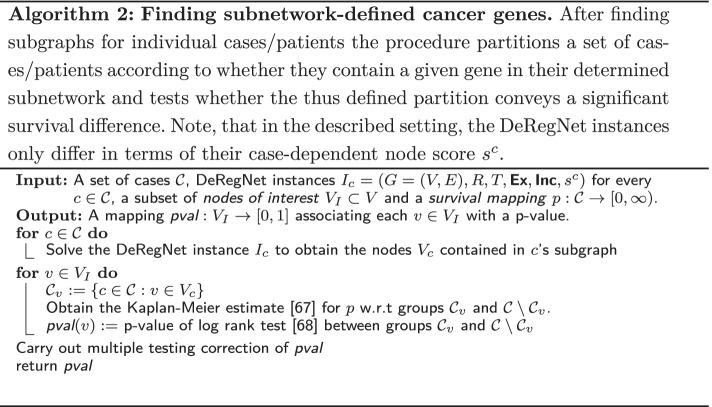


### Nodes scores representing consistent methylation and transcription patterns

In general one considers a *consistent methylation and transcription pattern* for a given gene a situation where one observes increased methylation (hypermethylation) close to/in the gene’s promoter region and decreased transcription of the gene or decreased methylation (hypomethylation) close to/in the gene’s promoter region and increased transcription of the gene [69, 70]. For a node/gene $v \in V$ we define a node score $s_v^{m\text {-}t}$ which captures these patterns by $s_v^{m\text {-}t} := {\mathbb {I}}(s^t_v \cdot m_v < 0)$, i.e.

4 $$\begin{aligned} s_v^{m\text {-}t} := {\left\{ \begin{array}{ll} +1 & \text { if } s^t_v \cdot m_v < 0\\ 0 & \text { otherwise} \end{array}\right. } \end{aligned}$$

We then infer deregulated subgraphs with nodes scores $s_v^{m\text {-}t}$ for $v \in V$ in order to capture subnetworks which consist largely of nodes which show consistent methylation-transcription patterns, thus representing de-novo pathways which may be largely regulated by epigenetic modulation of transcription.

## Results and discussion

In the following we present multiple results relating to the application of the DeRegNet algorithm. Firstly, we present benchmark results for synthetic data which compares DeRegNet to its closest methodological relative [40]. Next, we present applications of DeRegNet on a TCGA liver cancer dataset. More specifically, we present global subgraphs for the TCGA representing deregulated subnetworks summarizing the cohort under study as a whole, as well as a personalized application of DeRegNet, i.e. the derivation of patient-specific subgraphs.

### Performance comparison on data with a known ground truth

As outlined in the introduction, the field of statistical functional annotation needs adequate known ground truths (gold standards) against which one can evaluate corresponding methods, see for example [19]. Since actual ground truths are hard to come by for fundamental reasons, research for functional annotation algorithms justifiably focuses on simulated/synthetic ground truths. The latter are then generated such that they represent the assumed or postulated data-generating process. We compared DeRegNet to its closest methodological relative introduced in [40] based on simulated instances as described in *Material and Methods*.

**Fig. 4:**
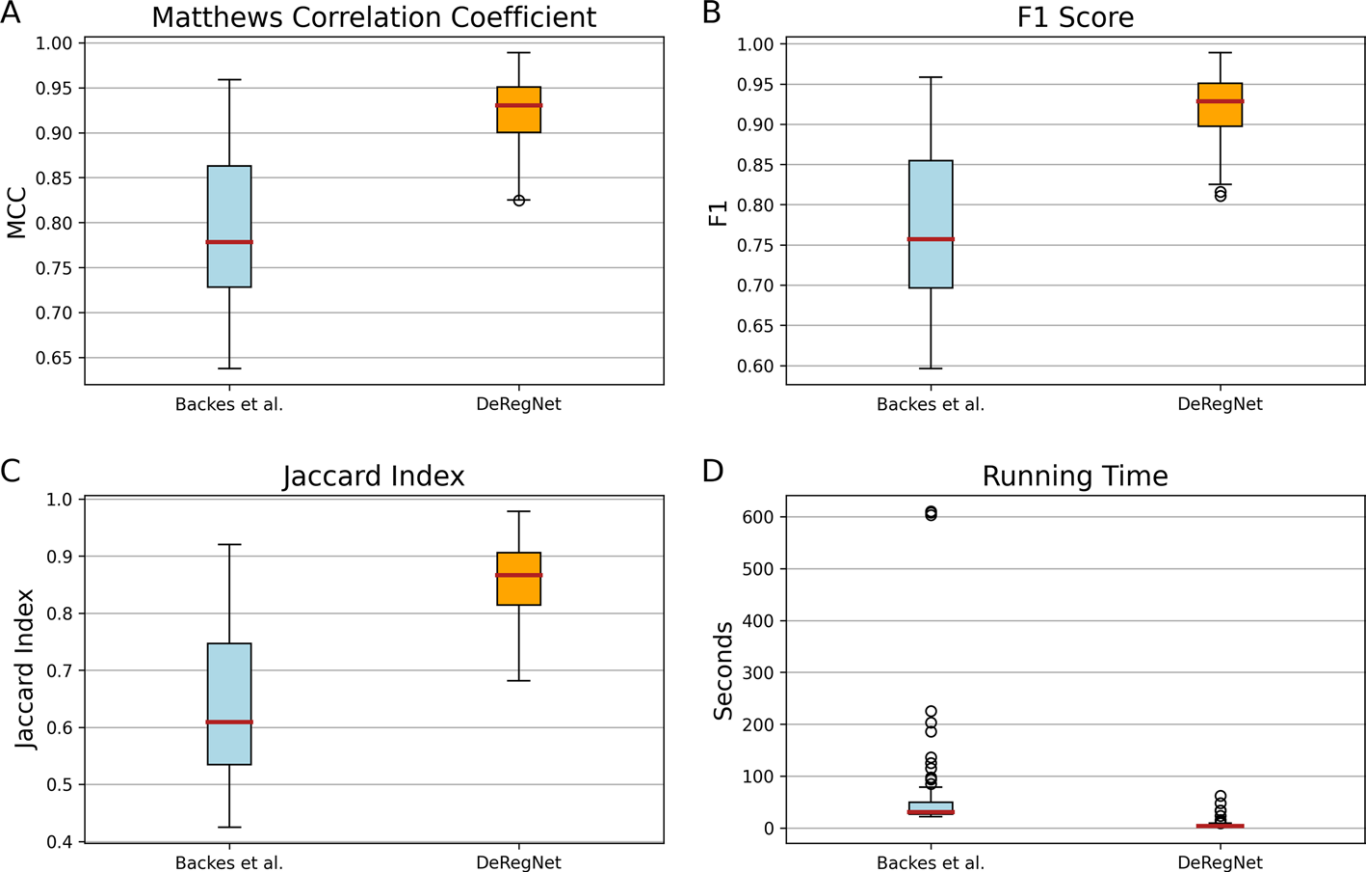
Benchmark patterns for DeRegNet and Backes et al. [40]. **A** Distributions of the Matthews correlation coefficients of DeRegNet and Backes et al. subgraphs. **B** Distributions of the F1 scores of DeRegNet and Backes et al. subgraphs. **C** Distributions of the Jaccard indices of DeRegNet and Backes et al. subgraphs. **D** Running time (in s) of DeRegNet (Dinkelbach algorithm) and $k_{max} - k_{min} + 1$ ($k_{max} = 50$, $k_{min} = 25$) runs of the Backes et al. algorithm [40]. Benchmark parameters: in-subgraph deregulation probability $p'=0.99$, out-of-subgraph deregulation probability $p = 0.01$, $k_{min} = 25$, $k_{max} = 50$, minimal size of simulated true subgraph $=30$, maximal size of simulated true subgraph $=45$, number of simulated instances $=100$, time limit $=600$ s. See Additional file 1: Supplementary Material and Methods for further formal details on the simulation procedure and Additional file 2: Figs. S18–S24 for simulation results for different parameter value settings

**Fig. 5:**
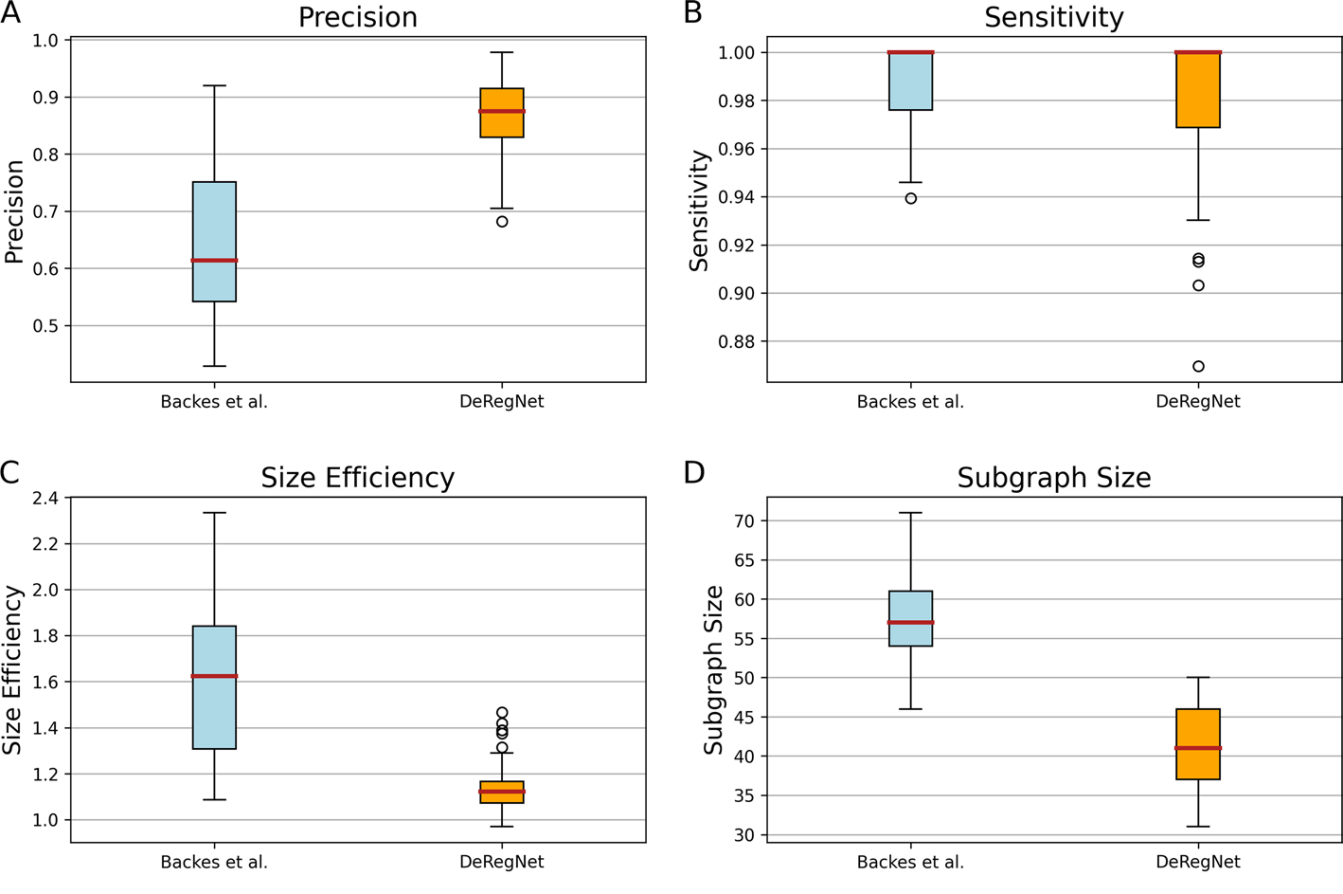
Benchmark patterns for DeRegNet and [40]. **A** Distributions of the precision of DeRegNet versus Backes et al. subgraphs. **B** Distributions of the sensitivity of DeRegNet versus Backes et al. subgraphs. **C** Distributions of the size efficiency of DeRegNet versus Backes et al. subgraphs. **D** Distributions of subgraph sizes of DeRegNet versus Backes et al. subgraphs. Benchmark parameters: As in Fig. 4. See Additional file 1: Supplementary Material and Methods for further formal details on the simulation procedure

**Fig. 6:**
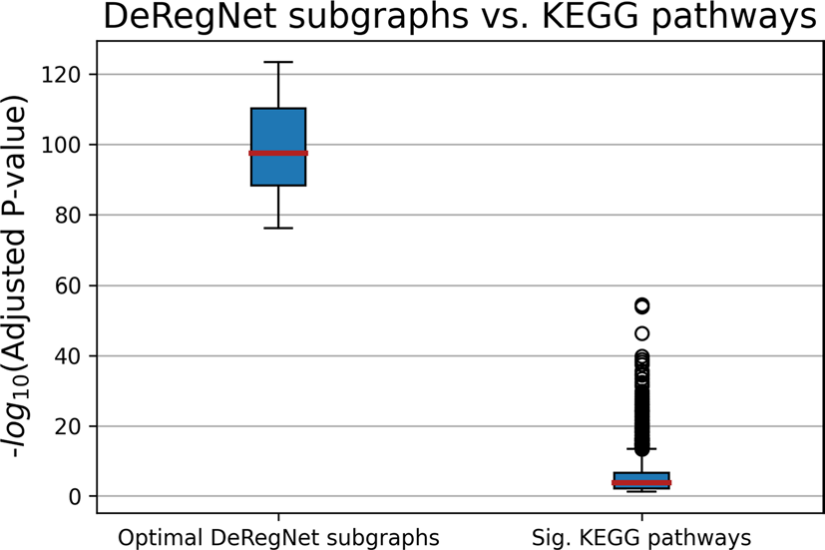
DeRegNet subgraphs versus pre-defined KEGG subgraphs. Distributions of significant *p* values for optimal deregulated subgraphs found by DeRegNet and pre-defined KEGG gene sets. Note that DeRegNet subgraphs (interpreted as gene sets) are considerably more significant than pre-defined (non data-dependent) KEGG gene sets in light of classical GSE. Additionally, all DeRegNet subgraphs were significantly enriched without exception

Figures 4 and 5 show results of simulation runs carried out according to the described procedure. As can be seen in Fig. 4, DeRegNet outperforms [40] in terms of Matthews correlation coefficient (MCC), F1 Score, Jaccard index, Precision, subgraph size efficiency (i.e. closeness to true subgraph size) and running time. Backes et al. has almost perfect sensitivity but DeRegNet generally also performs close to optimal with only a few outliers with lower sensitivity compared to Backes et al.. The Backes et al. algorithm achieves these slight sensitivity advantages with considerable cost with respect to precision as can be seen in Fig. 5. In order to assess the dependence of these simulation results on certain simulation parameters, in particular the noise level *p*, Additional file 2: Figs. S18–S23 can be consulted. For a wide range of noise settings, DeRegNet outperforms Backes et al. with the described patterns for the evaluation metrics. With increasing noise levels both algorithms start to perform less convincing.

Furthermore, all optimal subgraphs (interpreted as gene sets) found by DeRegNet (or Backes et al.) are significant w.r.t classical GSE analysis and considerably more so than pre-defined KEGG gene sets. This underlines the suitablility of de-novo subnetwork/pathway detection algorithms to find significant data-dependent “pathways” in the context of the outlined simulation studies. See Fig. 6.

Less quantitatively, note that DeRegNet allows for subgraphs which originate from so called source (root, receptor) nodes and *end* at so called terminal nodes. This is not readily possible with the Backes et al. algorithm due to the necessity to specify a fixed subgraph size *a priori* and the resulting lack of flexibility to connect receptors to targets. Also note that DeRegNet is available as open-source software and also provides an open-source implementation of the Backes et al. algorithm. Currently the implementation supports the commercial Gurobi ILP solver as a solver backend. Gurobi readily provides free academic licenses though. Furthermore, given the statistical model introduced in Material and Methods, Backes et al. solves only a special case of the maximum likelihood estimation problem which is solved by DeRegNet in its general form.

### Global deregulated subgraphs TCGA-LIHC

**Fig. 7:**
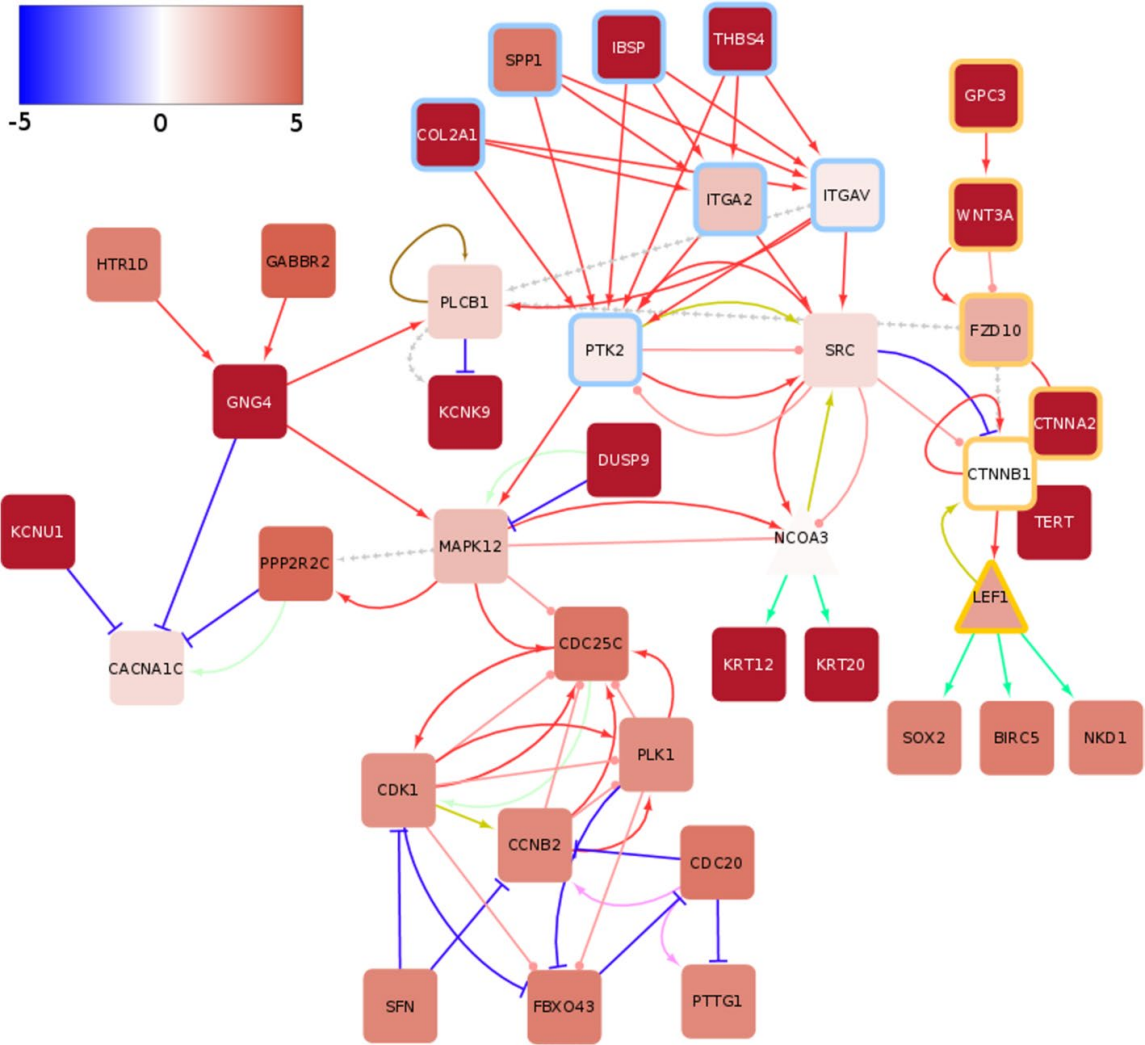
Global upregulated subgraph for TCGA-LIHC reconstructs transcriptional activation of WNT signaling. The Color of nodes indicates the average $\hbox {log}_2$ fold change of tumor samples compared to controls as represented in the color bar. The color of rims around nodes indicates genes contained in the integrin pathway (blue), the WNT pathway (yellow) and diverse other pathways (no rim). The color of edges indicates following interactions: activation (red), inhibition (dark blue), compound (brown), binding/association (yellow), indirect effect (dashed grey), phosphorylation (pink), dephosphorylation (light green), expression (green) and ubiquitination (light purple). See also Additional file 2: Figs. S6–S10

Using the DeRegNet algorithm we determined the upregulated global subgraphs obtained from running the algorithm with the global RNA-Seq score defined above. The optimal and four next best suboptimal subgraphs were calculated for every modality. The subgraphs were then summarized as a subgraph of the union graph of optimal and suboptimal subgraphs in order to allow streamlined interpretation. The global subgraph comprised of upregulated genes as nodes is shown in Fig. 7.

#### Reconstruction of transcriptional activation of WNT signaling

The subgraphs shows the activation of the WNT signaling pathway by means of over-expressed Glypican-3 (GPC3), which represents a membrane-bound heparin sulphate proteoglycan [71]. GPC3 has been extensively researched as a early biomarker and potential therapy target in HCC [72–77] (see Additional file 2: Fig. S5).

Genomic analysis conducted over the past decade have identified mutations affecting Telomere Reverse Transcriptase (TERT), $\beta$-catenin (CTNNB1) and cellular tumor antigen *p53 (TP53)* [78] as common driver mutations in HCC. Mutations in the TERT promoter are a well-studied factor in liver cancer development [79, 80] and lead to TERT overexpression while mutations in CTNNB1, activate CTNNB1 and result in activation of WNT signaling. Previous studies have determined that TERT promoter mutations significantly co-occur with CTNNB1 alternation and both mutations represent events in early HCC malignant transformation [81]. In agreement, the DeRegNet algorithm recatures the importance of a CTNNB1:TERT connection on a transcriptional level.

The subgraphs further show a possible alternative mechanism of CTNNB1 activation through upregulated GPC3, an early marker of HCC, as well as Wnt Family member 3a (WNT3A) and Frizzled 10 (FZD10). WNT3A promotes the stablization of CTNNB1 and consequently expression of genes that are important for growth, proliferation and survival [82] through activity of transcription factor Lymphoid Enhancer-Binding Factor 1 (LEF1). As shown in the subgraph Fig. 7, LEF1’s known targets SRY-box 2 (SOX2) (Sex-Determining Region Y (SRY)) and Baculoviral IAP Repeat Containing 5 (BIRC5) are likely important contributers to WNT pathway driven WNT proliferation. SOX2 is a pluripotency-associated transcription factor with known role in HCC development [83–85] and BIRC5 (survinin) is an anti-apoptotic factor often implicated in chronic liver disease and liver cancer [86–88].

In summary, our algorithm reconstructed important components of the canonical WNT signaling pathway activation in liver cancer [89–93] from TCGA-LIHC RNA-Seq data and pairwise gene-gene interaction information from KEGG.

#### Crosstalk between integrin and WNT signaling

Another interesting pattern emerging in the upregulated subgraphs is the crosstalk between the WNT signaling cascade and integrin signaling. Over-expression of Secreted Phosphoprotein 1 (SPP1) has been shown to be a common feature for most known human malignancies and it is commonly associated with poor overall survival [94]. The binding of SPP1 to integrins (e.g. integrin $\alpha$V$\beta$3) leads to further activation of kinases associated with proliferation, epithelial-mesenchymal-transition, migration and invasion in HCC, such as Mitogen Activated Kinase-like Protein (MAPK), Phosphatidylinositol-4,5-bisphosphate 3-kinase (PI3K), Protein Tyrosine Kinase (PTK2), and SRC proto-oncogene/Non-receptor tyrosine kinase (SRC) [95]. Further captured by the subgraphs is that elevated expression of PTK2 and MAPK*12* are accompanied with elevated expression of cell cycle related genes (Cell Division Cycle 25 Homolog C / M-phase inducer phosphatase 1 (CDC25C), Cyclin-dependent Kinase 1 (CDK1) and Polo-like Kinase 1 (PLK1)) thus connecting over-expression of kinases with cell proliferation.

Although KEGG lists the interaction between SRC and CTNNB1 as inhibitory in nature, other studies have concluded that activated Src enhances the accumulation of nuclear beta-catenin and therefore through their interaction contributes to an oncogenic phenotype [96].

In conclusion, the upregulated subgraphs capture the interaction of SPP1 with integrin and consequent activation of PTK2 and SRC together with their connection to the WNT signaling pathway (via CTNNB1) and cell cycle genes.

#### Downregulated oncogenes FOS and JUN and drug metabolism

**Fig. 8:**
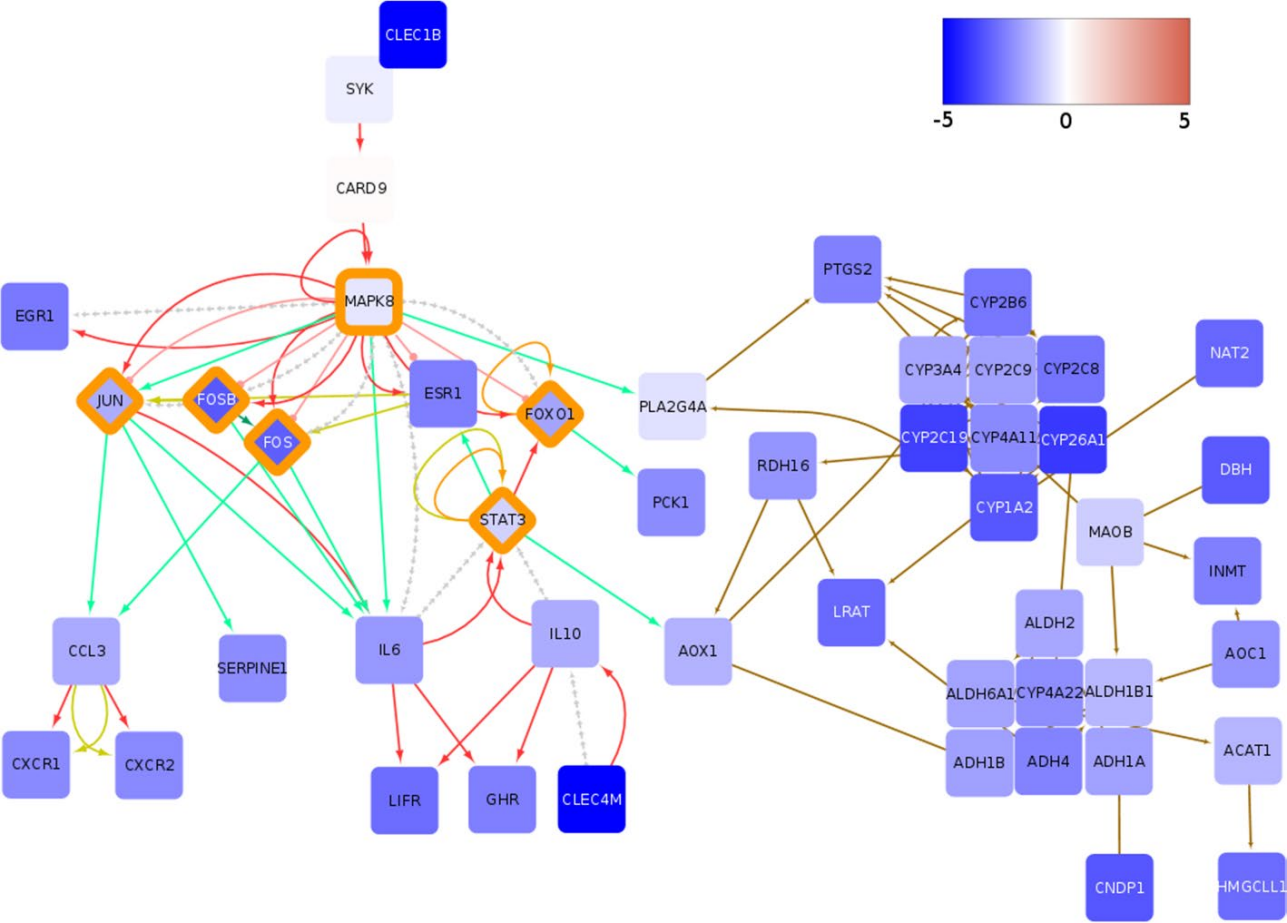
Global downregulated subgraph for TCGA-LIHC are centered on FOS and JUN transcription factors and drug metabolism. Color of nodes indicates the average $\hbox {log}_2$ fold change of tumor samples compared to controls as represented by the color bar. The color of edges indicates the following interactions: activation (red), compound (brown), binding/association (yellow), indirect effect (dashed grey) and expression (green). Also noteworthy it the general connection of transcriptional activators and inhibitors to signaling as well as metabolic networks. Transcription regulators have been highlighted with an orange rim. See also Additional file 2: Figs. S12–S16

The global downregulated subgraphs are centered around down-regulation of transcription factors FOS and JUN. The subgraph summary is depicted in Fig. 8. FOS and JUN, which form AP-1 transcription complex are considered to be oncogenic factors and necessary for development of liver tumors [97]. Considering their prominent role in liver tumorigenesis, further experimental study of the significance of Jun and Fos downregulation on HCC development could be of great interest. Interestingly, RNA-seq data show that all FOS (FOS, FOS*B*, FOS*L1*, FOS*L2*) and JUN (JUN, JUN*B*, JUN*D*) isoforms are downregulated in a majority of liver tumors of the TCGA cohort (see Additional file 2: Fig. S11 ).

Furthermore, the subgraphs show a number of downregulated Cytochrome P450 (CYP) enzymes as part of the most downregulated network of genes. CYP*3A4* is mainly expressed in the liver and has an important role in the conversion of carcinogens, such as aflatoxin $\hbox {B}_1$ toward their ultimate DNA-reactive metabolites [98], as well as, in detoxification of anticancer drugs [99]. Although the downregulation of CYP enzymes could potentially render HCC tumors sensitive to chemotherapy, liver tumors are notoriuosly irresponsive to chemotherapy [78]. Therefore, it is unclear how the gene pattern of CYP enzymes captured by the presented subgraphs could influence the HCC response to therapy and which compensatory mechanism is employed to counteract CYP downregulation.

### Personalized deregulated subgraphs for TCGA-LIHC

Finding deregulated subgraphs in a patient-resolved manner enables steps toward personalized medicine. In this section we introduce a case study where we employed our algorithm to find an upregulated subgraph for every TCGA-LIHC patient. Stratifying patients according to whether their subgraph contains a gene or not, one can identify genes whose inclusion into a patient’s inferred subgraph provides a survival handicap or advantage. Additional file 2: Fig. S17 shows the survival effect for further identified *network-defined cancer genes*. Here, we concentrate on one particular such gene, namely Spleen Tyrosine Kinase (SYK).

#### Spleen tyrosine kinase (SYK) as a network-defined cancer gene

**Fig. 9:**
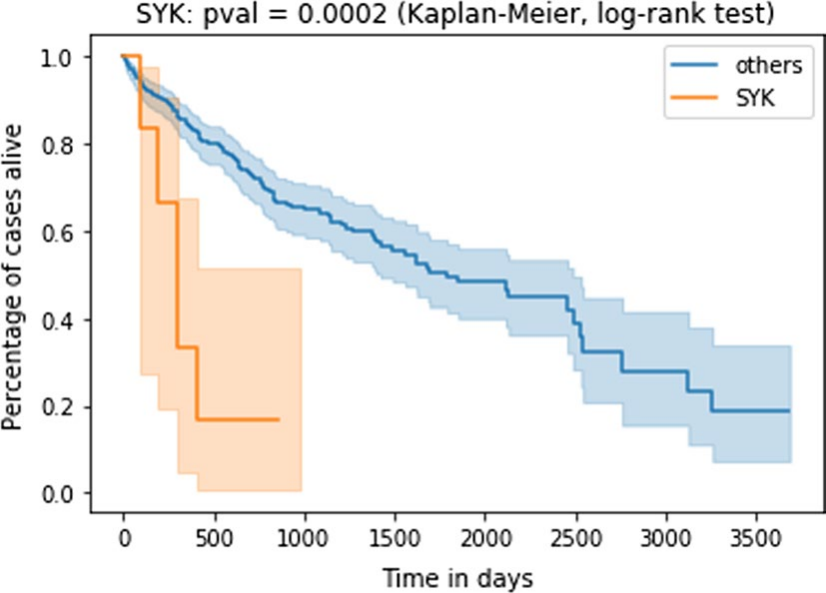
SYK signaling indicates poor survival. TCGA-LIHC cases TCGA-5C-AAPD, TCGA-CC-A3MA, TCGA-ED-A5KG, TCGA-DD-AACH, TCGA-YA-A8S7, TCGA-CC-5261, TCGA-CC-A3M9 show activated SYK signaling and poor survival. Survival difference is significant at $p=0.0002$ (Kaplan–Meier estimates and log-rank test)

**Fig. 10:**
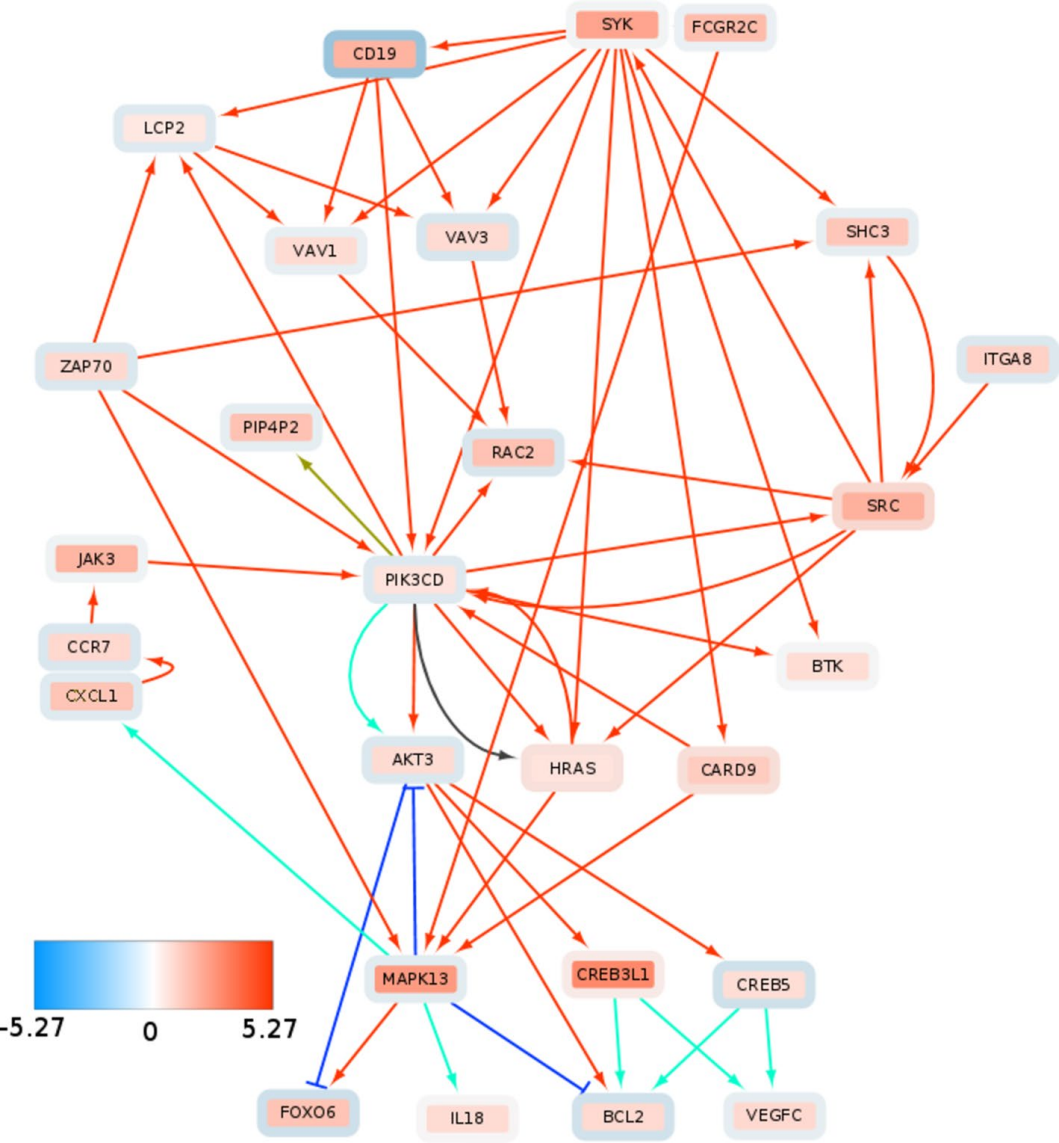
Consistent upregulation of SYK signaling components and downstream targets in subgraph of patients with poor survival. Inner node color represents the average $\hbox {log}_2$ fold change across the “SYK-positive” patients and node rim color represent average log2 fold change across the rest of the TCGA-LIHC cohort. Color of edges indicates following interactions: activation (red), inhibition (dark blue), compound (brown), indirect effect (dark grey) and expression (blue green)

Patients whose subgraph contained the spleen tyrosine kinase (SYK) showed comparatively bad survival outlook (see Figs. 9, 10).

SYK is most commonly expressed in immune cells and its deregulation has been originally associated with hematopoietic cancers [100–102]. However, it has been shown that SYK plays a role in various other cancer types and its respective roles seem to vary significantly depending on the molecular (i.e. ultimately network) context [101]. SYK comes in the form of two splice variants, SYK(*L*) and SYK(*S*) [103]. In the context of liver cancer, SYK promoter hypermethylation and corresponding SYK downregulation has been associated with poor survival [104]. Furthermore, Checkpoint Kinase 1 (CHK1) mediated phosphorylation of SYK(*L*) and associated SYK degradation has been considered an oncogenic process [105], associating low levels of SYK as a factor of poor survival. On the other hand, [103] SYK(*S*) expression promotes metastasis development in HCC and thus leads to poor survival outcome. Furthermore, high SYK expression has been shown to promote liver fibrosis [106]. The development of HCC is closely related to formation and progression of fibrosis. Fibrosis represents excessive accumulation of extracellular matrix (ECM) and scarring tissue in an organ. A fibrotic environment promotes development of dysplastic nodules which can gradually progress to liver tumors [107]. In short, a somewhat inconsistent role of SYK as a tumor supressor or oncogene can be observed in many cancers [101], including liver cancer.

By employing DeRegNet, we identified by means of the approach defined as algorithm 2 a subgroup of HCC patients from the TCGA-LIHC cohort which show poor survival and a distinguished SYK-signaling pattern shown in Fig. 10. The depicted network is manually extracted from the union graph of all the patient’s subgraphs which contained SYK. The network shows SRC-SYK-mediated activation of PI3K-Akt signaling via B-lymphocyte antigen CD19 (CD19) and Phosphatidyl-inositol 4,5-bisphosphate 3-kinase catalytic subunit delta (PI3KCD) (p110$\delta$) [108]. Furthermore, SYK also feeds into mitogen-activated protein kinase 11-13 (p38) signaling (only MAPK13 shown) through GTPase Hras (HRAS) and aspase recruitment domain-containing protein 9 (CASP9). p38 signaling promotes cytokine expression via Growth-regulated alphaprotein (CXCL1). Increased cytokine expression and activation is another canonical effect of SYK signaling [102]. This in turn, activates JAK signaling through Januskinase 3 (JAK3) activity, thereby reinforcing PI3K activation. Interestingly, SYK signaling is consistently linked to the upregulation of the guanine nucleotide exchange factors VAV*1* and VAV*3* [100, 102] [Guanine nucleotide exchange factor (VAV)]. The proto-oncogene VAV*3* is associated to adverse outcomes in colorectal [109] and breast cancer [110, 111]. Furthermore, VAV*3* mutations have been profiled to be potential drivers for liver cancer [112]. VAV signaling is mediated by forming a complex with Lymphocyte cytosolic protein 2 (LCP2) (SLP-76) upon activation of SYK signaling. VAV-meditated Ras-related C3 botulinum toxin substrate 2 (RAC2) activation may play a role in intravastation and motility [113]. Additionally, the subgraph shows upregulation of the B-cell lymphoma 2 (BCL2) gene, a known regulator of apoptosis [114], and vascular endothelial growth factor-C (VEGGC) which can promote metastasis [115] and angiogenesis [116, 117].

### Multi-omics subgraphs with consistent methylation and transcription patterns

**Fig. 11:**
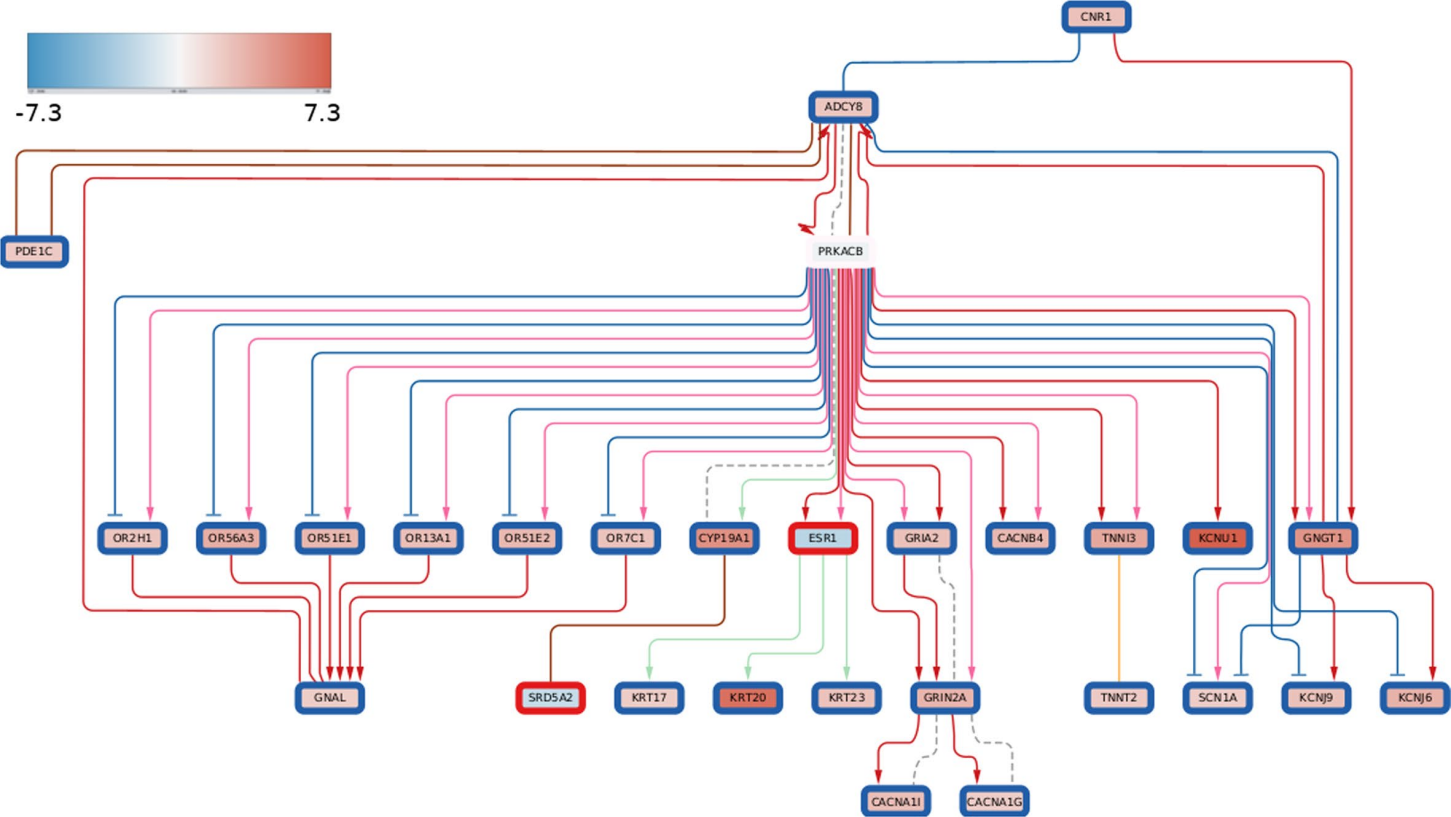
Multi-omics deregulated subgraph showing consistent methylation-transcription patterns. Inner node color represents RNA-Seq log2 fold change while the rim color of a node represents the methylation score $m_v$ where *red* corresponds to upregulated methylation and *blue* corresponds to downregulated methylation. The color of edges indicates the following interactions: activation (red), compound (brown), binding/association (yellow), indirect effect (dashed grey) and expression (green)

To demonstrate a multi-omics application of DeRegNet (i.e. simultaneously using different omics layers) we have utilized RNA-seq and methylation data of the TCGA-LIHC cohort. With the transcriptome-methylome node scores defined in *Materials and Methods* we inferred a deregulated subgraph showing consistent patterns of methylation and transcription. In mammals, hyporegulation of the gene promoter typically leads to downregulation of gene expression and hypermethylation to upregulation of gene expression and hence the optimal subgraph we found represents a functional module which shows consistent patterns of gene regulation by means of promoter methylation [69]. The optimal subgraph depicted in Fig. 11 is centered around protein kinase cAMP-activated catalytic subunit beta (PRKACB) gene. This gene is a catalytic subunit of cAMP (cyclic AMP)-dependent protein kinase. As such, it regulates signalling though cAMP. cAMP signaling is crucial to a large number of processes involved in carcinogenesis, including cell proliferation and differentiation [118]. As visible from the subgraph PRKACB gene is connected to a large number of downstream proteins, that could be potentially regulated through promoter methylation.

## Conclusion

We have shown DeRegNet’s capability to infer relevant patterns to a high degree of accuracy based on simulation benchmarks and showed that it compares favorably to related algorithms. Furthermore, application of DeRegNet to publically available data in a global fashion identified driving factors of liver cancer such as a transcriptionally activated WNT-pathway, thus showing that DeRegNet can provide valuable insight into a given omics experiment and may lead to novel and so far uncharacterized discoveries of genes/pathways involved in carcinogenesis and other biological contexts. An example of such discovery are the outlined insights into the global interaction of integrin and WNT signaling, as well as drug metabolism in liver cancer. In fact, profiling of such interaction between pathways is one of the main strengths of our algorithm over classical gene enrichment methods. Additionally, the application of our subgraph algorithm in a patient-specific manner could identify a consistent subgroup of patients showing poor prognosis potentially due to aberrant SYK signaling and therefore can generate meaningful hypotheses suitable for further experimental follow-up. Given that the SYK example is just one example case of a network-defined cancer gene, this indicates that DeRegNet is a useful hypothesis generation tool for network-guided personalized cancer research. In addition, further modes of application of the DeRegNet algorithm increase the spectrum of meaningful exploratory directions. Note, for example, that we only presented and discussed network-defined cancer genes (i.e. SYK in our subgraph example) for upregulated subgraphs, while we have not presented the results of an analysis based on downregulated or generically deregulated (either up- or downregulated) subgraphs which would lead to similar opportunities. Furthermore, we demonstrated DeRegNet’s explicit multi-omics capability by inferring consistent methylome-transcriptome subgraphs for the TCGA-LIHC dataset. Another venue of further research is the utilization of deregulated subnetworks as features for phenotype prediction tasks. See Additional file 1: Supplementary Material and Methods for some computational experiments regarding survival predictions within the TCGA-LIHC cohort based on subnetwork features. Furthermore, DeRegNet promises to be usable in single cell data analysis. One example of such application can be a construction of cell-type specific subgraphs. For example, genes up- or downregulated in one cell type in comparison to other cell types can be used to define suitable node scores leading to identification of the most active subnetwork in a given cell type relative to other cell types. In conclusion, together with a solid underlying statistical model for which DeRegNet is shown to infer Maximum Likelihood estimates and its open-source implementation, this makes DeRegNet a viable option for any researcher interested in network interactions in an high-throughput omics context.

### Availability of data and materials

- Project name: DeRegNet
- Project home page: https://github.com/KohlbacherLab/deregnet
- Operating system: Linux, OSX and Windows via Docker
- Programming language: C++, Python
- Other requirements: Lemon Graph Library 1.3.1, Gurobi $\ge$ 8
- License: BSD-3-Clause
- Any restrictions to use by non-academics: Gurobi license required

Our implementation is written in C++ and Python and utilizes the Gurobi optmization libary (http://www.gurobi.com/index) and the Lemon graph library (https://lemon.cs.elte.hu/trac/lemon). Our software is open source under a BSD-3-Clause OSI-approved license and is available at https://github.com/KohlbacherLab/deregnet where you can also find installation instructions and usage examples. The algorithm can be run either by using a Python package or a command line tool via Docker images. The Docker images *sebwink/deregnet* are available at Docker Hub (https://hub.docker.com/r/sebwink/deregnet) and bundle all necessary dependencies. Additionaly Docker images are also provided via https://github.com/orgs/KohlbacherLab/packages?repo_name=deregnet. Furthermore, in order to run DeRegNet, a license for the Gurobi optimization library is required. For academic purposes these licences are readily obtained at https://www.gurobi.com/downloads/. The applications of DeRegNet to TCGA data appearing in this paper can be found at https://github.com/KohlbacherLab/deregnet-tcga. DeRegNet depends on a C++ library called *libgrbfrc* (https://github.com/KohlbacherLab/libgrbfrc) to solve fractional integer programs with Gurobi which was implemented by the authors of DeRegNet which is also available under the BSD-3-Clause open source license. Finally, to run the synthetic benchmarks presented in this paper, one can follow the instructions at https://github.com/KohlbacherLab/deregnet/tree/master/examples/custom-python-script. The benchmark code and results as obtained by the authors and presented in Fig. 4 are available here: https://github.com/KohlbacherLab/deregnet/tree/0.99.999/benchmark.

For more information, see Additional file 1: Supplementary Material and Methods.

## Supplementary information

**Additional file 1: Supplementary Material and Methods.** Provides additional details and formalized exposition of many aspects of DeRegNet. In particular, it provides details on directions on how to run the DeRegNet software, definition and derivation of the probabilistic model underlying DeRegNet, as well as the proof that DeRegNet corresponds to maximum likelihood estimation under outlined model, DeRegNet in the context of the general optimization problem referred to as the *Maximum Average Weight Connected Subgraph Problem* and its relatives, proofs of certain structural properties of DeRegNet solutions, different application modes of the DeRegNet algorithms, fractional mixed-integer programming as it relates to the solution of DeRegNet instances, lazy constraints in branch-and-cut MILP solvers as it relates to DeRegNet, further solution technology employed for solving DeRegNet instances, DeRegNet benchmark simulations and use of DeRegNet subgraphs as a basis for feature engineering for survival prediction on the TCGA-LIHC dataset.

**Additional file 2: Supplementary Figures.** This document contains supplementary figures associated to the main text.

## Abbreviations

AP-1: Activator protein 1
BCL2: B-cell lymphoma 2
BIRC5: Baculoviral IAP repeat containing 5
CASP9: Aspase recruitment domain-containing protein 9
CD19: B-lymphocyte antigen CD 19
CDC25C: Cell division cycle 25 homolog C/M-phase inducer phosphatase 1
CDK1: Cyclin-dependent kinase 1
CHK1: Checkpoint kinase 1
CTNNB1: $$\beta$$-Catenin 18
CXCL1: Growth-regulated alphaprotein
CYP: Cytochrome P450
FOS: AP-1 transcription factor subunit/Fos proto-oncogene
FZD10: Frizzled 10
GPC3: Glypican-3
HRAS: GTPase Hras
JAK3: Januskinase 3
JUN AP-1: Transcription factor subunit/Jun proto-oncogene
LCP2: Lymphocyte cytosolic protein 2
LEF1: Lymphoid enhancer-binding factor 1
MAPK: Mitogen activated kinase-like protein
PI3KCD: Phosphatidylinositol 4,5-bisphosphate 3-kinase catalytic subunit delta
PI3K: Phosphatidylinositol-4,5-bisphosphate 3-kinase
PLK1: Polo-like kinase 1
PTK2: Protein tyrosine kinase
RAC2: Ras-related C3 botulinum toxin substrate 2
SOX2: SRY-box 2
SPP1: Secreted phosphoprotein 1
SRC: SRC proto-oncogene/Non-receptor tyrosine kinase
SRY: Sex-determining region Y
SYK: Spleen tyrosine kinase
TERT: Telomere reverse transcriptase
VAV: Guanine nucleotide exchange factor
VEGGC: Vascular endothelial growth factor-C
WNT3A: Wnt Family member 3a
WNT: Wingless-related integration site
DMP: Differentially methylated probe
ECM: Extracellular matrix
GSE: Gene set enrichment
HCC: Hepatocellular carcinoma
KEGG: Kyoto encyclopedia of genes and genomes
LIHC: Liver hepatocellular carcinoma
RNA: Ribonucleic acid
SQN: Subset quantile normalization
TCGA: The Cancer Genome Atlas
w.r.t: with respect to
