# Supplementary material for: De novo identification of maximally deregulated subnetworks based on multi-omics data with DeRegNet
Source: BMC Bioinformatics. 2022 Apr 19;23:139. doi: 10.1186/s12859-022-04670-6 (PMC9020058; doi:10.1186/s12859-022-04670-6)
Supplement: Supplementary file 1 — Additional file 1: Supplementary Material and Methods. Provides additional details and formalized exposition of many aspects of DeRegNet. In particular, it provides details on directions on how to run the DeRegNet software, definition and derivation of the probabilistic model underlying DeRegNet, as well as the proof that DeRegNet corresponds to maximum likelihood estimation under outlined model, DeRegNet in the context of the general optimization problem referred to as the Maximum Average Weight Connected Subgraph Problem and its relatives, proofs of certain structural properties of DeRegNet solutions, different application modes of the DeRegNet algorithms, fractional mixed-integer programming as it relates to the solution of DeRegNet instances, lazy constraints in branch-and-cut MILP solvers as it relates to DeRegNet, further solution technology employed for solving DeRegNet instances, DeRegNet benchmark simulations and use of DeRegNet subgraphs as a basis for feature engineering for survival prediction on the TCGA-LIHC dataset. [file 12859_2022_4670_MOESM1_ESM.pdf]

RESEARCH

Supplementary Material and Methods

Sebastian Winkler[1,2*], Ivana Winkler[2,3,4], Mirjam Figaschewski[1], Thorsten Tiede[1], Alfred Nordheim[3,5] and Oliver Kohlbacher[1,6,7]

[*]Correspondence: sebwink@pm.me
[1]Applied Bioinformatics, Dept. of Computer Science, University of Tuebingen, Tuebingen, Germany
[2]International Max Planck Research School (IMPRS) "From Molecules to Organisms", Tuebingen, Germany
Full list of author information is available at the end of the article

Abstract

This document contains details concerning the Material and Methods outlined in the main paper **de novo identification of maximally deregulated subnetworks based on multi-omics data with DeRegNet**. It provides details about the following topics:

- Directions on how to run the DeRegNet software
- Definition and derivation of the probabilistic model underlying DeRegNet, as well as the proof that DeRegNet corresponds to maximum likelihood estimation under outlined model
- DeRegNet in the context of the general optimization problem referred to as the *Maximum Average Weight Connected Subgraph Problem* and its relatives
- Proofs of certain structural properties of DeRegNet solutions
- Different application modes of the DeRegNet algorithms
- Fractional mixed-integer programming as it relates to the solution of DeRegNet instances
- Lazy constraints in branch-and-cut MILP solvers as it relates to DeRegNet
- Further solution technology employed for solving DeRegNet instances
- DeRegNet benchmark simulations
- Use of DeRegNet subgraphs as a basis for feature engineering for survival prediction on the TCGA-LIHC dataset

Keywords: Biomolecular Networks; Fractional Integer Programming; De-novo subnetwork enrichment; Functional enrichment; Omics data

How to use the DeRegNet software

DeRegNet Docker images and Gurobi setup

The main source code repository for DeRegNet is available here: https://github.com/sebwink/deregnet. DeRegNet is licensed under the BSD 3-clause OSI-approved open source license. The primary route to run DeRegNet is via Docker images which package DeRegNet and all its dependencies. Hence, in terms running DeRegNet on a Linux host there are only two dependencies: Docker and a Gurobi license. The official Docker images for DeRegNet can be found here: https://hub.docker.com/repository/docker/sebwink/deregnet. For instructions for setting up a Gurobi license for running DeRegNet it is referred to the source code repository where one can always find up-to-date information. The *sebwink/deregnet* Docker images support basically two modes of usage: command-line and Python package.

Running DeRegNet via Docker

Assuming you have Docker and a named-user Gurobi license configured, running DeRegNet in command-line mode is as easy as running

```
git clone https://github.com/sebwink/deregnet && cd deregnet
docker/named-user/run sebwink/deregnet:0.99.999 avgdrgnt.py --help
```

which would display all available command-line options for avgdrgnt.py (i.e. DeReg-Net's main script):

```
usage: avgdrgnt.py [-h] [--include-file INCLUDE_FILE]
                   [--include-genesets INCLUDE_GENESETS]
                   [--include INCLUDE]
                   [--include-id-type INCLUDE_ID_TYPE]
                   [--exclude-file EXCLUDE_FILE]
                   [--exclude-genesets EXCLUDE_GENESETS]
                   [--exclude EXCLUDE]
                   [--exclude-id-type EXCLUDE_ID_TYPE]
                   [--debug]
                   [--absolute-values]
                   [--default-score DEFAULT_SCORE]
                   [--score-column SCORE_COL]
                   [--score-file-without-header]
                   [--id-column ID_COL]
                   [--sep SEP]
                   [--biomap-mapper ID_MAPPER]
                   [--score-id-type SCORE_ID_TYPE]
                   [--graph-id-type GRAPH_ID_TYPE]
                   [--graph-id-attr GRAPH_ID_ATTR]
                   [--suboptimal SUBOPTIMAL]
                   [--max-overlap-percentage MAX_OVERLAP]
                   [--gap-cut GAP_CUT]
                   [--time-limit TIME_LIMIT]
                   [--model_sense {min,max}]
                   [--output-path OUTPUT]
                   [--flip-orientation]
                   [--min-size MIN_SIZE] [--max-size MAX_SIZE]
                   [--min-num-terminals MIN_NUM_TERMINALS]
                   [--algorithm {GeneralizedCharnesCooper,Dinkelbach}]
                   [--receptor-file RECEPTOR_FILE]
                   [--receptor-genesets RECEPTOR_GENESETS]
                   [--receptor RECEPTOR]
                   [--receptor-id-type RECEPTOR_ID_TYPE]
                   [--terminal-file TERMINAL_FILE]
                   [--terminal-genesets TERMINAL_GENESETS]
                   [--terminal TERMINAL]
                   [--terminal-id-type TERMINAL_ID_TYPE]
                   --graph GRAPH --scores SCORE_FILE

optional arguments:
  -h, --help            show this help message and exit
  --include-file INCLUDE_FILE
                        Path to GMT or GRP file containing genes defining
                        the include layer.
  --include-genesets INCLUDE_GENESETS
                        Comma seperated list of geneset names for include
                        layer, only applicable if GMT file provided.
  --include INCLUDE     Comma seperated list of IDs defining the include
                        layer.
  --include-id-type INCLUDE_ID_TYPE
                        Id-type for include layer genesets. Options: all
                        supported by chosen biomap mapper
```

```
--exclude-file EXCLUDE_FILE
                      Path to GMT or GRP file containing genes defining
                      the exclude layer.
--exclude-genesets EXCLUDE_GENESETS
                      Comma seperated list of geneset names for exclude
                      layer,only applicable if GMT file provided.
--exclude EXCLUDE     Comma seperated list of IDs defining the exclude
                      layer.
--exclude-id-type EXCLUDE_ID_TYPE
                      Id-type for exclude layer genesets. Options: all
                      supported by chosen biomap mapper
--debug               Debug underlying C++ code with gdb.
--absolute-values     Whether to take absolute values of the scores.
--graph GRAPH         A graphml file containing the graph
                      you want to run DeRegNet with.
--scores SCORE_FILE   A text file containing the scores.
                      See further options below.
--default-score DEFAULT_SCORE
                      The score of nodes in the graph
                      which are not scored in your score file.
                      Default: 0.0
--score-column SCORE_COL
                      Column name of (gene) id in your score file.
                      Default: score
--score-file-without-header
                      Flag to indicate whether the score file has a
                      header or not.
--id-column ID_COL    Column name of (gene) id in your score file.
                      Default: id
--sep SEP             The column seperator in your score file.Options:
                      comma, tab. Default: \t
--biomap-mapper ID_MAPPER
                      biomap mapper you want to use for id mapping.
                      Default: hgnc
--score-id-type SCORE_ID_TYPE
                      Which id type do you have in your score file?
                      Options: all those supported by the biomap mapper
                      you chose or unspecified.
                      Default: same as graph id type
--graph-id-type GRAPH_ID_TYPE
                      Which id type does the graph have?
                      Options: all those supported by the biomap mapper
                      you chose or unspecified.
                      Default: unspecifed i.e. None
--graph-id-attr GRAPH_ID_ATTR
                      Node attribute which contains the relevant
                      id in the graphml. Default: name
--suboptimal SUBOPTIMAL
                      Number of suboptimal subgraphs you want to find.
                      (Increases runtime)
--max-overlap-percentage MAX_OVERLAP
                      How much can suboptimal subgraphs overlap with
                      already found subgraphs. Default: 0
--gap-cut GAP_CUT     Stop optimization prematurely if current solution
                      within GAP of optimal solution. Default: None
--time-limit TIME_LIMIT
                      Set a time limit in seconds. Default: None
--model_sense {min,max}
                      Model sense. Default: max
```

```
--output-path OUTPUT  Folder to which output is written.
                      (Does not have to exist) Default : cwd
--flip-orientation    Set --flip-orientation when you want to flip the
                      orientation of the underlying graph.
--min-size MIN_SIZE   Minimal size of the resulting subgraph(s).
                      Default : 15
--max-size MAX_SIZE   Maximal size of the resulting subgraph(s).
                      Default : 15
--min-num-terminals MIN_NUM_TERMINALS
                      Minimum number of terminals in the resulting
                      subgraph(s). Default : 0
--algorithm {GeneralizedCharnesCooper,Dinkelbach}
                      Algorithm to use to solve the fractional integer
                      programming problem.
                      Default: GeneralizedCharnesCooper.
--receptor-file RECEPTOR_FILE
                      Path to GMT or GRP file containing genes defining
                      the receptor layer.
--receptor-genesets RECEPTOR_GENESETS
                      Comma seperated list of geneset names for receptor
                      layer,only applicable if GMT file provided.
--receptor RECEPTOR   Comma seperated list of IDs defining the receptor
                      layer.
--receptor-id-type RECEPTOR_ID_TYPE
                      Id-type for receptor layer genesets. Options: all
                      supported by chosen biomap mapper
--terminal-file TERMINAL_FILE
                      Path to GMT or GRP file containing genes defining
                      the terminal layer.
--terminal-genesets TERMINAL_GENESETS
                      Comma seperated list of geneset names for terminal
                      layer,only applicable if GMT file provided.
--terminal TERMINAL   Comma seperated list of IDs defining the terminal
                      layer.
--terminal-id-type TERMINAL_ID_TYPE
                      Id-type for terminal layer genesets. Options: all
                      supported by chosen biomap mapper
```

Still in the top-level of the repository, you can find your first subgraph like so:

```
docker/named-user/run sebwink/deregnet:latest avgdrgnt.py \
  --graph test/kegg_hsa.graphml \
  --scores test/data/score.csv \
  --sep , \
  --graph-id-attr ensembl
```

This will generate *deregnet.log* and finally *optimal.graphml* where the former is a log of the optimization procedure carried out by DeRegNet and the latter the resulting optimal subgraph in GraphML format. For more information on GraphML, the most prominent graph serialization format supported by DeRegNet, see: http://graphml.graphdrawing.org/.

DeRegNet Python package via Docker

For more custom analyses it is often necessary to work with the deregnet Python package directly. This is also supported by the *sebwink/deregnet* Docker images

which come with all the relevant packages pre-installed and properly configured. E.g. in order to run the benchmarks presented in the main text, you can follow the directions given here: https://github.com/sebwink/deregnet/tree/master/examples/custom-python-script. Running any Python script which uses the *deregnet* Python package is then as easy as:

```
docker/named-user/run sebwink/deregnet:0.99.999 python3 any_script.py
```

Results concerning the probabilistic model for DeRegNet

This subsection formalizes the notion that a *deregulated* subgraph satisfying given topological constraints should have higher/maximal probability of deregulation with respect to all possible subgraphs of that particular topological class. We present a basic probabilistic model yielding one possible formal probabilistic rationale for optimizing a model of form given in the main paper. Furthermore we provide a suitable interpretation of the model proposed in [1] in terms of that model, showing that DeRegNet solves a more general problem in the statistical sense necessitated by the probabilistic model introduced in the main text.

For sake of locality of exposition we restate the statistical model as introduced in the main text. The model assumes binary node scores $s : V \to \{0,1\}$ which are realizations of random variables $\mathbf{S} = (S_v)_{v \in V}$. Further it is assumed the existence of a subset of vertices $V' \subset V$ such that $S_v | v \in V' \sim Ber(p')$ and $S_v | v \in V \backslash V' \sim Ber(p)$ with $p, p' \in (0,1)$ denoting probabilites of deregulation outside and inside of the deregulated subgraph respectively. It is assumed that $p' > p$ to reflect the idea of *higher* deregulation (probability) in the *deregulated* subgraph. The network context (dependency) is introduced via the restriction that $V' \in \mathcal{C}(V) \subset \mathcal{P}(V)$. Here, $\mathcal{C}(V)$ denotes the set of feasible substructures and should (can) reflect topologies inspired by known biomolecular pathway topologies like the one described in [1] and the last subsection. Furthermore it is assumed, that the (S_v), given a network context and deregulation probabilities p, p', are independent. We further introduce the notation $\alpha(\tilde{V}) := |\{v \in \tilde{V} : S_v = 1\}|$ and considering V', p, p' to be parameters, and a subgraph determined by indicator variables x as outlined in the previous subsection, we can state:

Proposition 1

The log-likelihood $\mathcal{L}_s(\tilde{V}, p, p') = \log \mathbf{P}(\mathbf{S} = \mathbf{s} | V' = \tilde{V}, p, p')$ *under above model is given by:*

$$s^T x \log \frac{p'(1-p)}{p(1-p')} - e^T x \log \frac{1-p}{1-p'} + s^T e \log p + (e-s)^T e \log(1-p).$$

Proof

$$\begin{aligned}\mathbf{P}(\mathbf{S} = \mathbf{s} | V' = \tilde{V}, p, p') &= \prod_{v \in \tilde{V}} \mathbf{P}(S_v = s_v | V' = \tilde{V}, p') \cdot \prod_{v \in V \backslash \tilde{V}} \mathbf{P}(S_v = s_v | V' = \tilde{V}, p) \\ &= p'^{\alpha(\tilde{V})} (1-p')^{|\tilde{V}| - \alpha(\tilde{V})} p^{\alpha(V \backslash \tilde{V})} (1-p)^{|V \backslash \tilde{V}| - \alpha(V \backslash \tilde{V})}\end{aligned}$$

Employing decision variables $x_v = \mathbf{I}(v \in \tilde{V})$, we can write $\alpha(\tilde{V}) = s^T x, |\tilde{V}| = e^T x, \alpha(V \backslash \tilde{V}) = s^T(e-x)$ and $|V \backslash \tilde{V}| = e^T(e-x)$. It follows that the log-likelihood $\mathcal{L}_s(x,p,p') = \mathcal{L}_s(\tilde{V},p,p') = \log \mathbf{P}(\mathbf{S}=\mathbf{s}|V'=\tilde{V},p,p')$ can be written as:

$$\begin{aligned}\mathcal{L}_s(\tilde{V},p,p') &= s^T x \log p' + (e-s)^T x \log(1-p') \\ &\quad + s^T(e-x)\log p + (e-s)^T(e-x)\log(1-p) \\ &= s^T x \log \frac{p'(1-p)}{(1-p')p} - e^T x \log \frac{1-p}{1-p'} + s^T e \log p + (e-s)^T e \log(1-p)\end{aligned}$$

■

We call an optimization model maximizing the objective $s^T x$ subject to any constraints on x (the subgraph topology) a *model of Backes-type* [1]. Note that the DeRegNet model reduces to a Backes-type model in case of $k_{min} = k_{max}$.

Proposition 2

Any subgraph model of Backes-type enforcing a fixed subgraph size can be interpreted as maximum likelihood estimation with respect to subgraph structure given the above model.

Proof Given the log-likelihood as determined by proposition 1, ignoring the constant term with respect to x, a maximum likelihood estimator V^* with respect to subgraph structure can be determined as follows:

$$V^* \in \underset{\tilde{V} \subset \mathcal{C}(V)}{\operatorname{argmax}} \mathcal{L}_s(\tilde{V},p,p') \quad (1)$$

$$= \underset{\tilde{V} \subset \mathcal{C}(V)}{\operatorname{argmax}} \left\{ s^T x \log \frac{p'(1-p)}{p(1-p')} - e^T x \log \frac{1-p}{1-p'} \right\} \quad (2)$$

$$= \underset{\tilde{V} \subset \mathcal{C}(V)}{\operatorname{argmax}} \left\{ s^T x \log \frac{p'(1-p)}{p(1-p')} \right\} \quad (3)$$

$$= \underset{\tilde{V} \subset \mathcal{C}(V)}{\operatorname{argmax}} s^T x \quad (4)$$

Here, equality (2.4) follows from the assumption that the topological constraints of the optimization model enforce a constant subgraphs size (i.e. $e^T x = k$ for some fixed $k \in \mathbb{N}$). The last equality follows (by assumption $p' > p$) because $\log \frac{p'(1-p)}{p(1-p')} > 0$. Overall, a maximum likelihood estimator is given by a solution to a given Backes-type optimization model max $s^T x$ with subgraph topology restricted to subgraphs from $\mathcal{C}(V)$.

■

In particular, the specific model proposed by [1] lends itself to the just justified interpretation:

Corollary 1

The optimization model suggested by [1] can be interpreted as maximum likelihood estimation with respect to subgraph structure given the above probabilistic model.

We now proceed to provide a maximum likelihood interpretation for the DeRegNet model. Since the DeRegNet model does not assume a fixed subgraph size, above conclusions do not apply. Under the assumption that the parameter p is estimated external to the model and represents some general base level of deregulation one can by (conceptual) reduction from the full log-likelihood $\mathcal{L}_s(\tilde{V}, p, p')$ to $\mathcal{L}_s(\tilde{V}, p')$ state the following proposition.

Proposition 3

Solving a DeRegNet instance amounts to maximum likelihood estimation under above model with respect to subgraph structure and deregulation probability p' (assuming $p' > 0$).

Proof Given the log-likelihood as in proposition 1, one can differentiate with respect to p':

$$\frac{\partial}{\partial p'} \mathcal{L}_s(\tilde{V}, p, p') = \frac{\partial}{\partial p'} \mathcal{L}_s(\tilde{V}, p') \tag{5}$$

$$= \frac{\partial}{\partial p'} s^T x \log \frac{p'(1-p)}{(1-p')p} - \frac{\partial}{\partial p'} e^T x \log \frac{1-p}{1-p'} \tag{6}$$

By computing

$$\frac{\partial}{\partial p'} \log \frac{p'(1-p)}{(1-p')p} = \frac{\partial}{\partial p'} \log \frac{p'}{p} - \frac{\partial}{\partial p'} \log \frac{1-p'}{1-p} \tag{7}$$

$$= \frac{p}{p'} \cdot \frac{1}{p} - \frac{1-p}{1-p'} \cdot \frac{-1}{1-p} \tag{8}$$

$$= \frac{1}{p'} + \frac{1}{1-p'} \tag{9}$$

and

$$\frac{\partial}{\partial p'} \log \frac{1-p'}{1-p} = -\frac{1}{1-p'} \tag{10}$$

one obtains

$$\frac{\partial}{\partial p'} \mathcal{L}_s(\tilde{V}, p') = s^T x \frac{1}{p'} + s^T x \frac{1}{1-p'} - e^T x \frac{1}{1-p'} \tag{11}$$

Requiring $\frac{\partial}{\partial p'} \mathcal{L}_s(\tilde{V}, p'^*) = 0$ and with

$$\frac{\partial}{\partial p'} \mathcal{L}_s(\tilde{V}, p'^*) = 0 \Leftrightarrow \frac{1-p'^*}{p'^*} + 1 = \frac{e^T x}{s^T x} \tag{12}$$

$$\Leftrightarrow p'^* = \frac{s^T x}{e^T x} \tag{13}$$

and since $s^T x \leq e^T x$ and $s^T x > 0$ under the assumption that there is at least one node deregulated in the found subgraph and $p' > 0$:

$$\frac{\partial^2}{\partial p'^2}\,\mathcal{L}_s(\tilde{V},p') = s^T x \frac{-1}{p'^2} + s^T x \frac{1}{(1-p')^2} - e^T x \frac{1}{(1-p')^2} \leq -\frac{s^T x}{p'^2} < 0 \tag{14}$$

one arrives at

$$V^*_{MLE} \in \underset{\tilde{V}\subset\mathcal{C}(V)}{\text{argmax}}\; p'^* = \underset{\tilde{V}\subset\mathcal{C}(V)}{\text{argmax}}\; \frac{s^T x}{e^T x} \tag{15}$$

since no terms involving x were dropped in the derivation for p'^*.

■

The propositions of this subsection show, that, given the introduced statistical model, solving a DeRegNet instance instead of an instance of the optimization model proposed in [1] allows to carry out maximum likelihood estimation without the need to fix the subgraph size in advance. Given the assumptions of the model, these results hold regardless of further topological constraints and only relate to the respective objective functions.

Maximum Average Weight Connected Subgraph Problems

(Rooted) Maximum (Average) Weight Connected Subgraph Problems

In terms of mathematical optimization and up to minor modifications, for example the requirement of the subgraphs to be of a certain predefined size $k \in \mathbb{N}$, [1] solve instances of the so called (Rooted) Maximum Weight Connected Subgraph Problem.

Definition 1 (Maximum Weight Connected Subgraph Problem (MWCSP))
Given a directed graph $G = (V, E)$ and node scores $s : V \to \mathbb{R}$, find a set of nodes $V' \subset V$ whose induced subgraph (V', E') maximizes $e^T_{V'}s$ such that there is a node $r \in V'$ such that there is a directed path from r to every other node $v \in V'$.

By fixing the root node in the MWCSP to a particular node in the underlying graph one arrvies at the so called **Rooted Maximum Weight Connected Subgraph Problem (RMWCSP)**:

Definition 2 (Rooted Maximum Weight Connected Subgraph Problem (RMWCSP))
Given a directed graph $G = (V, E)$, node scores $s : V \to \mathbb{R}$, and a node $r \in V$ called the root node, find a set of nodes $V' \subset V$ with $r \in V'$ whose induced subgraph (V', E') maximizes $e^T_{V'}s$ such that there is a directed path from r to every other node $v \in V'$.

The (R)MWCSP has found applications in network biology [2], [1]. It also attracted general computational and theoretical research in recent years [3], [4], from different integer programming formulations and problem-specific branch-and-cut strategies [5], [6], [7], [8], to more recent research on computational strategies for addressing large-scale instances [9] and problem reduction techniques and heuristics [10], [11].

The Maximum Average Weight Connected Subgraph Problem (MAWCSP)

Analogously to the (R)MWCSP one can define versions which strive to optimize the average score in the subgraph.

Definition 3 (Maximum Average Weight Connected Subgraph Problem (MAWCSP))
Given a directed graph $G = (V, E)$ and node scores $s : V \to \mathbb{R}$, find a set of nodes $V' \subset V$ whose induced subgraph (V', E') maximizes $\frac{e_{V'}^T s}{e^T e_{V'}}$ such that there is a node $r \in V'$ such that there is a directed path from r to every other node $v \in V'$.

Definition 4 (Rooted Maximum Average Weight Connected Subgraph Problem (RMAWCSP))
Given a directed graph $G = (V, E)$, node scores $s : V \to \mathbb{R}$, and a node $r \in V$ called the root node, find a set of nodes $V' \subset V$ with $r \in V'$ whose induced subgraph (V', E') maximizes $\frac{e_{V'}^T s}{e^T e_{V'}}$ such that there is a directed path from r to every other node $v \in V'$.

DeRegNet solves extended versions of the (Rooted) Maximum Average Weight Connected Subgraph Problem.

Some formal properties of DeRegNet solutions

In terms of the notation and exact formulation provided in the main text, we will here formally specify certain topological characteristics of solutions of the above model which were hinted at before. For similar proofs and also alternative formulations for the MWCSP it is referred to [1], [7], [6], [5], [8]. I first formally recapture the defining topological feature of problems of (R)M(A)WCS flavour for DeRegNet.

Proposition 4
A feasible subgraph V^* of a DeRegNet instance has the property that any node in the subgraph can be reached from the root of the subgraph.

Proof Any given node $v \in V^*$ of the subgraph is contained in a strongly connected component. By constraints (2.1e) and (2.1f) this strongly connected component either contains the root node or is reachable from some node $u \in V^*$ in the subgraph which is not in that strongly connected component: Let $S \subset V$ be the vertex set inducing the strongly connected component. If the root is not in S we have $e_S^T(x - y) = |S|$ and hence it need to hold $e_{\delta^-(S)}^T x \geq 1$, otherwise one would have $e_S^T(x - y) - e_{\delta^-(S)}^T x \geq |S|$ in violation of constraints (2.1e) and (2.1f). If the root node is in S, it holds that $e_S^T(x-y) = |S|-1$ and hence constraints (2.1e) and (2.1f) always hold due to $e_{\delta^-(S)}^T x \geq 0$. In the case, that the root node is in v's component, v is reachable from the root node. In the case the component does not contain the root, repeat the argument with u instead of v. Again, the root is in the strongly connected component of u or the component is reachable from some $u' \in V^*$, and so on. Since the subgraph has a finite number of strongly connected components, one ultimately will encounter the component containing the root in the above argument which proves the the existence of a path to any arbitrary $v \in V^*$ from the root node. ∎

The terminals from the terminal set T represent terminals of a subgraph in the following sense.

Proposition 5
A feasible subgraph V^* of a DeRegNet instance has the property that a node $v \in V*$ in the subgraph with $v \notin T$ has to have an outgoing edge into the subgraph, i.e. only terminal nodes are allowed to have no outgoing edges within the subgraph.

Proof Given a non-terminal node $v \notin T$ one has constraint (2.1h): $x_v - e^T_{\delta^+(v)} x \leq 0$, i.e. if $x_v = 1$ it has to hold that $e^T_{\delta^+(v)} x \geq 1$. The latter inequality means that there exists another node $u \in V^*$ such that $(v, u) \in E$, E being the edge set of the underlying graph. ■

Further application modes of DeRegNet

Fixing the root node

Instead of the *root* being determined by the algorithm as outlined in the previous paragraph, one can also specify a given node $r \in V$ as root [1]. In this case, one does not need the y variables anymore and, since the constraint logic can be carried over analogously, we can write the corresponding fractional integer problem as:

$$\max_{x \in \{0,1\}^V} \quad \frac{s^T x}{e^T x} \tag{16a}$$

$$\text{s.t.} \quad x_r = 1 \tag{16b}$$

$$k_{min} \leq e^T x \leq k_{max} \tag{16c}$$

$$x_v - e^T_{\delta^-(v)} x \leq 0 \quad \forall v \in V \setminus \{r\} \tag{16d}$$

$$e^T_S x - e^T_{\delta^-(S)} x \leq |S| - 1 \quad \forall S \subset V \; iscs, \; |S| > 1 \tag{16e}$$

$$x_v - e^T_{\delta^+(v)} x \leq 0 \quad \forall v \in V \setminus T \quad \text{if } T \neq \varnothing \tag{16f}$$

$$e^T_{\mathbf{Inc}} x = |\mathbf{Inc}| \tag{16g}$$

$$e^T_{\mathbf{Ex}} x = 0 \tag{16h}$$

Note, that the above formulation is a special case of the more general formulation of the previous section, namely $R = \{r\}$. It is nonetheless convenient to sometimes refer to the tuple $(G, r, T, \mathbf{Ex}, \mathbf{Inc}, s)$ as a *rooted DeRegNet instance*. All other terminology from the general case carries over without modification.

Reversing the orientation

The default version of the just outlined algorithm will find subnetworks which possess a "root" node from which one can reach any other node in the subnetwork. This can be interpreted as the subnetwork being deregulated downstream of that root. As outlined in the previous sections, this root can either be determined by the algorithm or pre-determined by biological curiosity or insight. By reversing the orientation of the graph one can easily obtain subnetworks where the "root" can be reached from any node in the subnetwork. Such a subgraph can be interpreted as deregulated upstream of the either algorithmically determined or user-defined "root" node. In that case a more intuitive name for the "root" is "terminal" or "destination". Formally this difference in the structure of the output can be achieved by substituting the original graph G with the transposed graph $\tilde{G} = (V, \tilde{E})$, $\tilde{E} = \{(u, v) \in V \times V : (v, u) \in E\}$, and defining the models as before with the roles of receptors and terminals exchanged.

Definition 5

A ***reverse solution*** *of a DeRegNet instance* $I = (G, R, T, \boldsymbol{Ex}, \boldsymbol{Inc}, s)$ *with underlying graph* $G = (V, E)$ *is the (graph) transpose of an optimal subgraph of*

the DeRegNet instance $\tilde{\mathbf{I}} = (\tilde{G}, T, R, \boldsymbol{Ex}, \boldsymbol{Inc}, s)$. *The latter is called the reverse instance of* $\mathbf{I}$. *Here,* $\tilde{G}$ *denotes the transposed graph of* G, *i.e.* $\tilde{G} = (V, \tilde{E})$, $\tilde{E} = \{(u, v) \in V \times V : (v, u) \in E\}$.

After the algorithm found subnetworks with respect to the reversed graph the resulting subnetworks have to be re-reversed to reflect physical reality. Also note, that the reversed instance exchanges the roles of receptors and terminal nodes to keep the intuitive notions associated with these terms in line with the topology of the just defined reverse solutions.

Extracting suboptimal subnetworks

Although the strategy to optimize seems like a sensible heuristic, it is nonetheless just an heuristic. There is no intrinsic need for a biological system at hand to behave consistently with this optimization objective in the sense that it is not granted that the patterns found by the algorithm actually correspond to what is biologically important in the given situation. Vice versa, something (nodes, a particular pattern of nodes) not showing up in any subgraph does not mean that they may not be important in the given context. While this cannot be mediated completely, it is sensible to find at least possible suboptimal patterns along with the optimal one. This can be seen as a step to capture mathematically speaking slightly less optimal but biologically potentially similarily or even more important patterns. I implement this notion by following the appproach found in [2] and adapt it to DeRegNet. Given a specified *maximal overlap* $\alpha \in [0, 1)$ and a (induced) subgraph $V^* \subset V$ one adds to the DeRegNet model as stated in the main part of the paper the suboptimality constraint $e_{V^*}^T x \leq \alpha \cdot e^T x$ and reoptimizes, forcing any corresponding subgraph to be found to maximally have $100 \cdot \alpha$ % node overlap with the the nodes of the previously found subgraph. One can iterate this theme. For example, given a set of subgraphs $V^{(1)}, ..., V^{(k)}$ for some $k \in \mathbb{N}$ one can add the constraints $e_{V^{(j)}}^T x \leq \alpha \cdot e^T x$ for all $j = 1, ..., k$ to the DeRegNet instance to obtain a optimal subgraph of that modified DeRegNet instance which is guaranteed to have node overlap $\leq \alpha$ with any of the $V^{(j)}$. With $V^{(1)} = V^*$ being the original optimal subgraph of a DeRegNet instance one thus obtains a series of suboptimal subgraphs $V^{(2)}, ..., V^{(k)}$. The question which k to choose can be for example decided such that one chooses the k for which $\frac{e_{V^{(k+1)}}^T s}{|V^{(k+1)}|} < \beta \cdot \frac{e_{V^*}^T s}{|V^*|}$ for the first time for some $\beta \in [0, 1]$. Here, β quantifies the degree of suboptimality one is willing to accept.

Fractional mixed-integer programming

Definition 6 (Fractional mixed-integer linear program; FMILP)
A ***Fractional mixed-integer linear program (FMILP)*** *is an optimization problem of the following structure:*

$$\max \quad \frac{c^T x + d}{p^T x + q} \tag{17a}$$

$$\text{s.t.} \quad x \in \mathbb{R}^{n_c} \times \mathbb{Z}^{n_i} \tag{17b}$$

$$Ax \leq b \tag{17c}$$

Here, $c, p \in \mathbb{R}^n$, $d, q \in \mathbb{R}$ define the objective, $A \in \mathbb{R}^{m \times n}$, $b \in \mathbb{R}^m$ define $m \in \mathbb{N}$ linear constraints and $n_c \in \mathbb{N}$, $n_i \in \mathbb{N}$ denote the number of continuous and discrete (integer) variables.

We assume $\forall x \in \mathcal{F} : p^T x + q > 0$, $\mathcal{F} := \{x \in \mathbb{R}^n : Ax \leq b\}$. Fractional mixed-integer linear problems are hence mixed-integer problems except for the objective which is a rational function with linear enumerator and denominator instead. While a FMILP is non-convex, it turns out that a FMILP is pseudolinear and hence quasilinear, rendering local optima to be globally optimal [12].

Proposition 6

A FMILP is pseudoconvex and pseudoconcave.

Proposition 7

A FMILP is strictly quasiconvex and strictly quasiconcave.

Proposition 8

A local optimum of a FMILP is also a global optimum.

The latter facts render FMILP solvable by any generic mixed-integer nonlinear programming (MINLP) solver which can handle pseudolinear objective functions [12]. Empirically, it was shown that iterative schemes [12] or linearization-reformulation approaches [13] outperform generic MINLP solvers with respect to computing time and memory footprint. These approaches rely on a mixed-integer linear programming (MILP) solver as their optimization kernel, hence unlocking the power of modern MILP software, and rely on transforming the original problem into a (sequence of) MILP problem(s). The DeRegNet software package discussed in the main text implements a Dinkelbach-type algorithm [12] and a reformulation-linearization method [13] resembling the Charnes-Cooper method [14] for solving fractional linear programs (FLP). The following sections provide algorithmic details on the these methods.

Dinkelbach-type algorithm (Dinkelbach algorithm)

Originating in the 1960's [15, 16] and studied in the context of FMILP problems [17, 12] later on, the Dinkelbach algorithm relies on the iterative solution of linear problems only containing the original variables and an auxiliary iteration parameter. *Algorithm 1* details the procedure. In the following, as well as in the entire thesis, *Dinkelbach algorithm* and *Dinkelbach-type algorithm* are used synonymously to refer to *Algorithm 1*.

Algorithm 1: Dinkelbach-type algorithm

Input: FMILP with feasible set $\mathcal{S}$

Output: solution x^* of FMILP

Initialization:

$\pi = 0$

$\epsilon > 0$ (termination tolerance)

$F = \infty$

while $F > \epsilon$ **do**

$\quad x^* = arg\,max\,\{c^T x + d - \pi\,(p^T x + q) : x \in \mathcal{S}\}$

$\quad F = c^T x^* + d - r\,(p^T x^* + q)$

$\quad \pi = \dfrac{c^T x^* + d}{p^T x^* + q}$

return x^*

The mixed-integer linear program appearing in the *while*-loop of *algorithm 1* is called a *Dinkelbach iteration problem.* Dinkelbach's algorithm iteratively solves a sequence Dinkelbach iteration problems until some convergence criterion is met. The follwing subsection shows that this procedure indeed solves the original FMILP.

Correctness of Dinkelbach's Algorithm (1) - based on You et al.[12]

In order to facilitate the following exposition the functions $N : \mathcal{F} \to \mathbb{R}, N(x) := c^T x + d$ for the nominator and $D : \mathcal{F} \to \mathbb{R}, D(x) := p^T x + q$ for the denominator of the objective function are introduced. Without loss of generality one can set $d = q = 0$ since one can introduce dummy variables x_d and x_q with linear constraints $x_d = x_q = 1$ and corresponding coefficients $c_d = p_q = 1$ leading to $N(x) = c^T x + c_d x_d$ and $D(x) = p^T x + p_q x_q$. Furthermore, define $L_\pi(x) := N(x) - \pi D(x)$ and $F : \mathbb{R} \to \mathbb{R}, F(\pi) := \max\{L_\pi(x) : x \in \mathcal{F}\}$ be the optimal objective value of a Dinkelbach iteration problem as a function of the auxiliary parameter π. Without loss of generality we assume $D(x) > 0$ for all $x \in \mathcal{F}$.

The two main results concerning Dinkelbach's algorithm are the following:

Proposition 9 (Optimality criterion, [13] Proposition 1)
$F(\pi^*) = \max\{N(x) - \pi D(x) : x \in \mathcal{F}\} = 0 \iff \pi^* = \frac{N(x^*)}{D(x^*)} = \max\{\frac{N(x)}{D(x)} : x \in \mathcal{F}\}$ *where* $x^* = \text{argmax}\{\frac{N(x)}{D(x)} : x \in \mathcal{F}\}$

Proposition 10 (Convergence (rate), [13] Proposition 2)
Dinkelbach's algorithm converges superlinearly to π^* *in where* $x^* \in \text{argmax}\{\frac{N(x)}{D(x)} : x \in \mathcal{F}\}$ *and* $\pi^* = \frac{N(x^*)}{D(x^*)}$.

We follow [13] in proving the above propositions via a series of lemmas.

Lemma 1 ([13] Appendix, Lemma 4)
F is convex.

Proof For $\lambda \in [0, 1]$, let $x_\lambda \in \mathcal{F}$ be $x_\lambda \in \text{argmax}\{L_{\lambda\pi' + (1-\lambda)\pi''}(x) : x \in \mathcal{F}\}$ with $\pi', \pi'' \in \mathbb{R}$. Then:

$$F(\lambda\pi' + (1-\lambda)\pi'') = \max\{L_\pi(x) : x \in \mathcal{F}\} \tag{18}$$
$$= N(x_\lambda) - [\lambda\pi' + (1-\lambda)\pi'']D(x) \tag{19}$$
$$= \lambda[N(x_\lambda) - \pi' D(x_\lambda)] + (1-\lambda)[N(x_\lambda) - \pi'' D(x_\lambda)] \tag{20}$$
$$= \lambda F(\pi') + (1-\lambda)F(\pi'') \tag{21}$$

■

Lemma 2 ([13] Appendix, Lemma 5)
F is strictly monotonically increasing, i.e. $\pi' < \pi'' \implies F(\pi') < F(\pi'')$.

Proof Given $\pi' < \pi''$ one obtains with $x' = \text{argmax}\{L_{\pi'}(x) : x \in \mathcal{F}\}$ and $x'' = \text{argmax}\{L_{\pi''}(x) : x \in \mathcal{F}\}$:

$$F(\pi'') = N(x'') - \pi'' D(x'') \tag{22}$$
$$< N(x'') - \pi' D(x'') \tag{23}$$
$$\leq N(x') - \pi' D(x') \tag{24}$$
$$= F(\pi') \tag{25}$$

■

Lemma 3 ([13] Appendix, Lemma 6)
$F(\pi) = 0$ *has a unique solution.*

Proof Follows from $\lim_{\pi\to\infty} F(\pi) = -\infty$ and $\lim_{\pi\to-\infty} F(\pi) = \infty$ and F being strictly monotonically increasing (Lemma 2). ■

Lemma 4 ([13] Appendix, Lemma 7)
$\forall x' \in \mathcal{F} : F(\frac{N(x')}{D(x')}) \geq 0$

Proof For any $x' \in \mathcal{F}$ one has:

$$F(\frac{N(x')}{D(x')}) = \max\{N(x) - \frac{N(x')}{D(x')} D(x) : x \in \mathcal{F}\} \tag{26}$$
$$\geq N(x') - \frac{N(x')}{D(x')} D(x') \tag{27}$$
$$= 0 \tag{28}$$

■

One can now prove proposition 1:

Proof of proposition 1 We have to show: $F(\pi^*) \iff \pi^* = \frac{N(x^*)}{D(x^*)} = max_{x\in\mathcal{F}} \frac{N(x)}{D(x)}$.

$\Longrightarrow$: Given $F(\pi^*) = max_{x\in\mathbb{F}} N(x) - \pi^* D(x)$ it follows with $x^* := \text{argmax}\{N(x) - \pi^* D(x) : x \in \mathcal{F}\}$ for all $x \in \mathcal{F}$ $0 = N(x^*) - \pi^* D(x^*) \geq N(x) - \pi^* D(x)$. Hence $\frac{N(x)}{D(x)} \leq \pi^* = \frac{N(x^*)}{D(x^*)}$, i.e. $x^* = \text{argmax}\{\frac{N(x)}{D(x)} : x \in \mathcal{F}\}$.

$\Longleftarrow$: With $x^* = \text{argmax}\{\frac{N(x)}{D(x)} : x \in \mathcal{F}\}$ one has $\pi^* = \frac{N(x^*)}{D(x^*)} \geq \frac{D(x)}{N(x)}$. Under our general assumption $D(x) > 0$ for all $x \in \mathcal{F}$ it follows $N(x) - \pi^* D(x) \leq 0 = N(x^*) - \pi^* D(x^*)$ for all $x \in \mathcal{F}$ which shows $x^* = \text{argmax}\{N(x) - \pi^* D(x) : x \in \mathcal{F}\}$. ■

From now onward, let π^* be the unique solution of $F(\pi) = 0$ and let $x^* \in \text{argmax}\{\frac{N(x)}{D(x)} : x \in \mathcal{F}\}$ with $\pi^* = \frac{N(x^*)}{D(x^*)}$.

Lemma 5 ([13] Appendix, Lemma 8)
Let $x' \in \text{argmax}\{N(x) - \pi' D(x)\}$ *and* $x'' \in \text{argmax}\{N(x) - \pi'' D(x) : x \in \mathcal{F}\}$ *with* $\pi' < \pi''$*, then* $D(x') \geq D(x'')$.

Proof Adding the inequalities $N(x') - \pi' D(x') \geq N(x'') - \pi' D(x')$ and $N(x'') - \pi'' D(x'') \geq N(x') - \pi'' D(x')$ leads to $(\pi'' - \pi')D(x') \geq (\pi'' - \pi')D(x'')$, i.e. $D(x') \geq D(x'')$ since $\pi'' \geq \pi'$ by assumption. ■

Lemma 6 ([13] Appendix, Lemma 9)
Let $x' \in \operatorname{argmax}\{N(x) - \pi' D(x)\}$ *and* $x'' \in \operatorname{argmax}\{N(x) - \pi'' D(x) : x \in \mathcal{F}\}$, *then* $f(x'') - f(x') \geq \frac{F(\pi'')}{D(x'')} - \frac{F(\pi')}{D(x')}$

Proof From $F(\pi'') = N(x'') - \pi'' D(x'') \geq N(x') - \pi'' D(x'')$ it follows $\frac{N(x'')}{D(x')} - \pi'' \frac{D(x'')}{D(x')} \geq \frac{N(x')}{D(x')} - \pi''$. This implies:

$$\frac{N(x'')}{D(x'')} - \frac{N(x')}{D(x')} \geq \frac{N(x'')}{D(x'')} + (-\pi'' + \frac{D(x'')}{D(x')}\pi'' - \frac{N(x'')}{D(x')}) \tag{29}$$

$$= \frac{N(x'')}{D(x'')} - \frac{N(x'')}{D(x')} + \pi''(\frac{D(x'')}{D(x')} - \frac{D(x'')}{D(x'')}) \tag{30}$$

$$= N(x'')(\frac{1}{D(x'')} - \frac{1}{D(x')}) + \pi'' D(x'')(\frac{1}{D(x'')} - \frac{1}{D(x'')}) \tag{31}$$

$$= -F(\pi'')(\frac{1}{D(x')} - \frac{1}{D(x'')}) \tag{32}$$

$$= \frac{F(\pi'')}{D(x'')} - \frac{F(\pi'')}{D(x')} \tag{33}$$

■

Lemma 7 ([13] Appendix, Lemma 10)
Let $x' \in \operatorname{argmax}\{N(x) - \pi' D(x)\}$ *and* $x'' \in \operatorname{argmax}\{N(x) - \pi'' D(x) : x \in \mathcal{F}\}$ *and* $F(\pi^*) = 0$, *then if follows for* $\pi' \leq \pi'' \leq \pi^*$, *that* $\frac{N(x')}{D(x')} \leq \frac{N(x'')}{D(x'')}$.

Proof

$$\frac{N(x'')}{D(x'')} - \frac{N(x')}{D(x')} \geq \frac{F(\pi'')}{D(x'')} - \frac{F(\pi')}{D(x')} \tag{34}$$

$$\geq \frac{F(\pi'')}{D(x')} - \frac{F(\pi'')}{D(x')} \tag{35}$$

$$= 0 \tag{36}$$

The first inequality follows from lemma 9, the second from lemma 7 and 8. ■

Lemma 8 ([13] Appendix, Lemma 11)
Let $x' \in \operatorname{argmax}\{N(x) - \pi' D(x)\}$ *and* $x'' \in \operatorname{argmax}\{N(x) - \pi'' D(x) : x \in \mathcal{F}\}$, *then* $f(x'') - f(x') \leq (-F(\pi'') + (\pi' - \pi'')D(x''))(\frac{1}{D(x')} - \frac{1}{D(x'')})$.

Proof From $N(x') - \pi' D(x') \geq N(x'') - \pi'' D(x'')$ it follows $\frac{N(x')}{D(x')} - \pi' \geq \frac{N(x'')}{D(x')} - \pi' \frac{N(x'')}{D(x')}$ by dividing by $D(x') > 0$. It then follows:

$$f(x'') - f(x') = \frac{N(x'')}{D(x'')} - \frac{N(x')}{D(x')} \tag{37}$$
$$\leq \frac{N(x'')}{D(x'')} - \pi' - \frac{N(x'')}{D(x')} + \pi' \frac{D(x'')}{D(x')} \tag{38}$$
$$= \frac{N(x'')}{D(x'')} - \frac{N(x'')}{D(x')} - \pi' (\frac{D(x'')}{D(x'')} - \frac{D(x'')}{D(x')}) \tag{39}$$
$$= (-N(x'') + \pi' D(x''))(\frac{1}{D(x')} - \frac{1}{D(x'')}) \tag{40}$$
$$= (-F(\pi'') + (\pi' - \pi'')D(x''))(\frac{1}{D(x')} - \frac{1}{D(x'')}) \tag{41}$$

■

Lemma 9 ([13] Appendix, Lemma 12)
Let $x' \in \operatorname{argmax}\{N(x) - \pi' D(x)\}$ *and* $x'' \in \operatorname{argmax}\{N(x) - \pi'' D(x) : x \in \mathcal{F}\}$ *with* $F(\pi^*) = N(x^*) - \pi^* D(x^*) = 0$, *then* $\pi^* - f(x') \leq (\pi^* - \pi')(1 - \frac{D(x^*)}{D(x')})$.

Proof

$$\pi^* - f(x') = f(x^*) - f(x') \tag{42}$$
$$\leq (-F(\pi^*) + (\pi' - \pi^*)D(x^*))(\frac{1}{D(x')} - \frac{1}{D(x^*)}) \tag{43}$$
$$= (\pi' - \pi^*)(\frac{D(x^*)}{D(x')} - 1) \tag{44}$$
$$= (\pi^* - \pi')(1 - \frac{D(x^*)}{D(x')}) \tag{45}$$

where the inequality follows from Lemma 11. ■

Proposition 2 can now be demonstrated as follows:

Proof of proposition 2 Let $F(\pi^*) = 0$, i.e. $\pi^* = \max\{\frac{N(x)}{D(x)} : x \in \mathcal{F}\}$. For $i \in \mathbb{N}$, let $\pi_{i+1} = \frac{N(x_i)}{D(x_i)} = f(x_i)$ where $x_i \in \operatorname{argmax}\{N(x) - \pi_i D(x) : x \in \mathcal{F}\}$ it follows with Lemma 9:

$$\frac{\pi^* - \pi_{i+1}}{\pi^* - \pi_i} = \frac{\pi^* - f(x_i)}{\pi^* - \pi_i} \leq 1 - \frac{D(x^*)}{D(x_i)}$$

Since $\pi_i \leq \pi^* = \max\{\frac{N(x)}{D(x)} : x \in \mathcal{F}\}$ it follows with Lemma 5 $\frac{D(x^*)}{D(x_i)} \leq 1$ and since $\frac{D(x^*)}{D(x_i)} > 0$ one obtains

$$0 \leq \frac{\pi^* - \pi_{i+1}}{\pi^* - \pi_i} < 1$$

for all $i \in \mathbb{N}$. The latter inequality demonstrates superlinear convergence. ■

Correctness of Dinkelbach's algorithm for solving the DeRegNet model

Here, we prove that the fractional integer programming model for finding deregulated subgraphs proposed in the main text can be solved via Dinkelbach's algorithm. The only points to clarify are the suitability of Dinkelbach's algorithm for models with lazy constraints, the suitability of an initial value for π of 0 and the positivity of the objective denominator, see last subsection.

Proposition 11 (Dinkelbach-type algorithm for DeRegNet)
The Dinkelbach algorithm is correct for the fractional integer programming problem of DeRegNet.

Proof The first point to observe is that the objective of DeRegNet is always ≥ 0 hence the initialization condition of the iteration parameter $\pi = 0$ statisfies $\pi \leq \pi^*$. Furthermore, for subgraphs which are constrained to contain at least one node, the denominator of the objective is strictly positive. These two properties are enough to guarantuee convergence of Dinkelbach's algorithm as detailed above. Also since the original decision variables are also part of the parameterized Dinkelbach iteration problems introducing lazy constraints is technically feasible. Since lazy constraints can only decrease the maximum objective, after every iteration $\pi \leq \pi^*$ where π^* is the optimal objective determined by the current constraints and hence lazy constraints do not interfere with the correctness of Dinkelbach's algorithm since it requires a starting value of π which is a lower bound of the optimal objective value. ∎

Note that lazy constraints effectively amount to restarting Dinkelbach's algorithm (in a valid initialization state) every time a lazy constraint is added. Hence, convergence can also only be considered superlinear (see last subsection) with respect to the current optimal objective determined by the lazy constraints.

Reformulation-Linearization methods

Generalized Charnes-Cooper method

The so called Generalized Charnes-Cooper transformation [13] described in this subsection derives its name and general idea from the classical Charnes-Cooper transformation [14] used to solve continuous fractional linear problems. Consider the above general form of a FMIP in the following slightly more detailed format:

$$\max \quad \frac{c_c^T x_c + c_i^T x_i + d}{p_c^T x_c + p_i^T x_i + q} \tag{46a}$$

$$\text{s.t.} \quad x = \begin{pmatrix} x_c \\ x_i \end{pmatrix} \in \mathbb{R}^{n_c} \times \mathbb{Z}^{n_i} \tag{46b}$$

$$A_c x_x + A_i x_i \leq b \tag{46c}$$

where we explicitly decomposed the variable x into its continuous and integer parts. Analogously we have $c = \begin{pmatrix} c_c \\ c_i \end{pmatrix}$ with $c_c \in \mathbb{R}^{n_c}$, $c_i \in \mathbb{R}^{n_i}$, and $p = \begin{pmatrix} p_c \\ p_i \end{pmatrix}$ with

$p_c \in \mathbb{R}^{n_c}$, $p_i \in \mathbb{R}^{n_i}$, and $A = \begin{pmatrix} A_c & A_i \end{pmatrix}$ with $A_c \in \mathbb{R}^{m \times n_c}$, $A_i \in \mathbb{R}^{m \times n_i}$. As detailed in [13] one can now define additional variables $u := \dfrac{1}{p_c^T x_c + p_i^T x_i + q}$ and $z := \dfrac{x_c}{p_c^T x_c + p_i^T x_i + q} = ux$. Note, since we assume that there exists some real $m > 0$ such that $p^T x + q > m$ for all feasible $x \in \mathbb{R}^n$, it follows that $u > 0$. After incorporating the definition of u as a further constraint and multiplying all constraints with u one arrives at the following quadratic mixed-integer problem:

$$\max \quad c_c^T z + c_i^T (u \cdot x_i) + d \tag{47a}$$

$$\text{s.t.} \quad x_i \in \mathbb{Z}^{n_i}, \;\; z \in \mathbb{R}^{n_c}, \;\; u \in \mathbb{R}_+ \tag{47b}$$

$$p_c^T z + p_i^T (u \cdot x_i) + qu = 1 \tag{47c}$$

$$A_c z + A_i (u \cdot x_i) - bu \leq 0 \tag{47d}$$

Note that the above problem is not a MILP but a quadratic mixed-integer problem due to the terms ux_i in the transformed constraints. This is addressed in the next subsection. With the notation of this subsection one can formulate the following propositions formalizing the equivalence of the two model formulations [13]:
Proposition 12 (Feasible points of the generalized Charnes-Cooper transform)
A point (x_c, x_i) is a feasible solution of problem (A.30) if and only if (z, x_i, u) is a feasible solution of problem (A.31).

Proof Because of $p_c^T x_c + p_i^T x_i + q > 0$ this is true by definition of u and z. ■

Proposition 13 (Equivalence of solutions of the generalized Charnes-Cooper transform)
An feasible point (x_c^*, x_i^*) of (A.30) is optimal if and only if (z^*, x_i^*, u^*) is optimal for (A.31). It holds that $z^* = u^* x_c^*$ and $u^* = \frac{1}{p_c^T x_c^* + p_i^T x_i^* + q}$.

Proof By definition of u and z the objectives of (A.30) and (A.31) have the same value for all feasible points. The relations for the optimal points are also true by definition. ■

With respect to lazy constraints involving the integer variables x_i there do not arise any complications since they are part of both problem formulations. Lazy constraints for the continuous variables x_c require more care due to the necessity to transform the constraints correspondingly. The DeRegNet model does only contain integer (in fact, binary) variables and hence it is straight-forward to incorporate lazy constraints in the solution process in terms of the original model formulation.

Linearization of binary-continuous quadratic constraints
In contrast to the iterative Dinkelbach scheme, the reformulation-linearization method described in the last section relies on the linearization of products of integer and continuous variables. Since we only deal with binary variables in this paper, we assume from now on that all integer variables are in fact binary. In

case of a proper integer variable $x \in D \subset \mathbb{Z}$, one can introduce auxiliary binary variables $x'_d \in \{0, 1\}, d \in D$ with $x = \sum_{d \in D} d \cdot x'_d$ and $\sum_{d \in D} x'_d = 1$ in order to transform its product with continuous variables into a sum of products between binary and continuous variables. There exist variations on the theme of linearization [18], [19], but here we will present the implemented most basic version going back to [20].

Given a continuous variable $v \in \mathbb{R}$ and a binary variable $x \in \{0, 1\}$ one introduces a third (continuous) variable $z \in \mathbb{R}$ corresponding to $z = vx$ and substitutes any appearance of the product vx with z. Along with z one introduces the following constraints to ensure equivalence:

$$\begin{aligned} &z \leq Ux \\ &z \geq Lx \\ &v - U(1 - x) \leq z \\ &v - L(1 - x) \geq z \end{aligned} \tag{48}$$

Here, $U \in \mathbb{R}$ is an upper and $L \in \mathbb{R}$ is a lower bound of v which are either given by the problem formulation itself, can be inferred from manual insight into the problem or by solving a certain MILP in some cases. See below.

Proposition 14 (Linearization binary-continuous products)
Let $v \in \mathcal{S} \subset \mathbb{R}$ with bounded $\mathcal{S}$ and let $x \in \{0, 1\}$ and $z \in \mathbb{R}$. Furthermore $U \geq \sup \mathcal{S}$ and $L \leq \inf \mathcal{S}$. Then, the constraints (A.15) are statisfied if and only if $z = vx$.

Proof Let $z = vx$, then $z = vx \leq Ux$ since U is an upper bound of v and $z = vx \geq Lx$ since L is a lower bound of v. Also for the case $x = 1$ one has $v - U(1 - x) = v = vx = z$ and $v - L(1 - x) = v = vx = z$ and for the case $x = 0$ the two constraints $v - U(1 - x) \leq z$ and $v - L(1 - x) \geq z$ reduce to $v \leq U$ and $v \geq L$ respectively which is true by assumption. Conversely, let the constraints in (A.15) be satisfied. Then in the case $x = 1$, the constraints $v - U(1 - x) \leq z$ and $v - L(1 - x) \geq z$ imply $v \leq z \leq v$ and hence $z = v = vx$. In the case $x = 0$ the first two constraints of (A.15) imply $z = 0 = vx$. ■

The lower bound L and the upper bound U can generally be obtained by solving suitable MILPs [13] involving the denominator of the original objective. To obtain the (tightest possible) lower bound one can solve the following problem:

$$\max \quad p_c^T x_c + p_i^T x_i + q \tag{49a}$$

$$\text{s.t.} \quad x = \begin{pmatrix} x_c \\ x_i \end{pmatrix} \in \mathbb{R}^{n_c} \times \mathbb{Z}^{n_i} \tag{49b}$$

$$A_c x_x + A_i x_i \leq b \tag{49c}$$

Analogously to obtain the (tightest possible) upper bound one can solve the following minimization problem:

$$\min \quad p_c^T x_c + p_i^T x_i + q \tag{50a}$$

$$\text{s.t.} \quad x = \begin{pmatrix} x_c \\ x_i \end{pmatrix} \in \mathbb{R}^{n_c} \times \mathbb{Z}^{n_i} \tag{50b}$$

$$A_c x_x + A_i x_i \leq b \tag{50c}$$

Note however, that any lower and upper bound would work. The trade-off between less tight bounds on the denominator variable and the necessity of solving up to two MILPs up front has to be decided for every model.

In case of DeRegNet, lower and upper bound on the objective denominator are explicitly set in the problem formulation in the form of minimal and maximal subgraph size. Hence one does not have to solve any MILPs up front and has (optimal) lower and upper bounds for the inverse denominator readily available due to the problem formulation.

Software for solving fractional integer programs: libgrbfrc

In order to solve the fractional integer programs formulated in the main text, a C++ library based on the commercial Gurobi solver was implemented. libgrbfrc (https://sebwink.github.io/libgrbfrc/) in particular implements the two solution methods from above: Dinkelbach's algorithm and the generalized Charnes-Cooper transform. Due to the requirements of the developed optimization models (see main text) the implementations support lazy constraints. Academic licenses for Gurobi are readily obtained.

Lazy constraints in branch-and-cut MILP solvers

For reference this section contains an high-level outline of how lazy constraints fit into branch-and-cut algorithms for solving mixed-integer programs. The exposition is adapted from [21].

Let a MILP with $n_c \in \mathbb{N}$ continuous and $n_i \in \mathbb{N}$ integer variables of the following form be given:

$$\max \quad c^T x + d^T y \tag{51a}$$

$$\text{s.t.} \quad x \in \mathbb{R}^{n_c} \tag{51b}$$

$$y \in \mathbb{Z}^{n_i} \tag{51c}$$

$$Ax + By \leq b \tag{51d}$$

$$x, y \geq 0 \tag{51e}$$

Here $c \in \mathbb{R}^{n_c}$, $d \in \mathbb{R}^{n_i}$, $A \in \mathbb{R}^{m \times n_c}$ and $B \in \mathbb{R}^{m \times n_i}$ for some $m \in \mathbb{N}$. The *(natural) linear programming relaxation* of a MILP of the above form is the following:

$$\max \quad c^T x + d^T y \tag{52a}$$

$$\text{s.t.} \quad x \in \mathbb{R}^{n_c} \tag{52b}$$

$$y \in \mathbb{R}^{n_i} \tag{52c}$$

$$Ax + By \leq b \tag{52d}$$

$$x, y \geq 0 \tag{52e}$$

Lazy constraints are constraints which are not initially explicitly part of the model formulation, the reason usually being that it would require an infeasable exponential number of constraints (with respect to the number of variables).
The classical branch-and-cut strategy for solving MILPs with lazy constraints can then be formulated as the following algorithm 2.

Algorithm 2: Branch-and-cut for MILPs with lazy constraints

Input: MILP and lazy constraints
Output: Solution of MILP satisfying any lazy constraint
Initialization:
$\mathcal{L} = \{\text{MILP}\}$ (Set of MILP problems in search tree)
$\underline{z} = -\infty$ (Current best lower bound for optimal objective)
$(x^*, y^*) = (null, null)$ (Current best feasible solution)
while $\mathcal{L} \neq \varnothing$ **do**
  Choose P from $\mathcal{L}$ and remove P from $\mathcal{L}$
  (*) Solve linear programming relaxation of P
  Let z be the solution value and (x, y) be the solution of the relaxation
  if $z > \underline{z}$ **then**
    if (x, y) *feasible for* P **then**
      Find the set $\mathbf{V}$ of violated lazy constraints
      if $\mathbf{V} = \varnothing$ **then**
        $(x^*, y^*) := (x, y)$
        $\underline{z} := z$
      else
        Insert P back into $\mathcal{L}$
        Add lazy constraints from $\mathbf{V}$ to models in $\mathcal{L}$
    else
      if *you want to add cuts* **then**
        Add cuts and GOTO (*)
      else
        Branch and add created subproblems to $\mathcal{L}$
return (x^*, y^*), $\underline{z}$

Lazy constraints for the DeRegNet model

For DeRegNet the lazy constraint separation subroutine centers around finding the strongly connected components of the given solution. This is generally considered an efficiently solvable problem.

Strongly connected components

Given a directed graph $G = (V, E)$ one says that G is strongly connected if and only if there is a directed path from every node $v \in V$ to every other node $u \in V$. A

strongly connected component of a directed graph is any maximal subgraph which is strongly connected, i.e. adding any node not in the subgraph would render the resulting subgraph to be not strongly connected anymore. Sometimes one refers to $V' \subset V$ as inducing a strongly connected component if the subgraph induced by V' is a strongly connected component. One denotes the set of node sets inducing all strongly connected components of a graph $G = (V, E)$ by $\mathbf{SCC}(G) \subset \mathcal{P}(V)$. The three classical algorithms which can be used to solve the problem of finding a directed graph's strongly connected components in $O(|V| + |E|)$ time are the Kosarju-Sharir algorithm [22], Tarjan's algorithm [23] and variants of the path-based strong component algorithm [24]. A strongly connected *subgraph* (in contrast to *component*) is a subgraph of a graph which is strongly connected.

Lazy constraint separation subroutine of DeRegNet

This subsection and algorithm 3 provide the details on the lazy constraint separation subroutine employed for the solution of the DeRegNet model. The formal details are given as algorithm 3. In short, given a (potential) incumbent solution to a DeRegNet instance not containing all strong-component constraints, the subroutine finds the strongly connected components of the corresponding subgraph and checks whether any such component either contains the root node itself or has at least one incoming edge from within the subgraph but from outside the component. If so, the (potential) incumbent is feasible, hence an actual incumbent solution. Otherwise the violated constraint is added to the model in while the (potential) incumbent is declared infeasible. The general implementation strategy employed is based on the one given by [1] where cycles are detected in order to avoid unconnected subgraphs.

Algorithm 3: Lazy constraint subroutine for DeRegNet. In case a potential incumbent is found all strongly connected components are checked to assess feasibility. In case any strongly connected component does not contain the root node and has no incoming edges from another component, a (lazy) constraint enforcing the requirement is added. $\mathbf{SCC}(G)$ denotes the set of all strongly connected components of a graph G.

Input: DeRegNet instance and $x, y : V \to \{0, 1\}$
Output: *True* if x and y do not violate any lazy constraints, *false* otherwise
$V^* = \{v \in V : x_v = 1\}$ (nodes implied by x)
$G^* = (V^*, E^*)$ the subgraph induced by V^*
$\mathcal{C} = \mathbf{SCC}(G^*)$, $\mathcal{C} \in \mathcal{P}(V^*)$ (Find strongly connected components)
for C *in* $\mathcal{C}$ *with* $|C| > 1$ **do**
  if $e_C^T(x - y) - e_{\delta^-(C)}^T x > |C| - 1$ **then**
    return *false*
return *true*

Further technical aspects of solving DeRegNet models

Primal heuristics for the DeRegNet model

Every feasible solution of a mixed-integer program provides a lower bound on the optimal solution value (for maximization problems). The feasible solution which currently gives the best lower bound on the optimal value during a branch-and-bound procedure is called the *incumbent (solution)*. Branch-and-bound (and hence

branch-and-cut) for mixed-integer programs relies on pruning parts of the search tree of LP relaxation subproblems by assessing whether the optimal solution value of a given LP relaxation is less than the best lower bound provided by the incumbent. Primal heuristics [25] aim at finding and/or improving feasible solutions during a branch-and-bound procedure. While some generic methods for primal heuristics exist [26], [27], [28], [29], [30], they tend to be highly problem-specific [25]. Of special interest in that context are primal heuristics for the MWCSP [11], [6], [7]. In the following I describe start and improvement heuristics useful during the solution of DeRegNet instances.

Start heuristics

A priori there is no feasible solution known at the beginning of a branch-and-bound procedure for solving a mixed-integer program. Heuristics which try to find initial feasible solutions are called *start heuristics*. I outline two start heuristics which can be employed at the beginning of the branch-and-bound search for the solution of the DeRegNet model.

Greedy start heuristic. The first start heuristic is called *greedy start heuristic* and basically starts with the highest scoring node and greedily adds neighbors of already added nodes until the average score of the thus defined subgraph starts decreasing. If the currently selected subgraph is feasible upon termination, one has found a feasible solution. The formal procedure is outlined in algorithm 4. There are a number subtleties attached to this start heuristic. First and foremost the procedure only assures the reachability constraints regarding the root node. Most other constraints may or may not be satisfied at any given time during the procedure, mostly: subgraph size constraints and constraints ensuring the necessity of leaf nodes to be from the subset of terminal nodes. While the subgraph size constraint is relatively easily manageable by stopping the procedure when the maximal subgraph size is reached and by restarting in case the minimal subgraph size can not be achieved in the first place. In the latter case, one can restart the procedure from the best scoring node not already selected during earlier attempts of the greedy start heuristic. The issue of the terminal node constraints is not easily handled and hence the greedy start heuristic is in effect only usable in case $T = \varnothing$. Also instances with $\mathbf{Inc} \neq \varnothing$ cannot be handled by this heuristic.

Receptor-terminal shortest path heuristic. The second start heuristic is more suitable in situations where there is a non-empty terminal set T. In short, it finds the shortest path between a pair of receptor and terminal nodes with high node scores. The **SHORTEST_PATH** subroutine referenced in algorithm 5 can be an implementation of any of the canonical algorithms to find single-source shortest paths with unit edge weights in directed graphs in polynomial time [31], [32], [33]. Subject to $\mathbf{Ex} = \mathbf{Inc} = \varnothing$ all connectivity constraints will be satisfied by construction. If the subgraph size constraints are met is up to chance however. Again, running multiple times with the, say K, highest scoring pairs of receptors and terminals, can help in this situation. Note, that the restriction of $\mathbf{Ex} = \mathbf{Inc} = \varnothing$ could be lifted by formulating the corresponding shortest path problem by canonical means

Algorithm 4: Greedy start heuristic for the DeRegNet model

Input: DeRegNet instance with $T = \textsf{Inc} = \varnothing$
Output: Feasible solution of DeRegNet instance **or** *null*
if $R \neq \varnothing$ **then**
  $V_I = R$
else
  $V_I = V \setminus \textsf{Ex}$
$v^* = \text{argmax}_{v \in V_I} s_v$ (Select feasible root with highest score)
$V^* = \{v^*\}$ (Selected DeRegNet solution)
$N = \delta^+(v^*) \setminus \textsf{Ex}$ (Candidate nodes to be potentially added next)
$A^* = s_{v^*}$ (Current average score of selected subgraph)
CONTINUE = **true**
while *CONTINUE and* $|V^*| < k_{max}$ **do**
  $v^* = \text{argmax}_{v \in N} s_v$ (Highest scoring node in candidate set)
  if $s_{v^*} \geq A^*$ **then**
    $A^* = \frac{|V^*|A^* + s_{v^*}}{|V^*|+1}$ (Update average score of selected subgraph)
    $V^* = V^* \cup \{v^*\}$ (Update current subgraphs)
    $N^* = (\delta^+(v^*) \setminus V^*) \setminus \textsf{Ex}$ (New candidate nodes)
    $N = (N \setminus \{v^*\}) \cup N^*$ (Update candidate nodes)
  else
    CONTINUE = **false**
if V^* *feasible* **then**
  return V^* (Return feasible solution of DeRegNet instance)
else
  return *null* (Return nothing to indicate failure to find feasible solution)

in terms of integer programming problems [34]. This possibility is not explored further however since solving integer programs to get initial feasible solutions to integer program may be a slippery slope. In particular in the case of DeRegNet, where the main problem to solve is formulated in terms of decision variables corresponding to nodes while shortest path integer programming formulations usually introduce decision variables corresponding to the edges of the graph.

Improvement heuristics

In case a feasible solution is found at a particular branch-and-bound node (which may be a new incumbent or not), heuristics which try to improve that given feasible solution are called *improvement heuristics*. Here I describe a simple greedy improvement heuristic which can be applied to any feasible solution, either found during the branch-and-cut procedure or otherwise. It works analogously to the greedy start heuristic (algorithm 4), the only difference being that one is already starting with a feasible solution. In particular, the heuristic can be applied to solutions constructed by the receptor-terminal shortest path start heuristic (algorithm 5) described in the previous section. Trying to improve the greedy start heuristic (algorithm 4) with the improvement strategy outlined below is futile however since by construction the former already added all potential subgraph nodes in a greedy fashion. During a branch-and-cut run any new feasible solution can potentially be improved by the heuristic. In case of an incumbent one can hope for an even better incumbent, in case of a feasible solution one can hope to improve it up to a point where it actually becomes a new incumbent. The description of the heuristic is provided as algorithm 6.

Algorithm 5: Receptor-terminal shortest path start heuristic for the DeRegNet model

Input: DeRegNet instance with **Ex** = **Inc** = ∅
Output: Feasible solution of DeRegNet instance **or** *null*
if $R \neq \emptyset$ **then**
  $V_R = R$
else
  $V_R = V$
$r^* = \text{argmax}_{v \in V_R} s_v$ (Receptor with highest score)
if $T \neq \emptyset$ **then**
  $V_T = T$
else
  $V_T = V$
$t^* = \text{argmax}_{v \in V_T} s_v$ (Terminal with highest score)
$V^* = \{r^*, t^*\}$ (Selected DeRegNet solution)
$P =$ **SHORTEST_PATH**(G, r^*, t^*) (Find shortest path between receptor and terminal)
$V^* = V^* \cup P$ (Add nodes from shortest path)
if $k_{min} \leq |V^*| \leq k_{max}$ **then**
  return V^* (Return solution if it satisfies the subgraph size constraints)
else
  return *null* (Return nothing if size constraints are not met)

Algorithm 6: Greedy improvement heuristic for the DeRegNet model

Input: Feasible solution of a DeRegNet instance
Output: Another feasible solution of (the same) DeRegNet instance
$V^* = \{v \in V : x_v = 1\}$ (Selected DeRegNet solution)
$N = (\bigcup_{v \in V^*} \delta^+(v)) \setminus (V^* \cup \textbf{Ex})$ (Candidate nodes to be added next)
$A^* = \frac{s^T x}{e^T x}$ (Current average score of selected subgraph)
CONTINUE = **true**
while *CONTINUE and* $|V^*| < k_{max}$ **do**
  $v^* = \text{argmax}_{v \in N} s_v$ (Highest scoring node in candidate set)
  if $s_{v^*} \geq A^*$ **then**
    $A^* = \frac{|V^*|A^* + s_{v^*}}{|V^*|+1}$ (Update average score of selected subgraph)
    $V^* = V^* \cup \{v^*\}$ (Update current subgraphs)
    $N^* = (\delta^+(v^*) \setminus V^*) \setminus \textbf{Ex}$ (New candidate nodes)
    $N = (N \setminus \{v^*\}) \cup N^*$ (Update candidate nodes)
  else
    CONTINUE = **false**
return V^*

Approximate solutions via branch-and-bound gap cut

One can use a mixed-integer programming solver generically to obtain suboptimal solutions to a given (maximization) MILP with optimal objective value z^*. During the branch-and-cut search one obtains lower bounds on the optimal value by feasible solutions to the problem and an upper bound by the solution value of the initial LP relaxation of the problem. Let $\underline{z} \leq z^*$ be the best available lower bound and let $\bar{z} \geq z^*$ be the upper bound obtained by the relaxed problem. The *relative gap* λ_{rel} during a branch-and-cut search is defined as $\lambda_{rel} := \frac{z^*}{\bar{z}}$. With the upper bound $\hat{\lambda}_{rel} := \frac{\bar{z}}{\underline{z}} \geq \frac{z^*}{\bar{z}}$ on the gap it follows that $z^* \leq \hat{\lambda}_{rel}\underline{z}$ and hence $\alpha z^* \leq \underline{z}$ with $\alpha := \hat{\lambda}_{rel}^{-1}$. Stopping the branch-and-cut procedure at the given gap upper bound value hence provides an approximate solution of a posteriori approximation guarantee of $\hat{\lambda}_{rel}^{-1}$. I refer to the strategy of stopping the branch-and-cut search once the gap upper bound is below a certain threshold as *gap cut* or *gap (cut) thresholding*. Employing the gap cut strategy can be useful in situations where the MILP solver can find reasonably good solutions in reasonable time but would take significantly more time to find the optimal solution. The option of to carry out gap cut thresholding is incorporated in the implementation of DeRegNet for this very reason.

Caching transformed model formulations

For DeRegNet's use cases it is quite common to optimize DeRegNet instances which just differ in terms of their node scores, i.e. share the same underlying graph. For example, finding deregulated subgraphs for individual cases in a TCGA cohort with a fixed regulatory network derived from KEGG will require to solve a model with the same structural properties but with differing score data, for example a omics-readout for every case in the cohort. In particular, in such a situation the reformulation and linearization procedure of the generalized Charnes-Cooper transform only has to be carried out once and can be reused across cases since it does not depend structurally on the objective data vector s. While solution time of a DeRegNet instance with the generalized Charnes-Cooper transform tends to be dominated by the time to solve the resulting integer linear program, reuse of the transformed model structure can nonetheless result in significant computational savings.

Further details on benchmarking DeRegNet

Algorithm 7 details the benchmark instance simulation algorithm. Algorithm 8 details the mode of application of [1] in the context of the benchmarks described in the main part of the paper. Figure 1 depicts the subgraphs simulation procedure conceptually.

DeRegNet subgraph derived features for predicting survival

Predicting phenotypes based on clinical and molecular data is one of the big challenges on the road to personalized medicine. A frequently readily available phenotype for cancer patients is survival time (i.e. the time from disease onset/diagnosis

Algorithm 7: Simulating DeRegNet instances with known "optimal" subgraph. Here, **Ber**(p) denotes a Bernoulli random variable with parameter $p \in [0,1]$. Note that the true minimal subgraph sizes k^*_{min} and k^*_{max} can be different than the minimal and maximal subgraph sizes k_{min} and k_{max} specified when solving related DeRegNet or Backes et al. instances.

Input: A directed graph $G = (V, E)$, sets $T \subset V$, *in-subgraph deregulation probability* $p' \in (0,1]$, *out-subgraph deregulation probability* $p \in (0, p')$, true minimal subgraph size $k^*_{min} \in \mathbb{N}$, true maximal subgraph size $k^*_{max} \in \mathbb{N}$.
Output: A DeRegNet instance, a simulated *true* optimal subgraph $V' \subset V$ and the simulated root node r

```
Choose r ∈ R with probability 1/|R| (Choose root node)
V' := {r} (Initialize subgraph with root)
Choose subgraph size k ∈ [k*_min, k*_max] ∩ ℕ uniformly
while |V'| ≠ k do
    if ( ⋃_{v'∈V'} δ+(v)) \ V' = ∅ then
        RESTART Algorithm 7
    Choose v ∈ ( ⋃_{v'∈V'} δ+(v)) \ V' with probability |( ⋃_{v'∈V'} δ+(v)) \ V')|^-1
    V' = V' ∪ {v}
for v ∈ V' do
    Sample s(v) ~ Ber(p')
for v ∈ V \ V' do
    Sample s(v) ~ Ber(p)
return (G, R, ∅, ∅, ∅, s), V', r
```

Algorithm 8: Applying [1] for benchmarking DeRegNet. Here, **APPLY_BACKES**(k) refers to applying the algorithm of [1] with fixed subgraph size k, understood to return a set of nodes corresponding to the induced subgraph found by the run.

Input: A DeRegNet instance with underlying graph $G = (V, E)$
Output: A set $V' \subset V$ (inducing a subgraph)

```
V' := ∅ (Initialize the final subgraph)
for k = k_min; k ≤ k_max; k++ do
    V' = V' ∪ APPLY_BACKES(k)
end
return V'
```

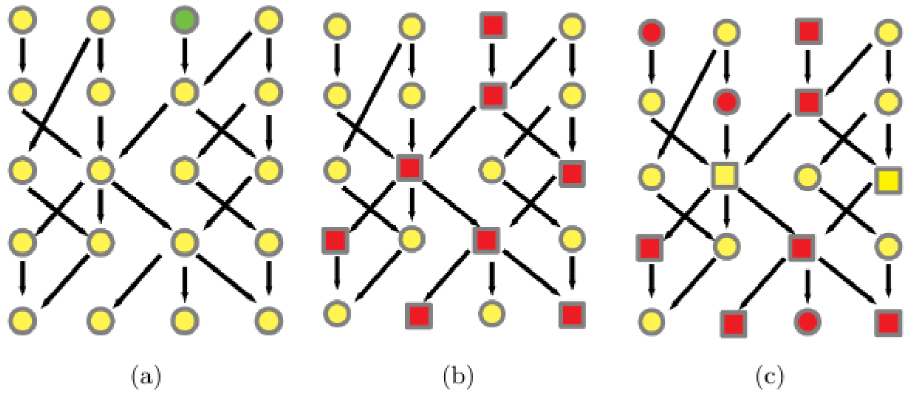

Figure A 1: **Simulating DeRegNet instances.** See algorithm 7 for a formal outline of the simulation procedure. On a high level it proceeds like this: **(a)** Choose root node randomly. **(b)** Simulate a feasible subgraph by randomly choosing nodes maintaining the topological constraints of the model. Set the deregulation score of nodes in the subgraph to one. **(c)** Introduce noise by flipping node deregulation scores randomly.

to (possibly disease induced) death). Improving upon clinical predictors with molecular data often still poses significant challenges [35]. Here, we provide an example of the suitability of deregulated subgraph-derived features for predicting survival in the TCGA-LIHC dataset. In particular, we demonstrate that predictions based on subgraphs is at least as good GSEA-based predictions obtained in a comparable manner. Furthermore, subgraph derived features can improve upon predictions based on clinical features alone.

Data preparation and feature engineering

Survival times were binarized by labeling all patients with survival less than three years (1095 days) as bad outlook patients ($y = 0$) and all patients with last follow-up time larger than three years as good outlook patients ($y = 1$). The resulting dataset consisted of 198 patients from the TCGA-LIHC cohort. For every case the following features are derived:

- **clinical**: Features from clinical data comprising *age*, *gender*, *body mass index (BMI)*, *tumor stage* (!) and *tumor morphology*. Age (in years) and BMI were scaled via z-scores. Tumor stage and morphology where one-hot encoded.
- **gsea**: Features derived from (single sample) Gene Set Enrichment Analysis (GSEA) [36]. Two lists of significantly enriched pathways w.r.t good outcomes vs. bad outcomes and vice versa were computed by (standard) GSEA. From every list I retained pathways with adjusted p-value less than 0.1, which resulted in a total of 14 KEGG pathways. After performing ssGSEA, every sample received the corresponding personalized ssGSEA enrichment scores for these pathways as a 14-dimensional feature vector. The above steps were carried out with *gseapy* (`http://gseapy.rtfd.io/`). For more information on single-sample GSEA, see [37]. The obtained features were scaled via z-scores.

- **subgraph_overlap**: Features based on up- and downregulated subgraphs for the good and bad outcome subgroups. Subgraphs were computed based on the global deregulation score for the good outcome and bad outcome patients respectively (on the respective training sets only, see below). Every sample is then associated with the regulation-aware node overlap between its personalized de-, up- and downregulated subgraphs and up- and downregulated global subgraphs for the good and bad outcome subgroups respectively. The deregulation-aware node overlap is defined as follows. Given two (induced) subgraphs $V', V'' \subset V$ and node scores $s', s'' : V \to \{-1, 0, 1\}$ the deregulation-aware node overlap is defined as $\sum_{v \in V} (\mathbb{I}(v \in V')\ s'_v) \cdot (\mathbb{I}(v \in V'')\ s''_v)$. This amounts to a 12-dimensional feature vector. Again, z-scores were applied.
- **ndcg**: Subgraph features derived from network-defined cancer genes. After identifying network-defined cancer genes (see previous subsection) for de-, up- and downregulated subgraphs one obtains a binary indicator for every case representing whether it contains any given such gene or not, leading to 15-dimensional feature vectors corresponding to 15 network-defined cancer genes.
- **subgraph**: *subgraph_overlap* and *ndcg* combined (concatenated).

Under a *feature combination* it is understood the combination of two or more of the just defined features. In the following, I use a plus sign to indicate feature combinations, e.g. $subgraph = subgraph\_overlap + ndcg$. As another example, $subgraph + clinical$ then denotes *subgraph* features combined with *clinical* features.

Survival prediction with clinical, pathway and subgraph features

The experiments described in the following were carried out with scikit-learn (https://scikit-learn.org). Every feature/feature combination was tested by training a Support Vector Machine, a simple artificial neural network, a random forest and a logistic regression. For every algorithm we performed an algorithm-specific grid search for model selection. The grid search was equivalent for different feature combinations in order to be able to assess the comparative suitability of the features. Final models were evaluated with 6-fold cross validation estimating mean Receiver Operating Characteristic (ROC) curves and Area under the curve (AUC) scores.

Features *gsea* and *subgraph_overlap* are roughly equivalent with respect to the underlying logic, with subgraphs or pathways as contextual data inputs respectively. Hence, comparing these two features may give an indication of the suitability of subgraph vs. pathway methods for feature engineering for survival prediction. Figure 2 shows that the *subgraph_overlap* features hold promise w.r.t *gsea* features.

Furthermore, it has been shown that improving upon clinical features with molecular features for survival prediction is not an easy task [35]. The experiments conducted here show that for the given setting, prediction models combining clinical and subgraph features (based on molecular interactions and data) provide performance gains compared to a purely clinical model. Also, the subgraph features achieve parity with classifiers based on clinical data alone. Figure 3 represents these findings.

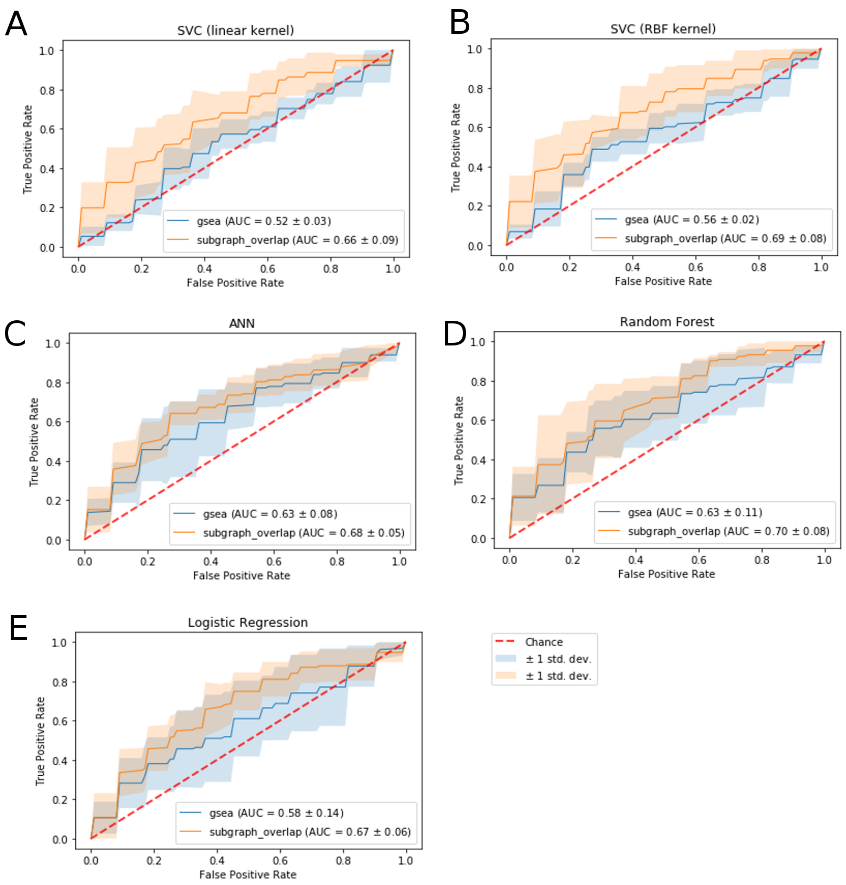

Figure A 2: **Subgraph features vs. GSEA features across models.** (A) Support Vector Classifier (SVC) with linear kernel (B) Support Vector Classifier (SVC) with radial basis function (RBF) kernel (C) Artificial Neural Network (ANN) (D) Random forest (E) Logistic Regression.

Author details

[1]Applied Bioinformatics, Dept. of Computer Science, University of Tuebingen, Tuebingen, Germany. [2]International Max Planck Research School (IMPRS) "From Molecules to Organisms", Tuebingen, Germany. [3]Interfaculty Institute for Cell Biology (IFIZ), University of Tuebingen, Tuebingen, Germany. [4]German Cancer Consortium (DKTK), German Cancer Research Center (DKFZ), Heidelberg, Germany. [5]Leibniz Institute on Aging (FLI), Jena, Germany. [6]Institute for Bioinformatics and Medical Informatics, University of Tuebingen, Tuebingen, Germany. [7]Translational Bioinformatics, University Hospital Tuebingen, Tuebingen, Germany.

References

1. Backes, C., Rurainski, A., Klau, G.W., Muller, O., Stockel, D., Gerasch, A., Kuntzer, J., Maisel, D., Ludwig, N., Hein, M., Keller, A., Burtscher, H., Kaufmann, M., Meese, E., Lenhof, H.P.: An integer linear programming approach for finding deregulated subgraphs in regulatory networks. Nucleic Acids Res. **40**(6), 43 (2012)
2. Dittrich, M.T., Klau, G.W., Rosenwald, A., Dandekar, T., Muller, T.: Identifying functional modules in protein-protein interaction networks: an integrated exact approach. Bioinformatics **24**(13), 223–231 (2008)
3. Buchanan, A., Wang, Y., Butenko, S.: Algorithms for node-weighted steiner tree and maximum-weight connected subgraph. Networks **72** (2017). doi:10.1002/net.21825
4. Loboda, A.A., Artyomov, M.N., Sergushichev, A.A.: Solving generalized maximum-weight connected subgraph problem for network enrichment analysis. In: Frith, M., Storm Pedersen, C.N. (eds.) Algorithms in Bioinformatics, pp. 210–221. Springer, Cham (2016)
5. El-Kebir, M., Klau, G.: Solving the maximum-weight connected subgraph problem to optimality. 11th DIMACS implementation challenge (2014)
6. Alvarez-Miranda, E., Ljubic, I., Mutzel, P.: The Maximum Weight Connected Subgraph Problem. In: Juenger, M., Reinelt, G. (eds.) The Maximum Weight Connected Subgraph Problem, pp. 245–270. Springer, Berlin,

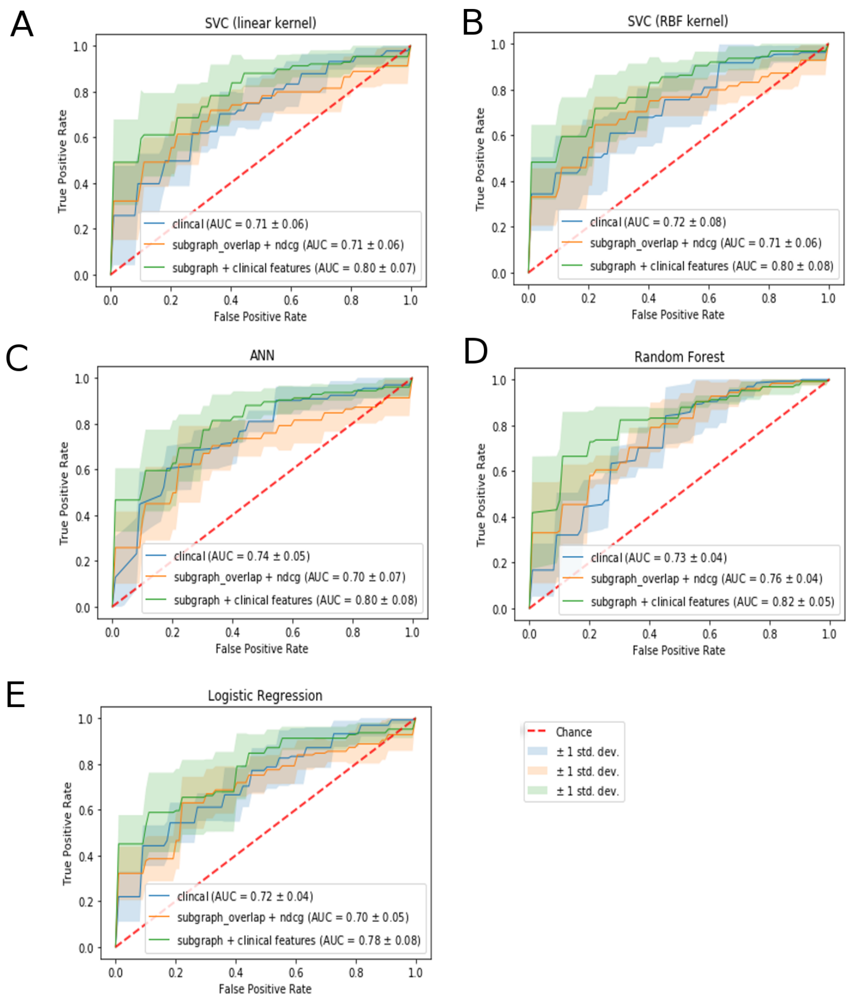

Figure A 3: **Subgraph features vs. clinical features across models.** Clinical + subgraph features together outperform either in isolation. Subgraph features perform comparably to clinical features. (A) Support Vector Classifier (SVC) with linear kernel (B) Support Vector Classifier (SVC) with radial basis function (RBF) kernel (C) Artificial Neural Network (ANN) (D) Random forest (E) Logistic Regression.

Heidelberg (2013)

7. Álvarez-Miranda, E., Ljubić, I., Mutzel, P.: The rooted maximum node-weight connected subgraph problem. In: Gomes, C., Sellmann, M. (eds.) Integration of AI and OR Techniques in Constraint Programming for Combinatorial Optimization Problems, pp. 300–315. Springer, Berlin, Heidelberg (2013)
8. Althaus, E., Blumenstock, M.: Algorithms for the maximum weight connected subgraph and prize-collecting steiner tree problems. 11th DIMACS Implementation Challenge in Collaboration with ICERM (2011)
9. Álvarez-Miranda, E., Sinnl, M.: A relax-and-cut framework for large-scale maximum weight connected subgraph problems. Computers & Operations Research **87**, 63–82 (2017). doi:10.1016/j.cor.2017.05.015
10. Rehfeldt, D., Koch, T., Maher, S.J.: Reduction techniques for the prize collecting steiner tree problem and the maximum-weight connected subgraph problem. Networks **73**(2), 206–233 (2019). doi:10.1002/net.21857. https://onlinelibrary.wiley.com/doi/pdf/10.1002/net.21857
11. Rehfeldt, D., Koch, T.: Combining np-hard reduction techniques and strong heuristics in an exact algorithm for the maximum-weight connected subgraph problem. SIAM Journal on Optimization **29**(1), 369–398 (2019). doi:10.1137/17M1145963. https://doi.org/10.1137/17M1145963
12. You, F., Castro, P.M., Grossmann, I.E.: Dinkelbach's algorithm as an efficient method to solve a class of minlp

models for large-scale cyclic scheduling problems. Computers & Chemical Engineering **33**, 1879–1889 (2009)
13. Yue, D., Guillén-Gosálbez, G., You, F.: Global optimization of large-scale mixed-integer linear fractional programming problems: a reformulation-linearization method and process scheduling applications. AIChE Journal **59**(11), 4255–4272 (2013)
14. Charnes, A., Cooper, W.W.: Programming with linear fractional functionals. Naval Research Logistics Quaterly **9**, 181–186 (1962)
15. Dinkelbach, W.: Die maximierung eines quotienten zweier linearer funktionen unter linearen nebenbedingungen. Z. Wahrscheinlichkeitstheorie **1**, 141–145 (1962)
16. Dinkelbach, W.: On nonlinear fractional programming. Managment Science **13**(7), 492–498 (1967)
17. Anzai, Y.: On integer fractional programming. J. Operations Research Soc. of Japan **17**(1), 49–66 (1974)
18. Adams, W.P., Forrester, R.J., Glover, F.: Comparison and enhancement strategies for linearizing mixed 0-1 quadratic programs. Discrete Optimization **1**, 99–120 (2004)
19. Adams, W.P., Forrester, R.J.: A simple recipe for concise mixed 0-1 linearizations. Operations Research Letters **33**, 55–61 (2005)
20. Glover, F.: Improved linear integer programming formulations of nonlinear integer problems. Managment Science **22**(4), 455–460 (1975)
21. Conforti, M., Cornuéjols, G., Zanbelli, G.: Integer Programming. Springer, ??? (2014)
22. Sharir, M.: A strong-connectivity algorithm and its applications to data flow analysis. Computers and Mathematics with applications **7**(1), 67–72 (1981)
23. Tarjan, R.: Depth-first search and linear graph algorithms. SIAM Journal on Computing **1**(2), 146–160 (1972)
24. Dijkstra, E.W.: A Discipline of Programming. Prentice-Hall, ??? (1972)
25. Berthold, T.: Primal heuristics for mixed integer programs. PhD thesis, Technische Universität Berlin (2006)
26. Glover, F., M., L.: General purpose heuristics forinteger pro-gramming - part i. Journal of Heuristics **2**, 343–358 (1997)
27. Glover, F., M., L.: General purpose heuristics forinteger pro-gramming - part ii. Journal of Heuristics **3**, 161–179 (1997)
28. Fischetti, M., Glover, F., A., L.: The feasibility pump. Mathematical Programming **104**, 91–104 (2005)
29. Balas, E., Schmieta, S., Wallace, C.: Pivot and shift - amixed integerprogramming heuristic. Discrete Optimization **1**, 3–12 (2004)
30. Balas, E., Martin, C.H.: Pivot-and-complement: A heuristic for 0-1 programming. Management science **26**, 86–96 (1980)
31. Dijkstra, E.W.: A note on two problems in connexion with graphs. Numerische Mathematik **1**, 269–271 (1959)
32. Johnson, D.B.: Efficient algorithms for shortest paths in sparse networks. Journal of the ACM **24**(1) (1977)
33. Ahuja, R.K., Mehlhorn, K., Orlin, J., Tarjan, R.E.: Faster algorithms for the shortest path problem. Journal of the ACM **37**(2) (1990)
34. Taccari, L.: Integer programming formulations for the elementary shortest path problem. European Journal of Operational Research **252**(1) (2016)
35. Yuan, Y., Van Allen, E.M., Omberg, L., Wagle, N., Amin-Mansour, A., Sokolov, A., Byers, L.A., Xu, Y., Hess, K.R., Diao, L., Han, L., Huang, X., Lawrence, M.S., Weinstein, J.N., Stuart, J.M., Mills, G.B., Garraway, L.A., Margolin, A.A., Getz, G., Liang, H.: Assessing the clinical utility of cancer genomic and proteomic data across tumor types. Nat. Biotechnol. **32**(7), 644–652 (2014)
36. Subramanian, A., Tamayo, P., Mootha, V.K., Mukherjee, S., Ebert, B.L., Gillette, M.A., Paulovich, A., Pomeroy, S.L., Golub, T.R., Lander, E.S., Mesirov, J.P.: Gene set enrichment analysis: a knowledge-based approach for interpreting genome-wide expression profiles. Proc. Natl. Acad. Sci. U.S.A. **102**(43), 15545–15550 (2005)
37. Foroutan, M., Bhuva, D.D., Lyu, R., Horan, K., Cursons, J., Davis, M.J.: Single sample scoring of molecular phenotypes. BMC Bioinformatics **19**(1), 404 (2018)

Abbreviations

Acronyms

ANN Artificial Neural Network. 30, 31
AUC Area under the curve. 29

BMI Body Mass Index. 28

GSEA Gene set enrichment analysis. 28, 30

KEGG Kyoto Encyclopedia of Genes and Genomes. 26, 28

LIHC Liver Hepatocellular Carcinoma. 28

MAWCSP Maximum Average Weight Connected Subgraph Problem. 9
MWCSP Maximum Weight Connected Subgraph Problem. 8

RBF Radial Basis Function. 30, 31
RMAWCSP Rooted Maximum Average Weight Connected Subgraph Problem. 9
RMWCSP Rooted Maximum Weight Connected Subgraph Problem. 8
ROC Receiver Operating Characteristic. 29

ssGSEA Single Sample Gene Set Enrichment Analysis. 28
SVC Support Vector Classifier. 30, 31

TCGA The Cancer Genome Atlas. 26, 28

w.r.t with respect to. 28, 29
