# Supplementary material for: De novo identification of maximally deregulated subnetworks based on multi-omics data with DeRegNet
Source: BMC Bioinformatics. 2022 Apr 19;23:139. doi: 10.1186/s12859-022-04670-6 (PMC9020058; doi:10.1186/s12859-022-04670-6)
Supplement: Supplementary file 2 — Additional file 2: Supplementary Figures. This document contains supplementary figures associated to the main text. [file 12859_2022_4670_MOESM2_ESM.pdf]

Supplementary Figures

February 25, 2022

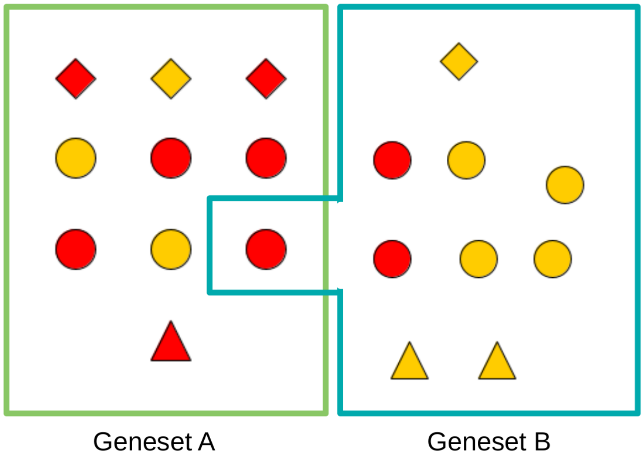

Figure S 1: **Conceptual view of classical pathway/gene set analysis.** Gene sets/pathways are considered merely as sets of genes ignoring any explicit biomolecular interactions between the elements of a gene set/pathway. Here red nodes represent differentially regulated genes and a basic GSE analysis employing hypergeometric Over-representation analysis (ORA) would test for more red nodes than expected in any given gene set.

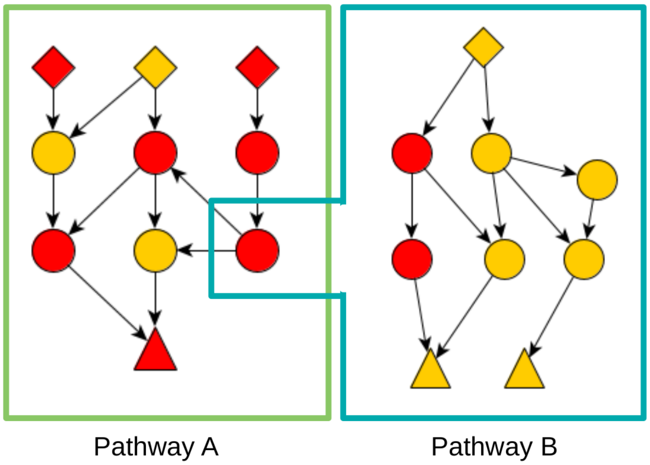

Figure S 2: **Conceptual view of topological pathway/analysis.** Biomolecular interaction are taken into account when calculating enrichment for any given pathway. Gene sets/pathways are still predefined though and interactions between pathways are usually not taken into account

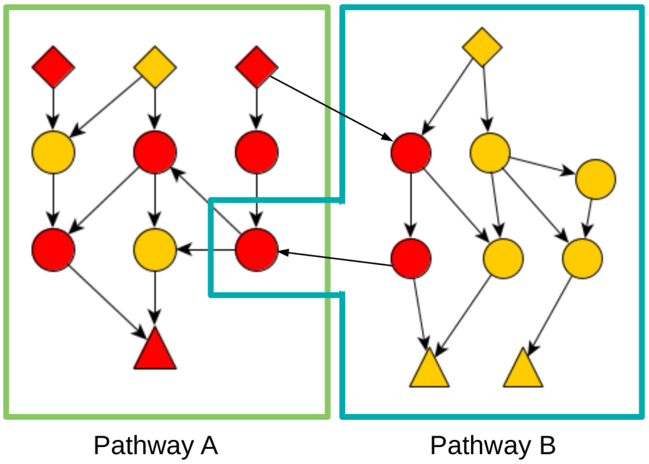

Figure S 3: **Conceptual view of topological pathway/analysis with pathway crosstalks.** Pathway crosstalks happen when genes are part of multiple pathways. They can also happen if there are genes in two pathways with interactions between them from another pathway. Even with pathway crosstalks accounted for, the gene sets/pathways as such are still predetermined.

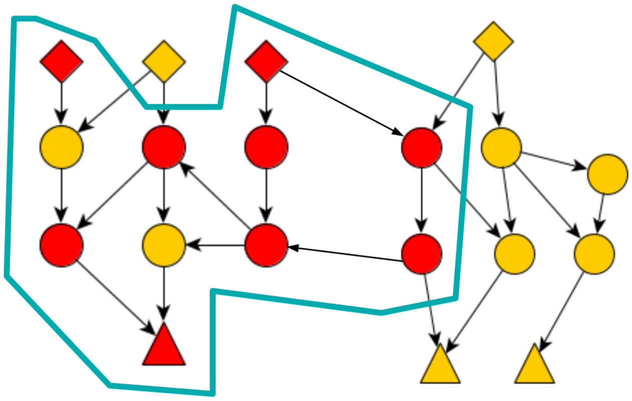

Figure S 4: **Conceptual view of de-novo pathway analysis.** De-novo pathway identification / deregulated subnetwork discovery drops the predetermined pathways and defines enriched subnetworks/pathways from the omics data itself.

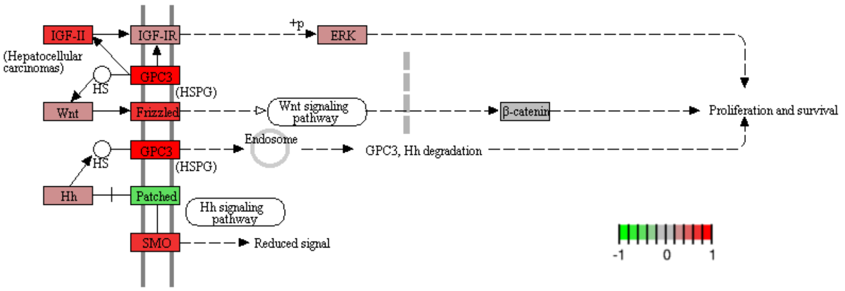

Figure S 5: **GPC3-mediated activation of WNT signaling is a well-documented process in liver cancer. The figure shows the relevent KEGG map (Proteoglycans in cancer: hsa05205; see https://www.kegg.jp/kegg/) with TCGA-LIHC min-max-scaled $\log_2$ fold changes mapped onto the genes. This process was automatically recaptured by our up-regulated subgraphs for TCGA-LIHC.**

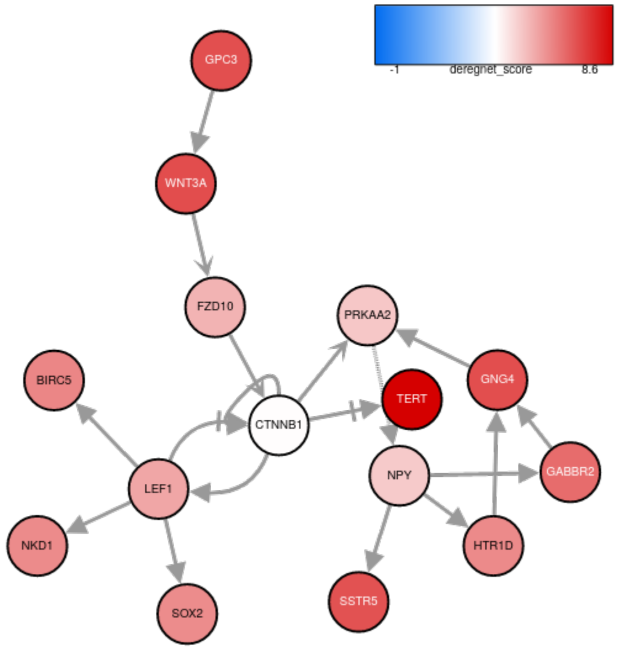

Figure S 6: **Optimal upregulated global subgraph for TCGA-LIHC**

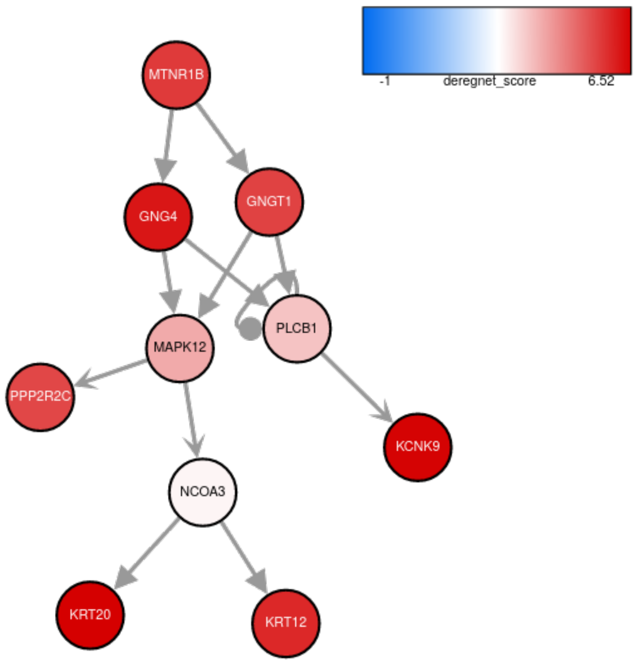

Figure S 7: 1st **suboptimal upregulated global subgraph for TCGA-LIHC**

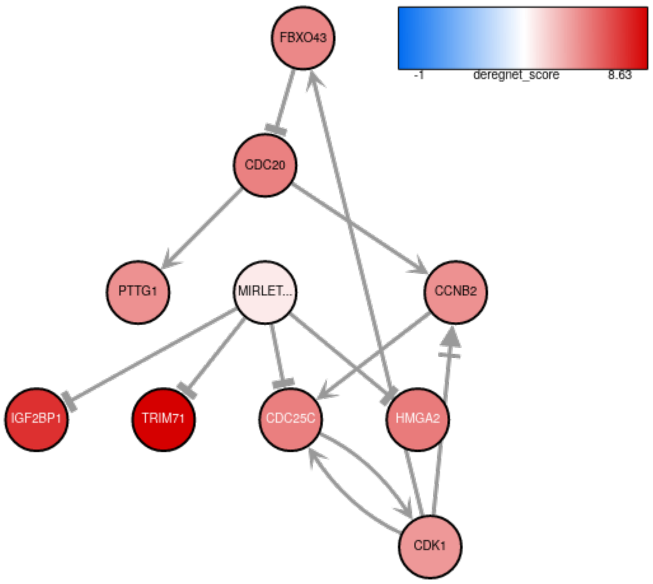

Figure S 8: 2nd **suboptimal upregulated global subgraph for TCGA-LIHC**

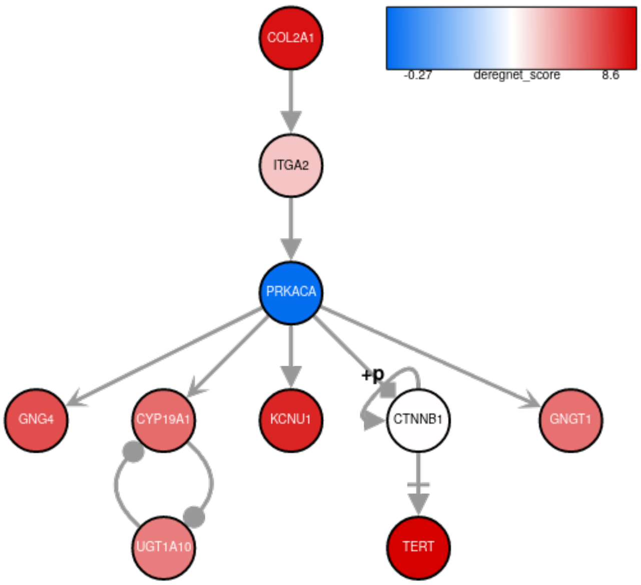

Figure S 9: 3rd **suboptimal upregulated global subgraph for TCGA-LIHC**

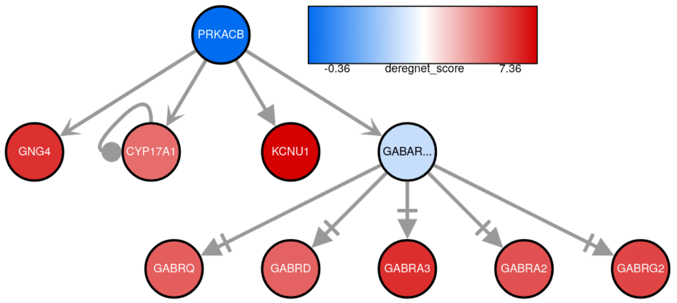

Figure S 10: 4th **suboptimal upregulated global subgraph for TCGA-LIHC**

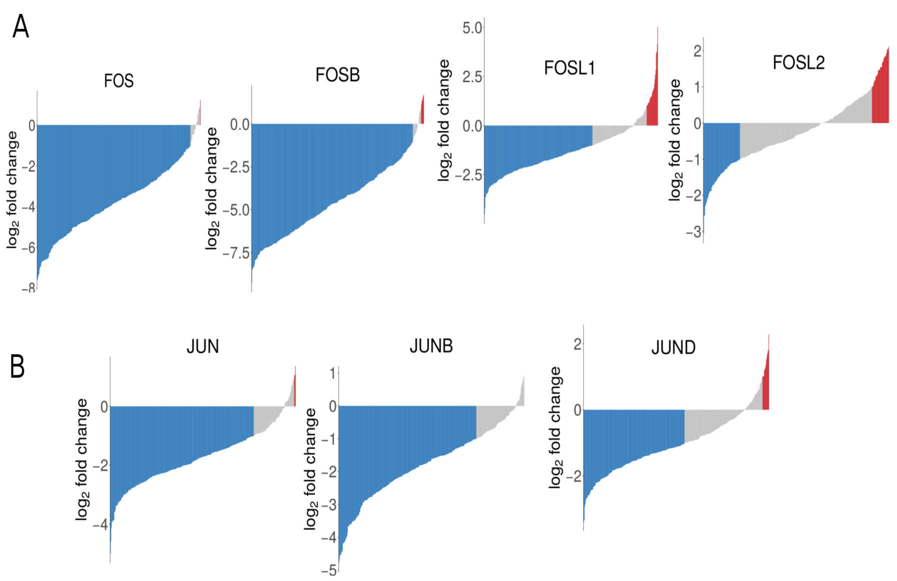

Figure S 11: **Expression of FOS and JUN isoforms in tumor of TCGA-LIHC cohort.** (A) Log_2-fold changes of FOS isoforms in individual tumors compared to the mean control value of the TCGA-LIHC dataset. (B) Log_2 fold changes of JUN isoforms in individual tumors compared to the mean control value of the TCGA-LIHC dataset. Bars in waterfall plot indicate mRNA down-regulation $\geq$ 1.5-fold (blue),mRNA upregulation $\geq$ 1.5-fold (red).

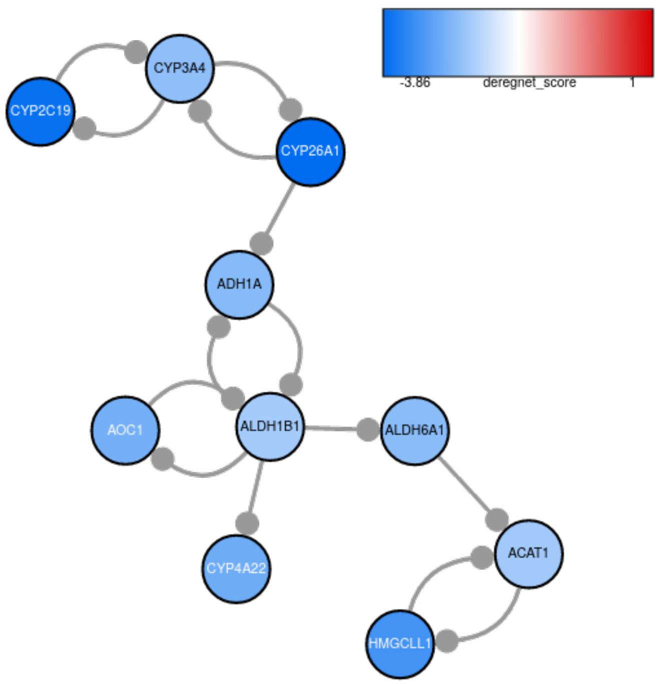

Figure S 12: **Optimal downregulated global subgraph for TCGA-LIHC**

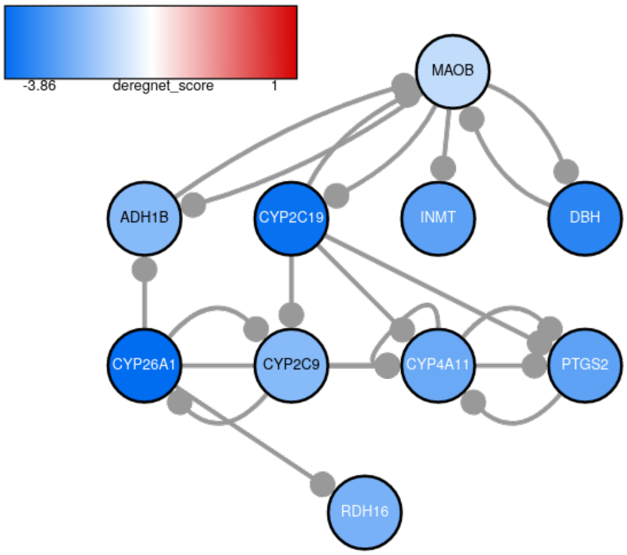

Figure S 13: 1st **suboptimal downregulated global subgraph for TCGA-LIHC**

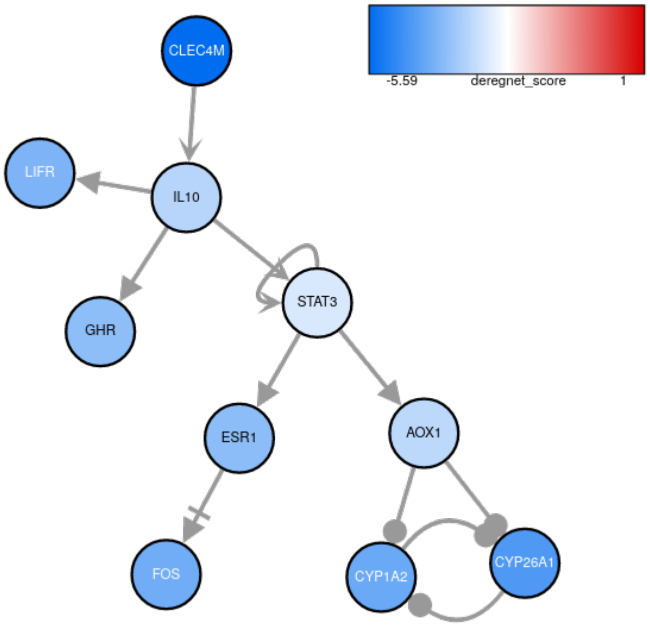

Figure S 14: 2nd **suboptimal downregulated global subgraph for TCGA-LIHC**

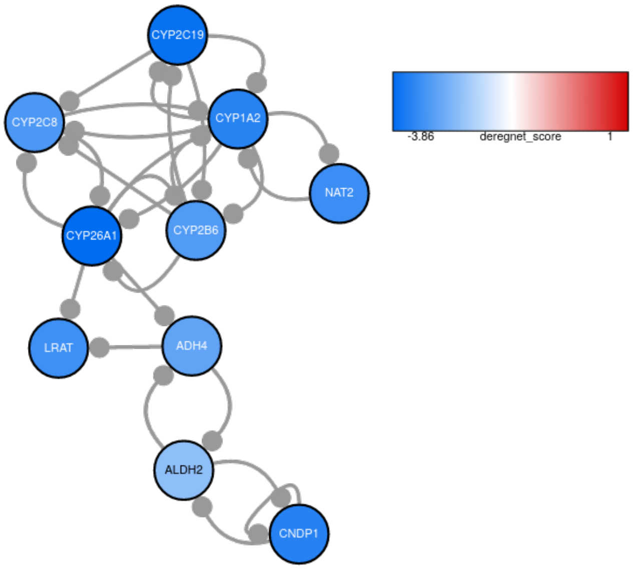

Figure S 15: 3rd **suboptimal downregulated global subgraph for TCGA-LIHC**

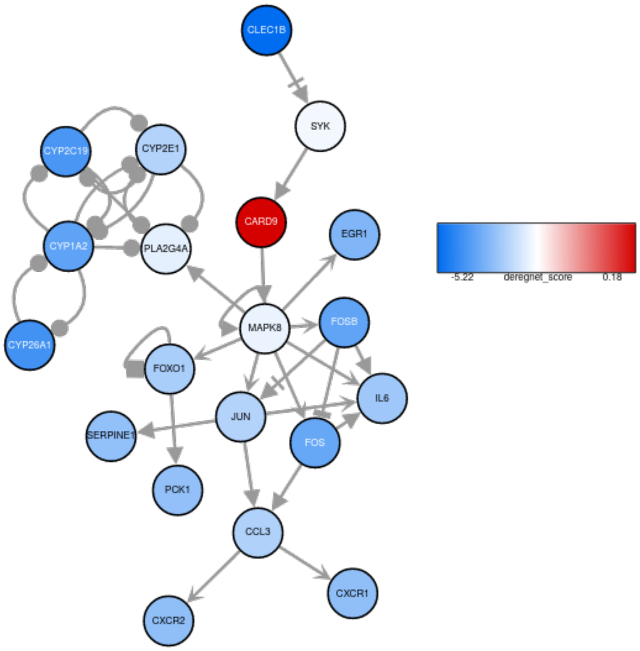

Figure S 16: 4th **suboptimal downregulated global subgraph for TCGA-LIHC**

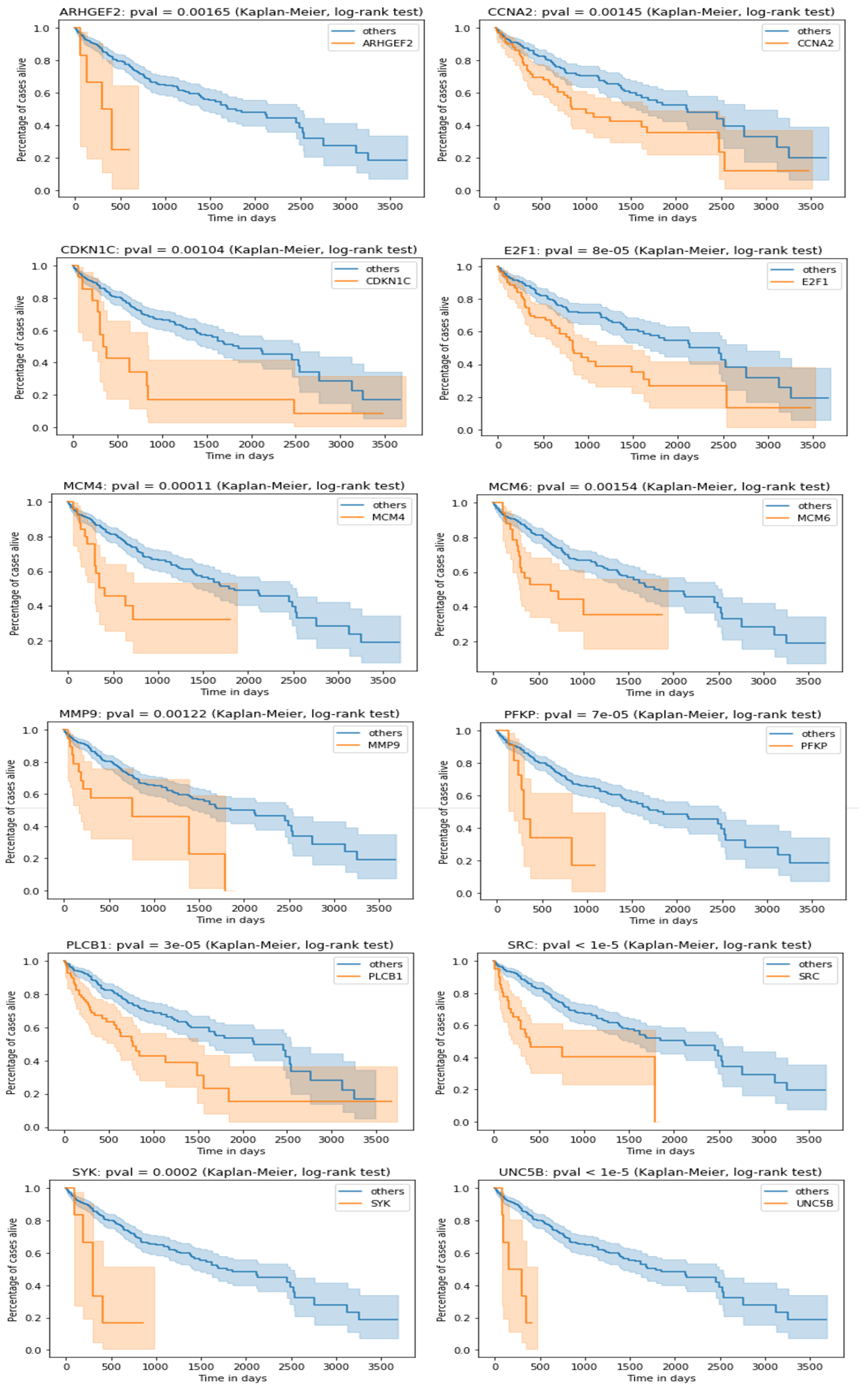

Figure S 17: **Subset of genes whose presence in a patient's inferred subgraph indicates a poor survival.** Survival difference is calculated using Kaplan-Meier estimates and log-rank test.

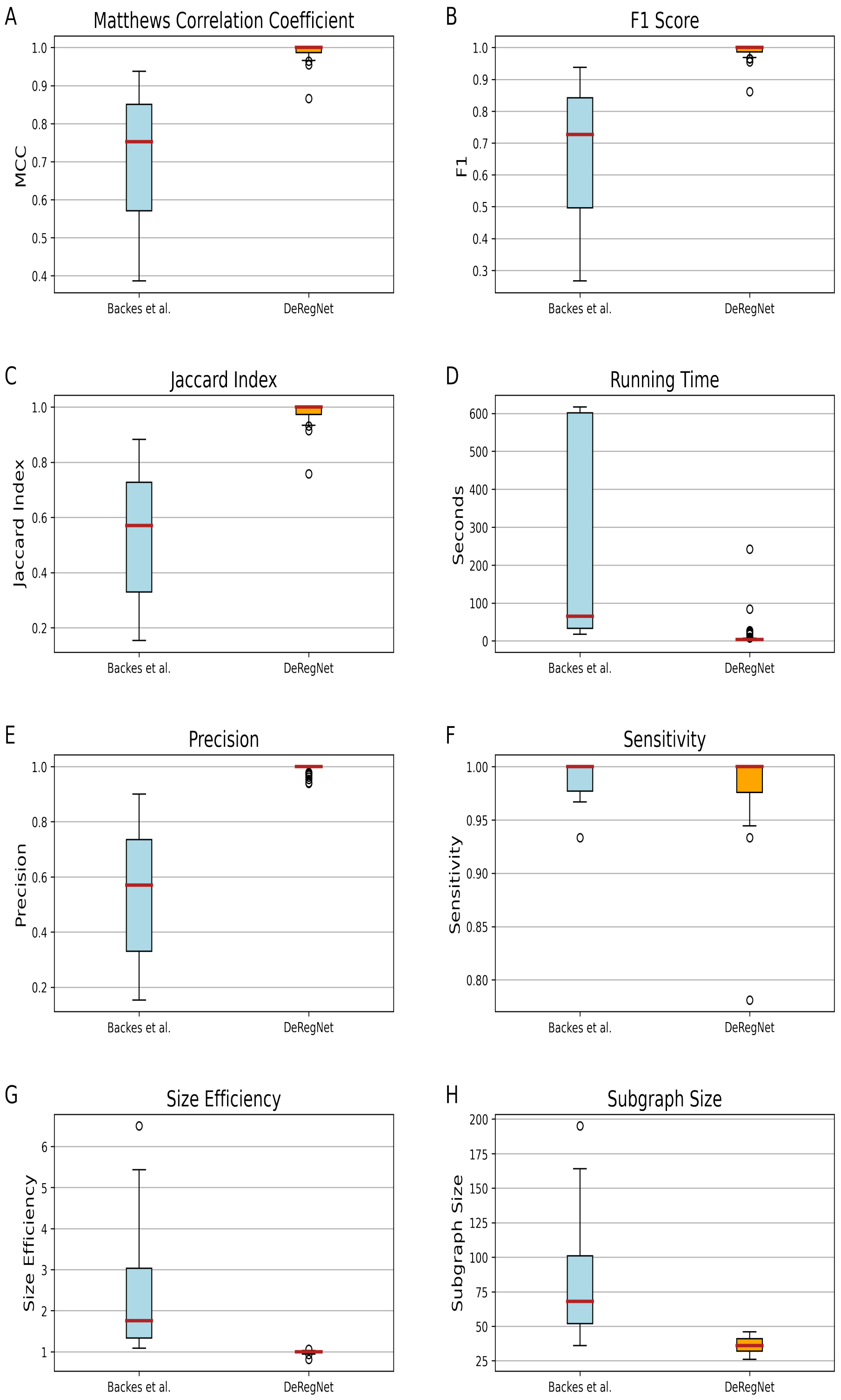

Figure S 18: **Benchmark results for out-of-subgraph deregulation probability** $p = 0.0005$**.** $k_{min} = 25$, $k_{max} = 50$, minimal size of simulated true subgraph $= 30$, maximal size of simulated true subgraph $= 45$, in-subgraph deregulation probability $p' = 0.99$, number of simulated instances $= 100$, time limit $= 600$ seconds.

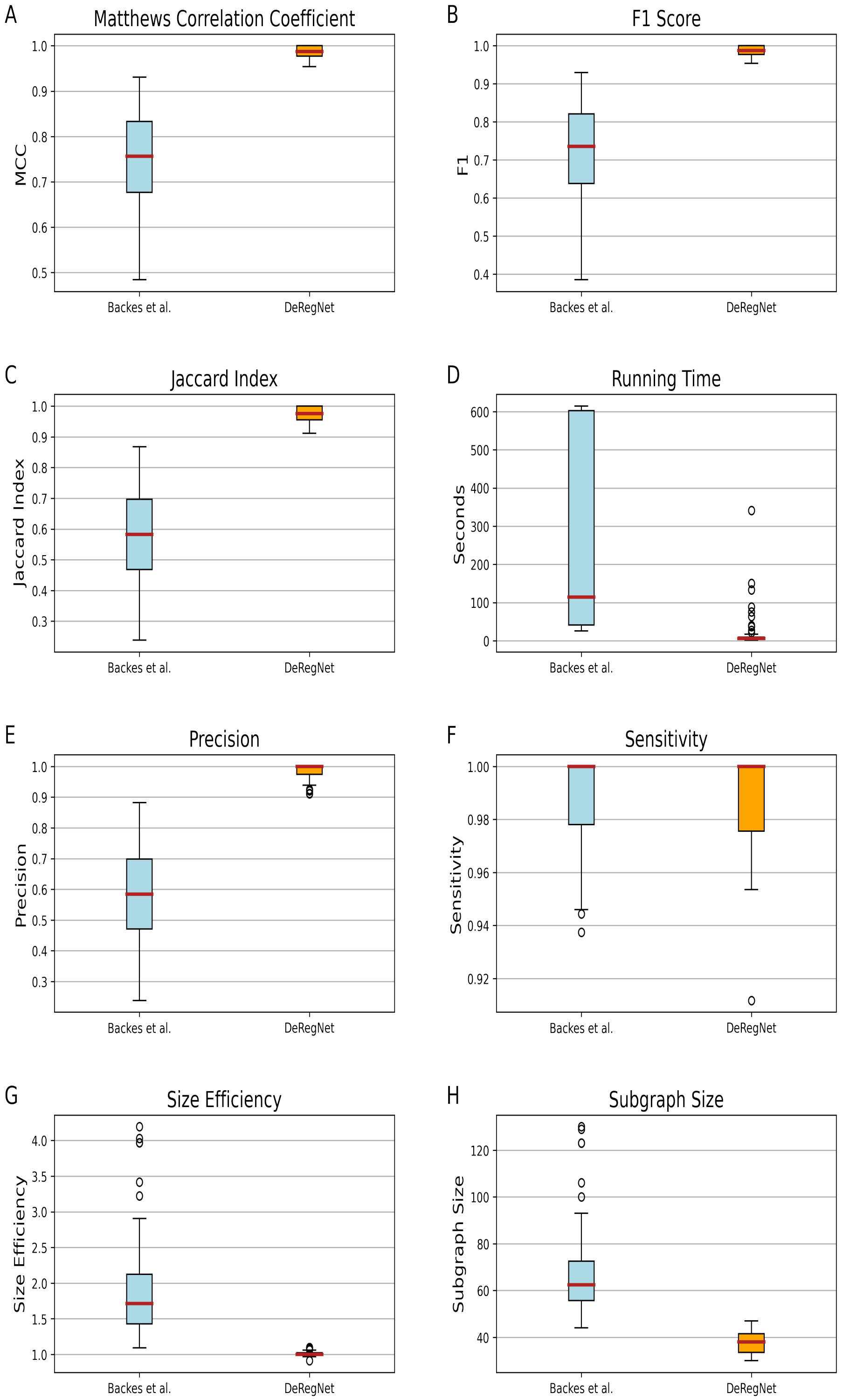

Figure S 19: **Benchmark results for out-of-subgraph deregulation probability $p = 0.001$.** $k_{min} = 25$, $k_{max} = 50$, minimal size of simulated true subgraph = 30, maximal size of simulated true subgraph = 45, in-subgraph deregulation probability $p' = 0.99$, number of simulated instances = 100, time limit = 600 seconds.

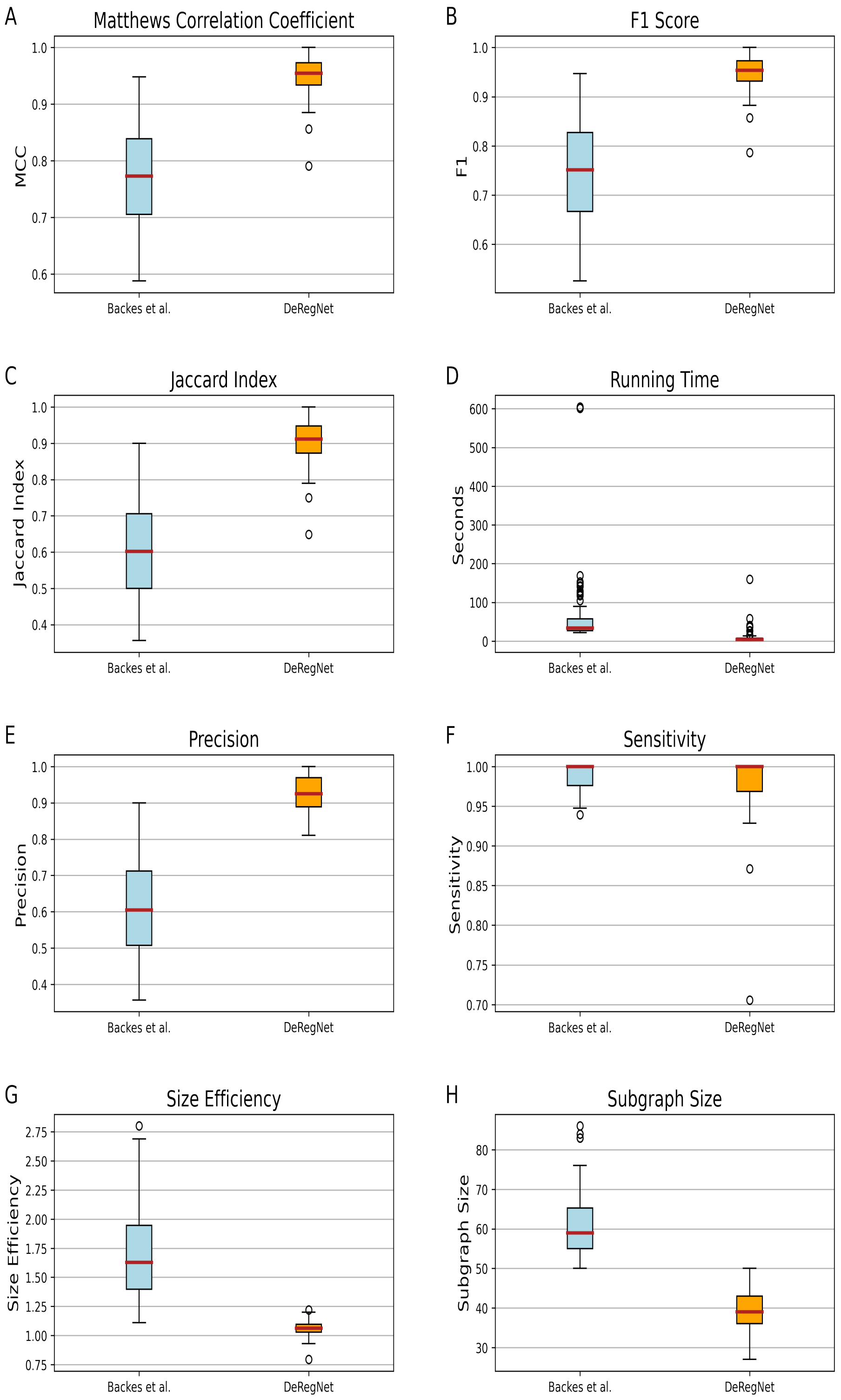

Figure S 20: **Benchmark results for out-of-subgraph deregulation probability $p = 0.005$.** $k_{min} = 25$, $k_{max} = 50$, minimal size of simulated true subgraph $= 30$, maximal size of simulated true subgraph $= 45$, in-subgraph deregulation probability $p' = 0.99$, number of simulated instances $= 100$, time limit $= 600$ seconds.

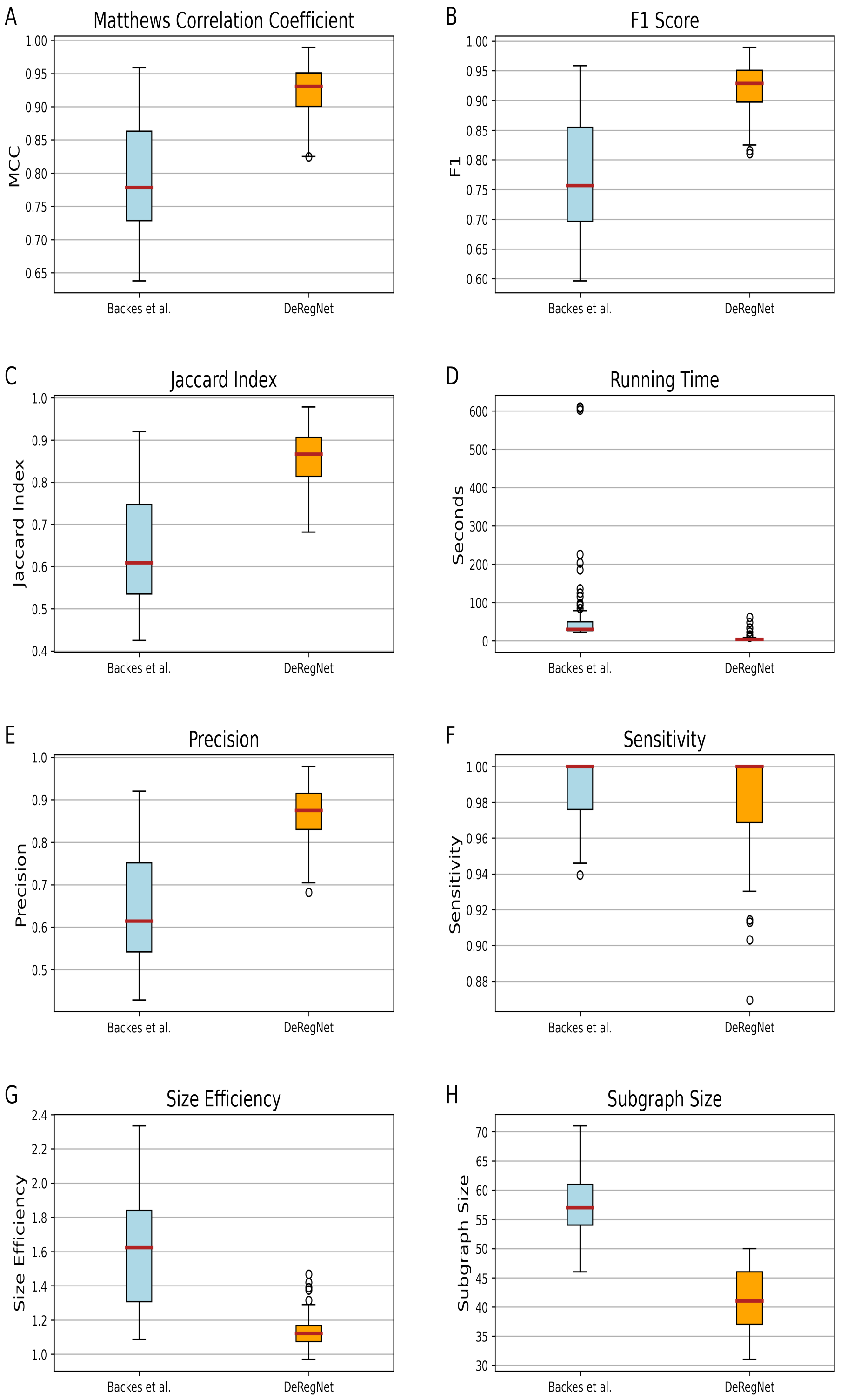

Figure S 21: **Benchmark results for out-of-subgraph deregulation probability $p = 0.01$.** $k_{min} = 25$, $k_{max} = 50$, minimal size of simulated true subgraph $= 30$, maximal size of simulated true subgraph $= 45$, in-subgraph deregulation probability $p' = 0.99$, number of simulated instances $= 100$, time limit $= 600$ seconds.

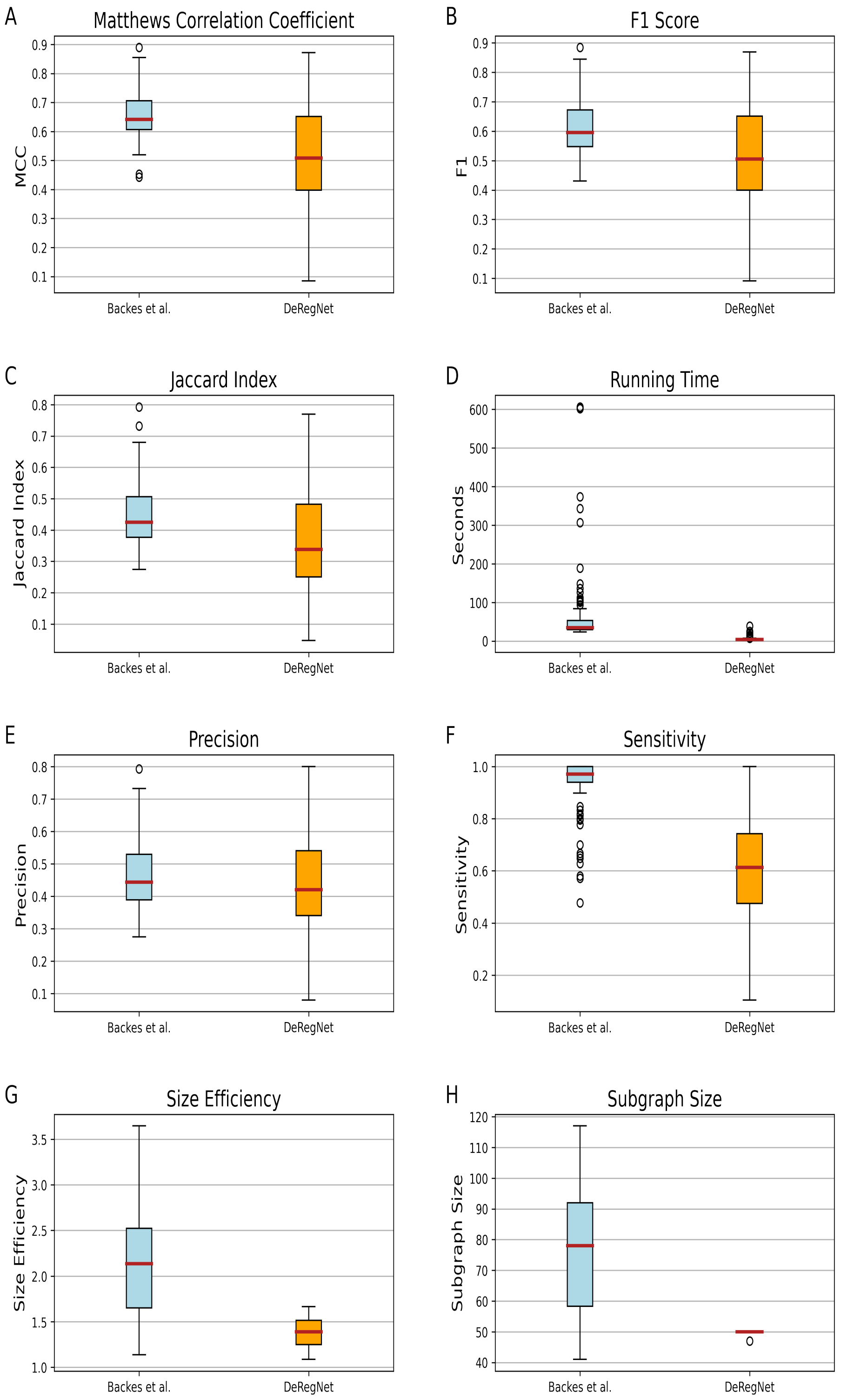

Figure S 22: **Benchmark results for out-of-subgraph deregulation probability** $p = 0.05$**.** $k_{min} = 25$, $k_{max} = 50$, minimal size of simulated true subgraph $= 30$, maximal size of simulated true subgraph $= 45$, in-subgraph deregulation probability $p' = 0.99$, number of simulated instances $= 100$, time limit $= 600$ seconds.

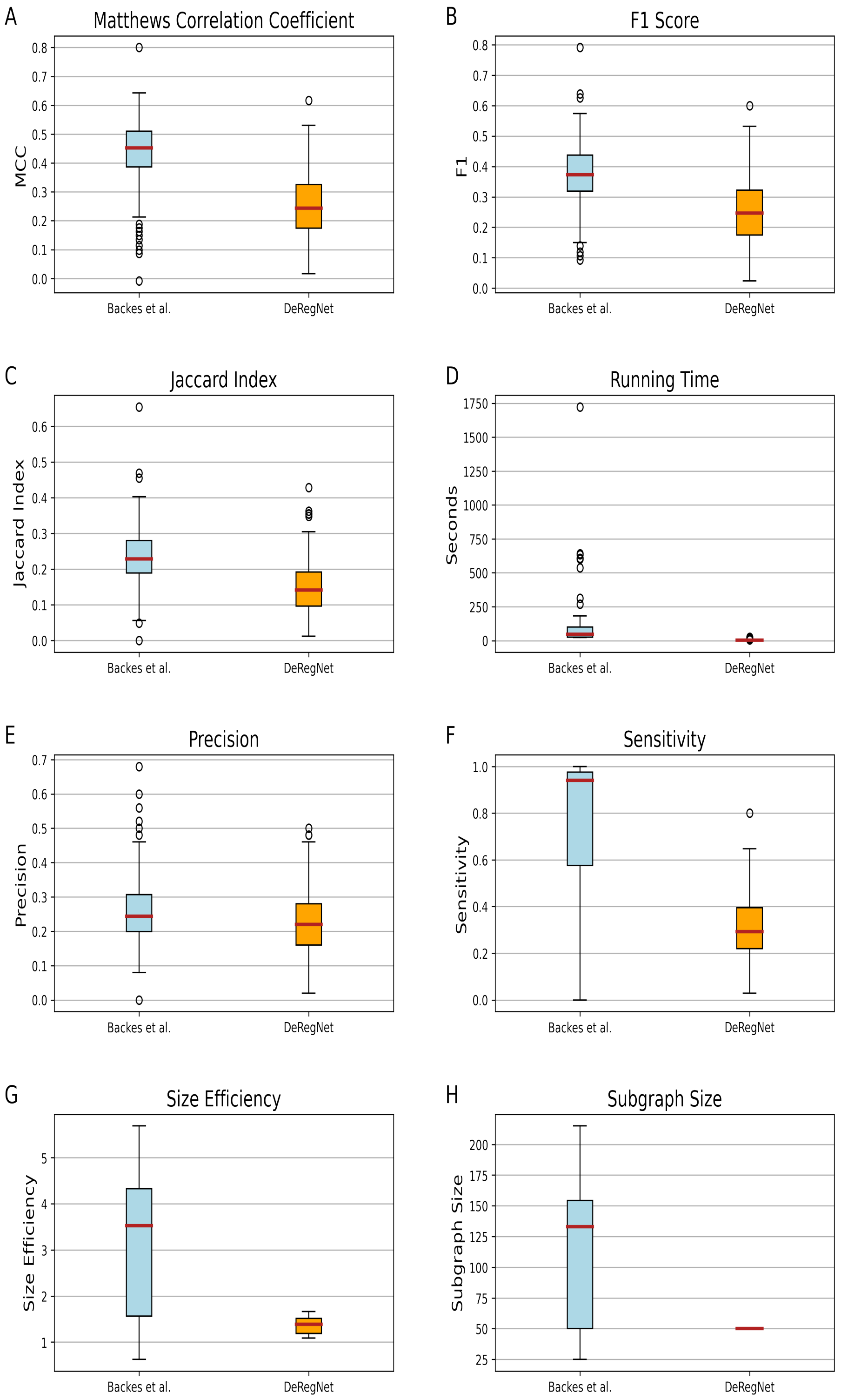

Figure S 23: **Benchmark results for out-of-subgraph deregulation probability $p = 0.1$.** $k_{min} = 25$, $k_{max} = 50$, minimal size of simulated true subgraph $= 30$, maximal size of simulated true subgraph $= 45$, in-subgraph deregulation probability $p' = 0.99$, number of simulated instances $= 100$, time limit $= 600$ seconds.

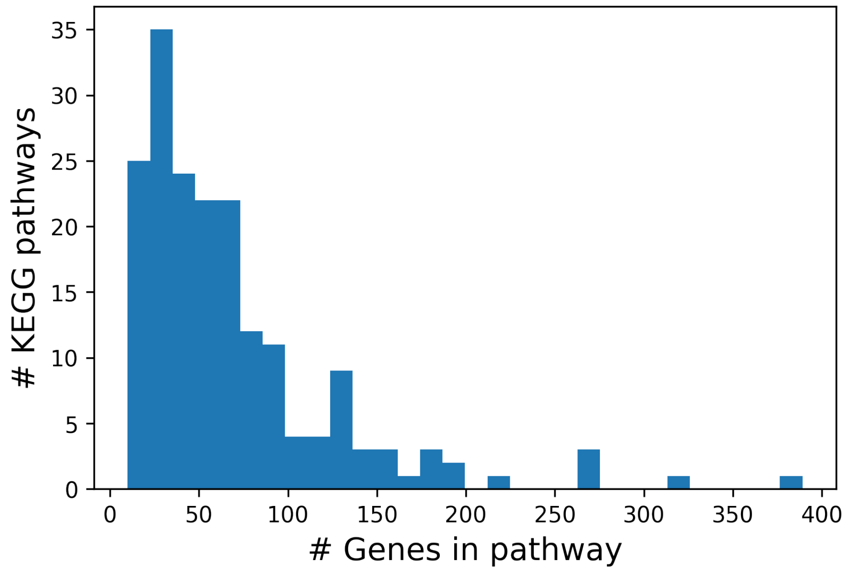

Figure S 24: **Distribution of KEGG pathway sizes.** As can be seen from the distribution pre-defined KEGG pathways have a median size around 50. Most pathways with much higher number of genes are in fact meta pathways representing a considerable extent of biomolecular function like functions related to cancer across cancer types for example. Most pathways which are more narrow/specific in scope are smaller and hence one option to choose k_{min} and k_{max} is to aim for subnetworks e.g. between 10 to 60 in order to aim for reasonably sized subnetworks given the median size of common pre-defined pathways.
